# B cells in anti-tRNA synthetase syndrome patients show an activated, interferon-responsive signature

**DOI:** 10.3389/fimmu.2026.1770291

**Published:** 2026-04-07

**Authors:** Erin M. Wilfong, Lindsay E. Bass, Leslie J. Crofford, Rachel H. Bonami, The MYSTIC Investigators

**Affiliations:** 1 Division of Rheumatology and Immunology, Department of Medicine, Vanderbilt University Medical Center, Nashville, TN, United States; 2 Division of Allergy, Pulmonary, and Critical Care Medicine, Department of Medicine, Vanderbilt University Medical Center, Nashville, TN, United States; 3 Vanderbilt Institute for Infection, Immunology, and Inflammation, Vanderbilt University Medical Center, Nashville, TN, United States; 4 Department of Pathology, Microbiology, and Immunology, Vanderbilt University Medical Center, Nashville, TN, United States

**Keywords:** antisynthetase syndrome, autoimmune diseases, autoimmunity, B lymphocytes, idiopathic inflammatory myopathies

## Abstract

**Introduction:**

The diagnostic value of autoantibodies together with the clinical utility of B cell-depleting therapies (e.g., rituximab) highlight a pathologic role for B cells in antisynthetase syndrome (ASyS). Mainstays of therapy however rely on broadly immunosuppressive agents, which often lead to incomplete treatment response. We therefore set out to identify dysregulated pathways in ASyS as novel therapeutic targets.

**Methods:**

Peripheral blood mononuclear nuclear cells were isolated from ASyS and healthy participants. Single-cell RNA sequencing was performed on flow sorted CD19+ cells, followed by differential gene expression and pathway analysis.

**Results:**

ASyS patients upregulated pathways related to either interferon or cellular stress (activated B cells) and interferon, actin, or chemical stress (memory B cells), with increased reactive oxygen species identified in several memory B cell subsets in ASyS participants. The frequency of memory B cells expressing the stress response gene, FKBP5 or lipid membrane raft organization gene, MYADM was higher in ASyS patients versus healthy controls. Pathway analysis of these memory subsets showed altered actin/cytoskeleton rearrangement, cellular stress response, and cellular metabolism (FKBP5+ memory) and altered antigen processing/presentation, cellular adhesion, and cell homing (MYADM+ memory) in ASyS.

**Discussion:**

Overall, our data identify novel and known gene expression changes within activated and memory B cells. These data implicate activated and memory B cell rewiring in ASyS that may support their ability to act as antigen-presenting cells. Future studies will be required to validate these findings and probe their utility as new targets to limit tissue damage in ASyS.

## 1 Introduction

Anti-synthetase syndrome (ASyS) is an autoimmune condition hallmarked by autoantibodies against tRNA synthetases. Clinically, ASyS can manifest with interstitial lung disease, skin rashes, arthritis, and muscle inflammation, although there is significant heterogeneity amongst patients. The anti-CD20 therapy, rituximab, is efficacious in ASyS (1, 2). In the case of Jo1 ASyS, identified by the most commonly detected anti-histidyl tRNA synthetase autoantibody, decreased autoantibody titers correlated with decreased clinical response and disease activity (3). More recently, anti-CD19 CAR-T therapy has been effective in inducing a drug free remission in a Jo1 ASyS patient (4). These findings support a pathogenic role for B cells in ASyS.

While rituximab, and potentially anti-CD19 CAR-T therapy, are efficacious in ASyS, they both indiscriminately kill B cells – irrespective of whether they recognize self vs. protective antigens. Broadly targeting B cells inhibits the ability of an individual to mount an effective vaccine response (5–7) and increases the risk of severe infection (8–10). Ideally, therapies would exist to target aberrant B cell pathways in autoimmune-prone B cells while sparing protective B cell responses.

Key circulating B cell subsets differ in ASyS patients relative to healthy controls, including a reduction in memory B cells amongst IgM^-^CD27^+^, IgM^+^CD27^+^, IgD^-^CD27^-^ (DN) populations, increased B_ND_ B cells (anergic, IgD^+^IgM^lo^), and increased naïve B cells (11–15). Single-cell technology has been used to profile the transcriptome of total peripheral blood mononuclear cells (PBMCs) in ASyS patients, but this study offered limited resolution within the B cell compartment, as B cells are typically a minority (10-20%) of PBMCs (16). We therefore used single-cell RNA sequencing of purified CD19^+^ cells to identify transcriptional changes within specific B cell populations in ASyS patients relative to healthy controls.

## 2 Materials and methods

All participants were enrolled into the Myositis and Scleroderma Treatment Initiative Center (MYSTIC) Cohort at Vanderbilt University Medical Center between 9/2017-10/2019. Informed consent was provided at the time of enrollment (VUMC IRB 141415). MYSTIC patients were recruited from both the inpatient and outpatient rheumatology and pulmonology services. Inclusion criteria were age 18 years or older at cohort enrollment, ASyS diagnosis by a rheumatologist or pulmonologist, and active disease at the time of enrollment (e.g. their treating provider thought increased immunosuppression was required for ongoing disease activity). Individuals were excluded if they had received cyclophosphamide or rituximab in the past five years. Clinical phenotyping of ASyS patients was performed via chart abstraction to determine serologic profile, disease manifestations, and current treatments. Healthy controls completed a health survey at the time of enrollment and did not report any history or signs/symptoms of an underlying rheumatic disease.

### 2.1 PBMC isolation

Peripheral blood was collected into mononuclear cell preparation tubes with sodium heparin (BD) via peripheral venipuncture. Peripheral blood mononuclear cells (PBMCs) were isolated, red blood cells were lysed with Ack Lysis Buffer (Gibco), and cells were washed 3X with 1X PBS. Cells were cryopreserved in 10% Dimethyl sulfoxide (DMSO) in fetal bovine serum (FBS) as previously described (15) and stored in liquid nitrogen until the time of experimentation.

### 2.2 B cell purification and single-cell sequencing

Cryopreserved PBMCs were rapidly thawed, washed, and resuspended in RPMI with 10% FBS. Cells were stained for flow cytometry sorting using the following reactive antibodies and reagents: CD3-e450 (OKT3), CD14-PE-Dazzle (M5E2), CD19-PE (SJ25C1), and LD700 viability dye (Invitrogen). Cells were also stained with cell hashing antibodies (**Supplementary Table 1**). Live CD3^-^CD14^-^CD19^+^ cells were flow cytometry purified using a BD FACSAria III, pelleted and resuspended in 1X PBS, and delivered promptly to the Vanderbilt Technologies for Advanced Genomics (VANTAGE) Shared Resource. VANTAGE staff prepared libraries and performed sequence amplification using the Chromium Next-GEM Single Cell 5’ Library & Gel Bead Kit, Chromium i7 Multiplex Kit, Chromium Single Cell A Chip kit, Chromium Single Cell V(D)J Enrichment (Human B Cell) Kit, and Chromium Single Cell 3’/5’ Library Construction Kit (10X Genomics). Five thousand CD19+ cells were targeted per participant sample. The VANTAGE core targeted 50,000 reads per cell which were sequenced using an Illumina NovaSeq6000 (S4) PE150. Data were de-multiplexed and processed using the CellRanger pipeline (v5.0.0, 10X Genomics) using GRCh38 as reference.

### 2.3 Single-cell clustering and differentially expressed gene analysis

Single-cell analysis was performed using Seurat v5.0.3 and SeuratObject v5.0.1 (17). Data were demultiplexed based on hashtag antibodies using the HTODemux function. Cells were removed that had fewer than 200 RNA features and/or > 10% mitochondrial genes. Doublets were called using the Seurat HTODmux function and were filtered out of the dataset. RNA-seq data were normalized and scaled using the SCTransform function. With regard to assigning B cell cluster and subset identity, we viewed immunoglobulin V gene usage as an uninteresting source of biological variation. Immunoglobulin (IGHV, IGKV, IGLV) genes were therefore removed from the RNA-seq gene list used as integration features prior to running FindIntegrationAnchors and IntegrateData to prevent them from driving transcriptionally defined clusters; integrated data were used for downstream clustering. BCR isotypes were determined using IgH heavy chain gene expression (e.g., IGHM, IGHG1, etc.) in the RNA-seq data and assigned in the meta.data slot in the Seurat object. B cell subset identities were assigned to clusters based on transcriptional profiles that were consistent with other studies defining these populations (18–20). These subsets (e.g., memory) were encoded in the Seurat object meta.data slot for use in downstream analyses. This study was not powered to address sex as a biological variable. Y-chromosome genes were therefore removed prior to differential gene expression and pathway analysis to prevent these genes from driving results, as otherwise any differences in the number of recovered male vs. female cells in a given cluster or subset could bias gene expression results. The Seurat FindMarkers function, which uses a non-parametric Wilcoxon rank sum test, was used to identify differentially expressed genes between ASyS and healthy control B cells within each transcriptionally defined cluster or for a particular gene of interest. Interactions between select differentially expressed genes were further visualized in Cytoscape version 3.10.1 using the default setting of a confidence score cut-off of 0.4 (21). All participants were included in cluster assignment, and subset level differentially expressed gene analysis. For additional analyses probing cells expressing high levels of a particular gene (e.g. FKBP5, MYADM, STAG3) within the activated or memory subsets, we classified cells as expressing a gene of interest if the level of expression was greater than 0.5 in the Seurat object data slot, which contains normalized expression values of each gene. We required that each included participant have at least 10 cells identified for activated/memory subset to be included in this deeper analysis.

### 2.4 Pathways analysis

Pathway enrichment was performed using g:Profiler (22) as previously described at https://baderlab.github.io/CBW_Pathways_2021/CANgprofiler-lab.html. The custom gene list (gmt) gprofiler_full_hsapiens.name.gmt was downloaded (http://baderlab.org/GeneSets) and utilized for this analysis. Analysis was preformed separately for the memory and activated subsets and the gene lists included genes with a fold change > 1.25 and p_adj_ < 0.01. Immunoglobulin genes were stripped from differential gene expression and pathway analysis. The analysis was run as a non-ordered query using the Benjamini-Hochberg FDR significance threshold set to 0.05 and included the data sources GO biologic process without electronic GO annotations, Reactome, and Wikipathways. Gene sets containing 10–500 terms were included for visualization in Cytoscape version 3.10.1 using the EnrichmentMap (settings p_adj_ < 0.01, Jaccard Overlap Combined = 0.375 edge similarity) and AutoAnnotate plugins.

### 2.5 Mitochondrial staining

Cryopreserved PBMCs from six healthy participants (including four who had undergone transcriptional sequencing) and twelve ASyS participants (including nine who had undergone transcriptional sequencing) were thawed as noted above. Staining with CM-H2DCFDA (e.g., DCFDA), TMRE, MitoTracker Green, and MitoSox (Invitrogen, USA) was performed per the manufacturer’s directions. Cells were concurrently stained with the following antibodies and reagents Live/Dead Aqua (Invitrogen), CD27-BUV737 (O323), BD19-BUV395 (SJ25C1), CD16-BV510 (M5E2), CD20-BV785 (2H7), IgM-Pacific Blue (MHM-88), and Qdot655-CD3 (S4.1). Data were acquired using a BD Fortessa 5-laser device and analyzed using CytoBank. Sample gating is shown in **Supplementary Figure 1**. The mean fluorescent intensity (MFI) of the PE (trimethylrhodamine ethyl ester (TMRE) or MitoSOX) and FITC (MitoTracker Green or 2’-7’-dichlorofluorescin diacetate (DCFDA)) was calculated for the CD27^-^IgM^+^ naive, CD27^+^IgM^+^ non-class switched memory, and CD27^+^IgM^-^ class switched memory subsets. To normalize by mitochondrial mass, a correction factor was determined by dividing the MitoTracker green MFI of a given sample and subset by the average MitoTracker green MFI for the six healthy participant samples for that particular subset. The raw MFI for TMRE, MitoSOX, and DCFDA for a given sample subset was then multiplied by the correction factor to yield the normalized MFI.

### 2.6 Statistical analysis

All statistical analysis was performed using GraphPad Prism software version 8.3.1 (GraphPad Software) or the stats package v4.3.2 in R. The individual statistical tests used are specified in figure legends.

## 3 Results

### 3.1 The frequency of transcriptionally defined memory B cells is decreased in ASyS patients relative to healthy controls

We previously found that phenotypically defined aberrant B cell populations are increased in patients with IIM (23). Alterations in various B cell subsets, including memory populations, are also reported within ASyS patients (12–15). We therefore sought to determine whether gene expression changes were present in ASyS patient B cells, relative to healthy controls. To address this question, single-cell RNA-sequencing technology was used as in Methods to profile CD19+ cells isolated from ASyS and age and sex-matched healthy donors. Baseline participant characteristics are shown in **Table 1**; detailed clinical phenotyping of the 10 ASyS participants is shown in **Supplementary Table 2**. All ASyS patients required increased immunosuppression at the time of enrollment. Seven patients were receiving glucocorticoids at the time of enrollment. Two patients were receiving either mycophenolate or azathioprine at enrollment. Notably, ASyS4 presented simultaneously with non-Hodgkin’s lymphoma and ASyS 7.5 years prior to admission and was successfully treated with rituximab, cyclophosphamide, doxyrubicin, vincristine and prednisone (R-CHOP) with drug-free remission of non-hodgkins lymphoma and ASyS until cohort enrollment.

**Table 1:** Baseline characteristics of included subjects^‡^.

Characteristic	ASyS patients (n=10)	HC (n=5)*
Age at enrollment (yrs)^†^	55 (41.5,58)	53 (43,59)
Female sex	5/10	3/5
BIPOC	5/10	2/5
Disease duration (yrs)^†^	1.2 (0.9,3.8)	
Anti-synthetase antibody		
Jo1	5/10	
PL7	1/10	
PL12	4/10	
Muscle involvement		
Proximal weakness	4/10	
Elevated CK/aldolase/LDH	7/10	
Skin involvement		
Mechanic’s hands	6/10	
Heliotrope rash	3/10	
Gottron’s sign/papules	2/10	
Inflammatory arthritis	7/10	
Interstitial lung disease	10/10	
Therapies at enrollment		
Prednisone	7/10	
Mycophenolate	1/10	
Azathioprine	1/10	

ASyS, antisynthetase syndrome; BIPOC, Black, Indigenous, and Persons of Color; HC, healthy control.

^†^Expressed as median(interquartile range).

^‡^ Subject level data is shown in **Supplementary Table 2**.

**Figure 1:**
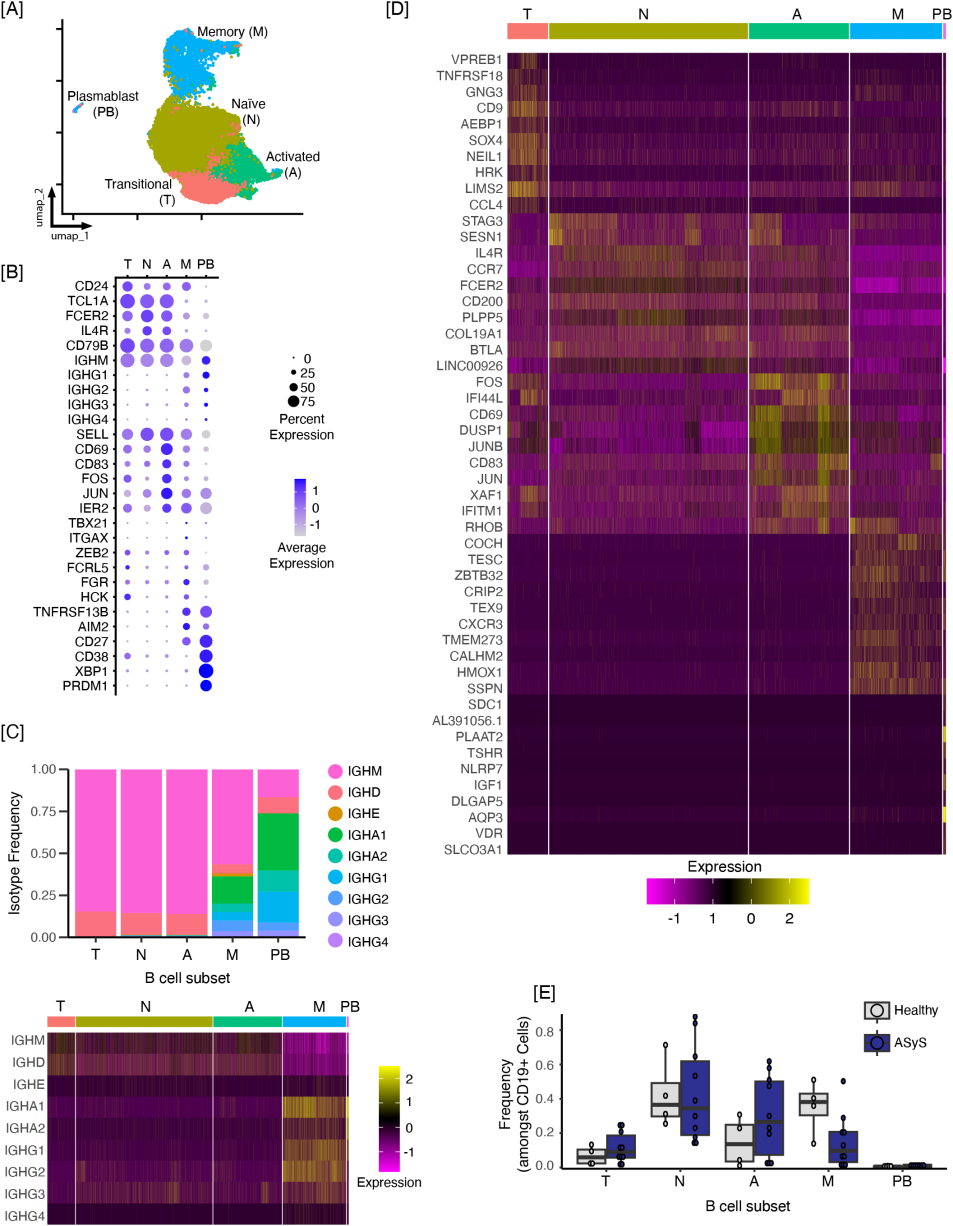
Anti-synthetase syndrome patients show skewed B cell subset distribution from healthy controls. Single-cell RNA-seq technology was used to target 2000–5000 purified CD19^+^ CD3^-^ cells isolated per donor from n = 10 antisynthetase (ASyS) patients and n = 5 healthy controls (HC). Seurat was used to identify 12 clusters which were further grouped as naïve, transitional, activated, memory, and plasmablast subsets based on manual inspection of isotype and gene expression profiles for each cluster. **(A)** UMAP showing RNA-seq-based B cell subsets. **(B)** Manually selected gene expression profiles are shown for each B cell subset. **(C)** Isotype distribution based on immunoglobulin heavy chain genes is shown by subset in the bar chart (top) and as a heatmap by subset (bottom), **(D)** Top ten significantly upregulated genes by B cell subset (p adj. < 0.05). **(E)** Boxplots show the mean frequency of each B cell subset by disease group, with individual donors plotted as points. HC2 was excluded due to only isolating 36 cells from this individual. Formal nonparametric statistical comparisons were not feasible due to only including 4 HC samples. Subset abbreviations: A=activated, M=memory, N=naïve, PB=plasmablast, and T=transitional.

First, we sought to identify various B cell subsets to probe transcriptional differences across the B cell compartment. Low-quality cells were filtered out based on the number of expressed genes and mitochondrial gene content, as described in Methods. Data were additionally filtered to remove doublets. A total of 18,018 ASyS B cells and 4,788 healthy participant CD19+ cells were included in downstream analysis. The median number of cells isolated in ASys was comparable to healthy controls (1293 [IQR 607, 1839] v. 867 [IQR 322, 1639], p=0.37). Seurat identified 12 unique clusters (**Supplementary Figures 2**, **S3**), which were manually merged and assigned into the following five major B cell subsets: transitional, naïve, activated, memory, and plasmablasts (**Figure 1A**). Participant level data for B cell subset frequency is shown in **Supplementary Table 3**. HC2 had a low number of recovered B cells, and as such, did not contribute significantly to cell identity assignments or differential gene expression. These subset identities were assigned based on isotype skewing (**Figure 1B**) and expression of selected genes known to correspond with these subsets (**Figure 1****C** and (18–20)). For example, transitional B cells expressed increased levels of CD24, CD38, and TCL1A relative to naïve B cells. The activated population expressed increased levels of the activation markers, CD69 and CD83. Both memory and plasmablast subsets showed increased expression of CD27, and the plasmablast population expressed genes associated with antibody-secreting cell fate, XBP1 and PRDM1. The top ten differentially expressed genes for each subset are shown in **Figure 1D**. While we acknowledge the complexity within each of these broader subset categories (e.g., many phenotypically and functionally distinct subtypes of memory B cells have been identified, reviewed in (24)), this higher-level categorization provides a convenient framework within which we can identify gene expression changes that are most broadly present in ASyS patients (relative to healthy controls), in balance with avoiding gene expression changes that merely track with a decreased abundance of a given B cell subset (e.g., memory), as a means to identify those aberrant genes and pathways that may be the most enticing targets for therapeutic modulation to limit autoimmunity, and consequently tissue damage.

The frequency of B cells within each subset for individual donors is shown in **Figure 1E**. As HC2 had a low number of recovered B cells compared to other participants, subset frequencies for HC2 were excluded from **Figure 1E**. These data appear to recapitulate prior flow cytometry phenotyping-based findings showing that ASyS patients have reduced frequencies of memory B cells relative to healthy controls (12–15), although removal of HC2 precludes statistical analysis. Memory cluster 3, which expresses a classical memory signature, appears to be the most dramatically reduced (**Supplementary Figure 2**). Intriguingly, BCR isotype, which distinguishes IgM memory vs. class-switched memory, did not drive the different memory clusters observed. Rather, they were driven by genes associated with classical vs. atypical memory, which represent different transcriptional (and presumably functional) states which was dominant over isotype (also encoded in the list of genes used for clustering and gene expression analysis). DN2 are another subset of memory B cells that are increased in SLE and share features with atypical B (25). We observed that memory cluster 8 appeared to have an atypical memory/DN2 B cell signature (including upregulation of ITGAX (CD11c) and FCRL5). However, given that DN2 B cells have largely been defined in the literature based on expression of phenotypic markers, we are avoiding definitively naming that cluster as DN2, as it may encompass other cell types beyond the DN2 population. Of note, this was a low abundance cluster (consistent with the literature) that was present at similar frequencies in both healthy controls and ASyS patients.

### 3.2 Activated B cells in ASyS patients express either an interferon signature or cell stress related genes

We performed differential gene expression and pathway analyses within each B cell subset to reduce potential bias driven by the decreased memory B cell frequency observed in the ASyS patient group relative to healthy controls (**Figure 1E**). Using the cut-offs of absolute fold change > 1.25 and p_adj_ < 0.01, transitional cells differentially expressed 44 genes (**Supplementary Table 4**), naïve B cells differentially expressed 122 genes (**Supplementary Table 5**), activated B cells differentially expressed 84 genes (**Supplementary Table 6**), and memory B cells differentially expressed 396 genes (**Supplementary Table 7**). Due to the limited number of plasmablast cells identified, only one gene (U2AF1L5, absolute fold change = 28.7, p_adj_ = 0.005) was differentially expressed using these cut-offs. Given the key role for activated and memory B cells in immune responses, we focused on these two subsets for further analysis.

**Figure 2:**
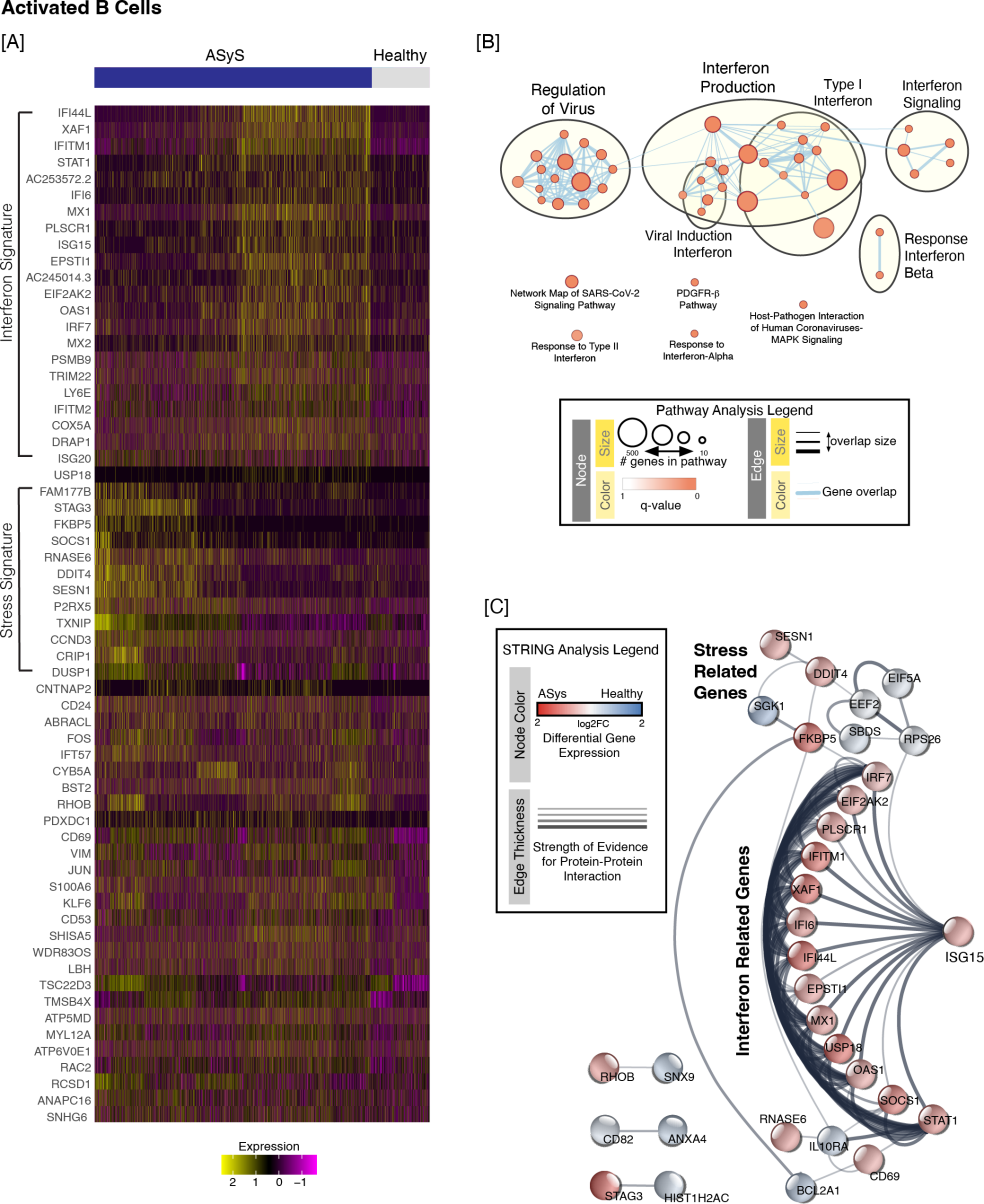
An enriched interferon signature was observed in activated ASyS B cells compared to healthy donor B cells. Activated B cells were identified as in **Figure 1**. **(A)** n = 62 upregulated genes were identified in ASyS compared to healthy participants based on the following cutoffs: fold change > 1.25 and adjusted p value < 0.01, which are plotted on a heatmap for ASyS (blue bar) or healthy (grey bar) donors. **(B)** Pathway analysis using g:Profiler (as outlined in Methods) identified 47 pathways that were overrepresented (FDR significance threshold set to 0.05) amongst upregulated genes in activated ASyS B cells compared to healthy participants. **(C)** STRING analysis was then performed as in Methods and used to identify putative protein-protein interactions between the top 25 up- and down-regulated genes in ASyS compared to healthy participants for the activated B cell subset.

To gain preliminary insights into how activated B cells in ASyS patients differed from healthy controls, we performed pathway analysis of the differentially expressed genes with an absolute fold change > 1.25 and p_adj_ < 0.01 using g:Profiler, which identified 62 upregulated (**Figure 2A**) and 22 downregulated genes (not shown). Examination of upregulated genes revealed that a subset of activated ASyS B cells had a more interferon-related signature while others appeared to upregulate genes related to cellular stress (**Figure 2A**). Forty-seven upregulated pathways were overrepresented in ASyS B cells with p_adj_ < 0.01 (**Supplementary Table 8**). Overrepresented pathways were further visualized using EnrichmentMap (**Figure 2B**). Detailed expression data for highlighted gene ontology terms are included in **Supplementary Table 9**. Notably, interferon related pathways dominated the enrichment analysis, in line with other reports that noted an increased interferon signature in total B cells (16) and memory B cells (26). Even for the seemingly unrelated platelet derived growth factor receptor beta (PDGFR-B) pathway, the upregulated genes included the interferon-related genes STAT1 and EIF2AK2 as well as FOS and JUN.

We next utilized STRING analysis, as in Methods, which visually organizes gene expression data based on the strength of experimental evidence supporting direct protein-protein interactions for each gene. STRING networks for the top 25 upregulated and 25 downregulated genes in ASyS B cells are shown in **Figure 2C** and **Supplementary Table 10**. Similar to pathway analysis, STRING analysis revealed a group of 14 interferon-related genes (**Figure 2C****),** further highlighting the strength of the interferon-related signature in activated B cells of ASyS patients. In addition to this, there was a group of cellular stress related proteins – namely SESN1, DDIT4, SGK1, and FKBP5 – that were upregulated in activated B cells in ASyS vs. healthy donors (**Figure 2C**).

### 3.3 Memory B cells in ASyS patients upregulate interferon, actin, and chemical stress pathways

**Figure 3:**
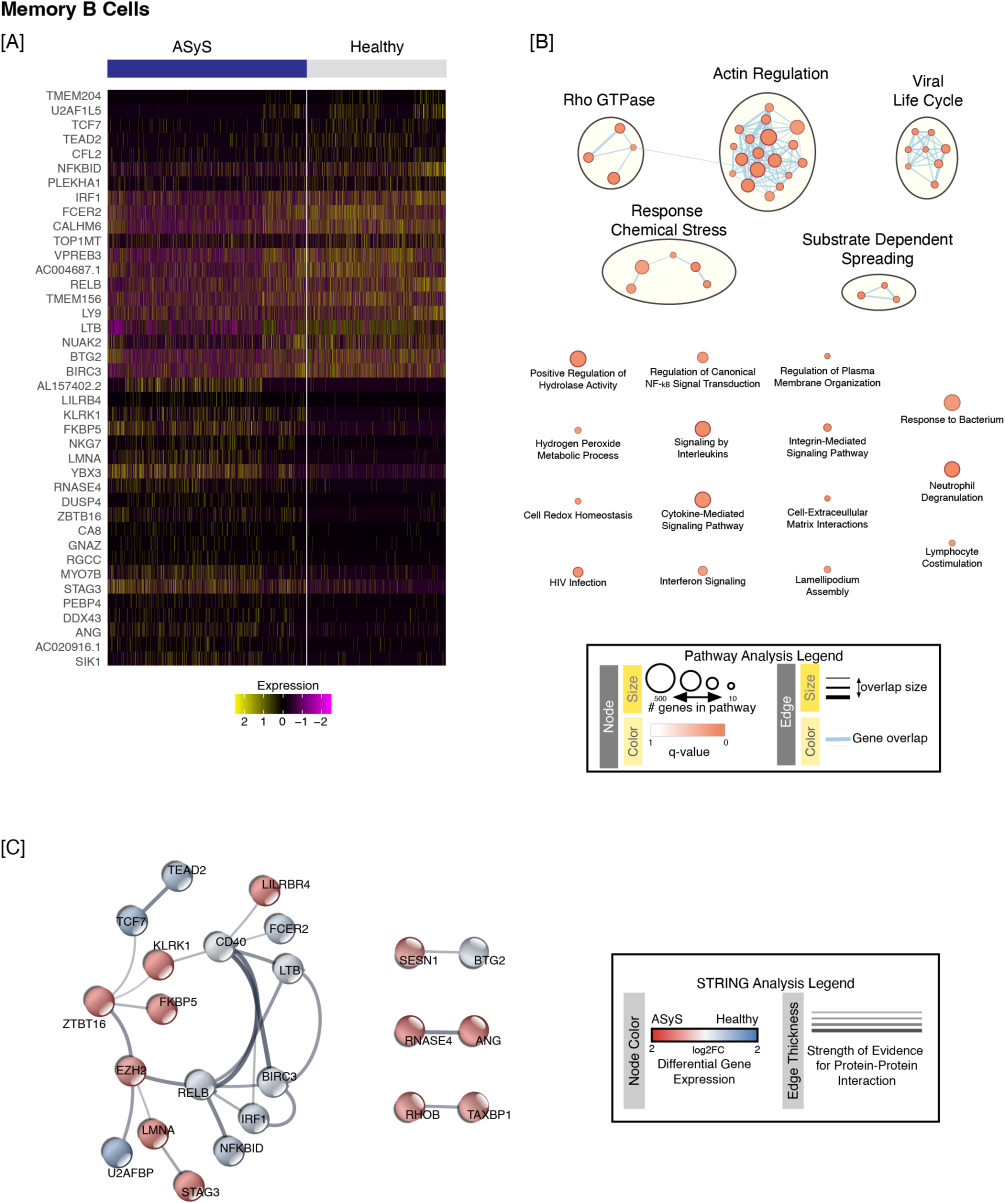
Transcriptional differences were observed in memory B cells isolated from ASyS compared to healthy donors. Memory B cells were identified as in **Figure 1**. **(A)** Heatmap showing the top 20 downregulated genes and top 20 upregulated genes in ASyS (blue bar) compared to healthy participants (grey bar) based on the following cutoffs: fold change > 1.25-and adjusted p value < 0.01. **(B)** Pathway analysis using g:Profiler (as in Methods) identified 53 pathways that were overrepresented (FDR significance threshold set to 0.05) amongst upregulated genes in memory B cells isolated from ASyS compared to healthy participants. **(C)** STRING analysis was then performed as in Methods and used to identify putative protein-protein interactions between the top 25 up- and down-regulated genes in ASyS compared to healthy participants for the memory subset.

We next performed pathway analysis of the memory subset using the same cut-offs of an absolute fold change of 1.25 and p_adj_ < 0.01. A total of 360 upregulated and 36 downregulated genes were identified by our analysis (**Supplementary Table 7**). Fifty-three upregulated pathways were overrepresented with p_adj_ < 0.01 (**Supplementary Table 10**). A heatmap of the top 20 downregulated and top 20 upregulated genes in memory ASyS B cells is shown in **Figure 3A**. These pathways were further visualized using EnrichmentMap (**Figure 3B**) and detailed expression data for highlighted gene ontology terms is included in **Supplementary Table 12**. STRING analysis of the top 25 up- and downregulated genes (**Supplementary Table 13**) in memory ASyS B cells is shown in **Figure 3C**. Three common themes were present in overrepresented pathways, namely cellular cytoskeleton rearrangement, the interferon/viral related pathways and response to chemical stresses. Notably, cellular stress related proteins SESN1, DDIT4, and FKBP5 were upregulated in both activated and memory B cells (**Supplementary Tables 6**, **S7**).

**Figure 4:**
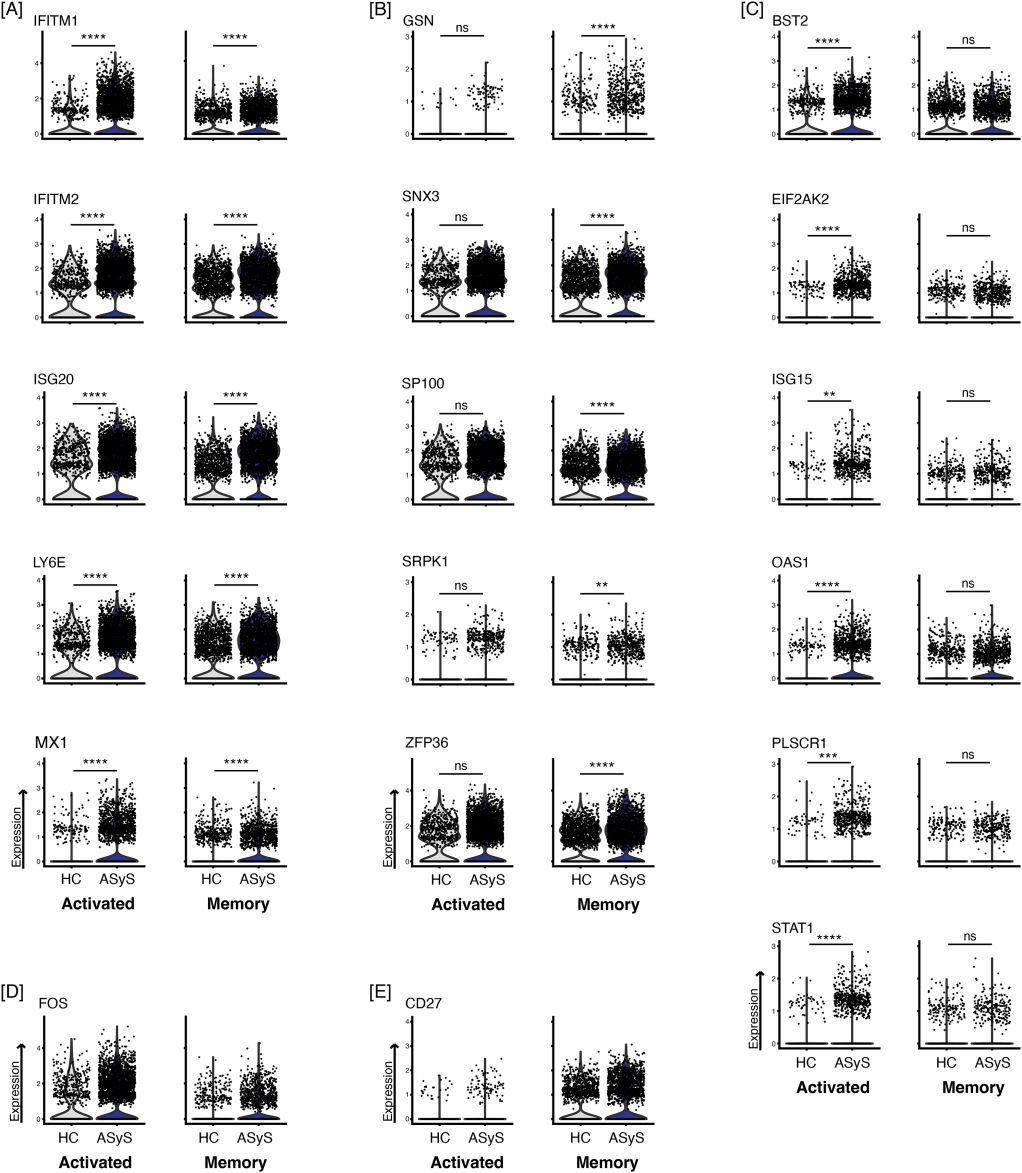
Genes in the Negative Regulation of Viral Process Pathway (GO:0048525) are increased in the activated and memory subset in ASyS compared to healthy controls. Several genes in GO:0048525 are upregulated in **(A)** both the activated and memory subset, **(B)** the memory subset, or **(C)** the activated subset in ASyS compared to healthy controls. Between group comparisons were made with a Wilcoxon-Rank Sum test and significance is denoted as n.s. = not significant, **padj<0.01, ***padj<0.001, ****padj<0.0001. As shown in **Figures 1C**, **(D)** FOS is increased in the activated subset (relative to the memory subset) and **(E)** CD27 is increased in the memory subset (relative to the activated subset).

Both activated and memory B cell subsets upregulated interferon response genes, IFITM1, IFITM2, ISG20, LY6E, and MX1 (**Figure 4**). Negative regulation of viral process term (GO:0048525) was the most overrepresented shared gene ontology term for activated (p_adj_ = 2.77E-12) and memory B cells (p_adj_ = 1.23E-03). However, the upregulated genes in this gene ontology term differed in activated and memory B cell subsets of ASyS. In addition, altered expression of some interferon and viral regulation pathways varied between the activated and memory B cell subsets.

Actin filament organization (GO:0007015) was the most enriched pathway; 33/360 upregulated memory genes were identified in this pathway (p_adj_=1.0E-8). Actin cytoskeleton facilitates BCR organization, signaling, and internalization (reviewed in (27)) The top 5 differentially upregulated genes in this pathway were RGCC (FC 5.7, p_adj_ = 0.0004), MYO7B (FC 4.5, p_adj_ = 2.4E-16), ANG (FC 4.0, p_adj_ = 1.1E-8), RHOB (FC 3.3, p_adj_ = 7E-62), and PTGER4 (FC 2.3, p_adj_ = 0.0002). Angiogenin (ANG) has been implicated in cell migration (28) and RHOB belongs to the Rho family of small GTPases that impacts cytoskeleton rearrangement (reviewed in (29)) which has been implicated in autoantibody production in K/BxN and MRL/*lpr* mouse models (30). PTGER4 regulates B cell proliferation and differentiation (31) and RGCC regulates cell cycle progression and is induced by complement activation (32). Of the 33 upregulated genes in this pathway in memory B cells, only three (RHOB, TMSB4X, and RAC2) were also upregulated in activated ASyS B cells. Of note, RAC2 controls BCR synapse formation and ICAM-1 adhesion; B cells over-expressing Rac2 in mice were highly adhesive (33), which could influence the quality of T cell help received.

The Reactome term cellular response to chemical stress (REAC:R-HAS-9711123) was the most overrepresented chemical stress pathway; 19/360 upregulated genes were represented in this pathway (FDR 2.7E-5). The top five upregulated genes in this term were SESN1 (FC 3.1, p_adj_ = 7.6E-10), HMOX1 (FC 2.0, p_adj_ = 1.2E-14), KEAP1 (FC 1.6, p_adj_ = 0.003), TXNIP (FC 1.6, p_adj_ = 7.7E-23), PRDX6 (FC 1.5, p_adj_ = 6.0E-5). SESN1 is a key sensor that promotes cell survival under stress conditions induced by cytokines, chemokines, and reactive oxygen species (34). TXNIP regulates germinal center formation and proliferation (35, 36). Reactive oxygen species oxidize KEAP1, which leads to induction of HMOX1 transcription, and ultimately affects B cell activation and differentiation (37). While most ROS are generated by the mitochondria, the NADPH oxidase complex (NOX2) is another important source of intracellular ROS. Notably, the p67phox subunit encoded by NCF2 was also increased in memory ASyS B cells (FC 1.5, p_adj_ =7.5E-03).

### 3.4 Reactive oxygen species are increased in B cells from ASyS patients, relative to healthy controls

**Figure 5:**
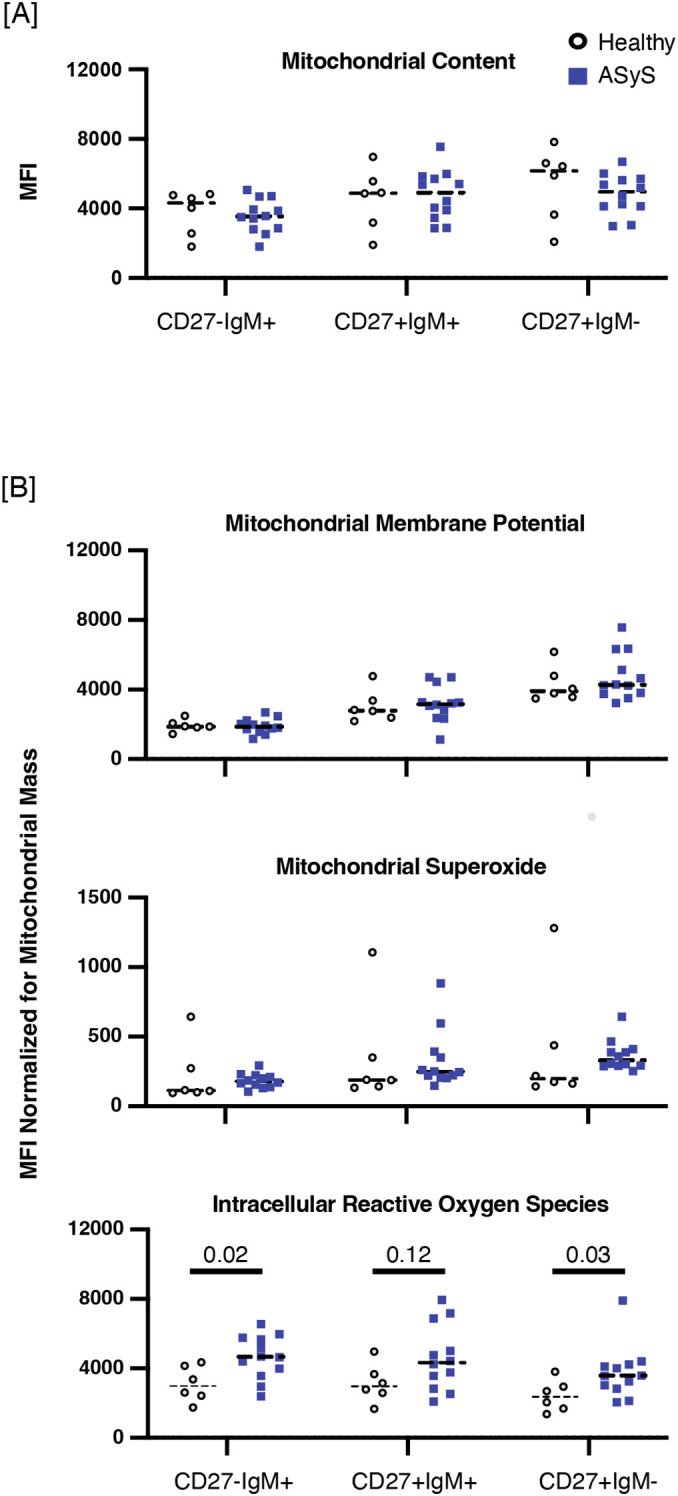
Intracellular reactive oxygen species are increased in ASyS compared to healthy B cells. CD27-IgM+, CD27+IgM+, and CD27+IgM- B cell populations were compared as follows between ASyS (blue squares) and healthy participants (open circles). **(A)** Mitochondrial content was assessed using MitoTracker green. **(B)** Data were corrected for mitochondrial content and mitochondrial membrane potential was measured by TMRE (top panel), mitochondrial superoxide was measured by MitoSox (middle panel), and intracellular reactive oxygen species were measured by DCFDA (bottom panel). Statistical comparisons were made using Mann-Whitney U test.

These transcriptional findings raise a key question as to whether reactive oxygen species are increased in ASyS B cells. We therefore experimentally quantified the mitochondrial bulk using MitoTracker Green, mitochondrial membrane potential using TMRE, intracellular reactive oxygen species using DCFDA, and mitochondrial superoxide using MitoSOX in specific B cell subsets (raw data provided in **Supplementary Table 14**). There was no difference in the mitochondrial content in memory or non-memory ASyS B cells compared to healthy controls (**Figure 5A**), but there was significant variability in mitochondrial content amongst donors. We therefore normalized the mean fluorescent intensity (MFI) of TMRE, DCFDA, and Mitosox for mitochondrial bulk (**Figure 5B**). There was no difference in the mitochondrial membrane potential, or superoxide in CD27^-^IgM^+^, CD27^+^IgM^+^, or CD27^+^IgM^-^ B cells of ASyS compared to healthy participants. In contrast, ASyS participants had increased intracellular reactive oxygen species, as measured by DCFDA, compared to healthy participants in both the CD27^-^IgM^+^ B subset (MFI 4673.1 [IQR 3670.3, 5744.7] vs. 2983.5 [IQR 2268.0, 4205.1], p=0.03) and CD27^+^IgM^-^ B cells (MFI 3580.6 [IQR 2880.7,4193.8] vs. 2367.5 [IQR 1614.4, 3162.5], p=0.02). There was a trend towards increased intracellular reactive oxygen species in CD27^+^IgM^+^ B cells (MFI 4336.0 [IQR 3010.4, 6400.8] vs. 2977.3, [IQR2356.1, 3998.0], p=0.12).

### 3.5 The frequency of memory B cells expressing detectable FKBP5, MYADM, and STAG3 is increased in ASyS participants relative to healthy controls

A subset of ASyS B cells showed an increased interferon signature while other ASyS B cells showed increased expression of cell stress genes (**Figure 2A****).** This highlights the potential for heterogeneity in B cell gene expression programs across donors, even when comparisons are restricted to B cells belonging to similar subsets (e.g., activated). We therefore identified genes for which expression was increased in a higher proportion of activated or memory B cells in ASyS relative to healthy donors. Thus, we filtered the afore described significant gene lists using the following criteria: 1) gene expressed in < 10% of healthy cells and 2) percent of ASyS B cells expressing a specific gene increased by > 10% compared to healthy cells across all donors in aggregate. Three candidate genes (FAM177B, IFI44L, and STAG3) had increased frequency of expression in activated ASyS B cells; nine candidate genes (AL157402.2, EBI3, FKBP5, MPST, MYADM, STAG3, TAX1BP3, WEE1, YBX3) had increased frequency of expression in memory ASyS cells (**Supplementary Table 15**). We then calculated the frequency of each candidate gene in the relevant B cell compartments for all healthy and ASyS participants (**Supplementary Table 16**, **Supplementary Figure 4**). As noted in the Methods, we required that at least 10 cells were detected per donor for the memory and activated subset for frequency calculations of cells that did/did not express each gene. ASyS6 had eight activated B cells, and ASyS 10 had only one memory B cell. All HC participants, including HC2, had at least 10 activated and memory B cells. There was no statistically significant difference in the frequency of any candidate gene expression in activated B cells for ASyS compared to healthy participants for this individual donor-level analysis. Three genes, FKBP5 (11.1% [IQR 2.9%, 22.2%] vs. 1.0% [IQR 0.4%, 3.4%], p=0.04), MYADM (17.0% [IQR 12.8%, 20.2%] VS. 4.9% [IQR 1.4%, 10.6%], p=0.01), and STAG3 (15.8% [IQR 8.8%, 31.3%] vs. 6.1% [IQR 0.5%, 9.4%], p=0.04) were more frequently expressed in memory B cells from ASyS compared to healthy individuals.

### 3.6 FKBP5 expressing memory B cells are increased in a subset of ASyS participants and show a cellular stress phenotype.

**Figure 6:**
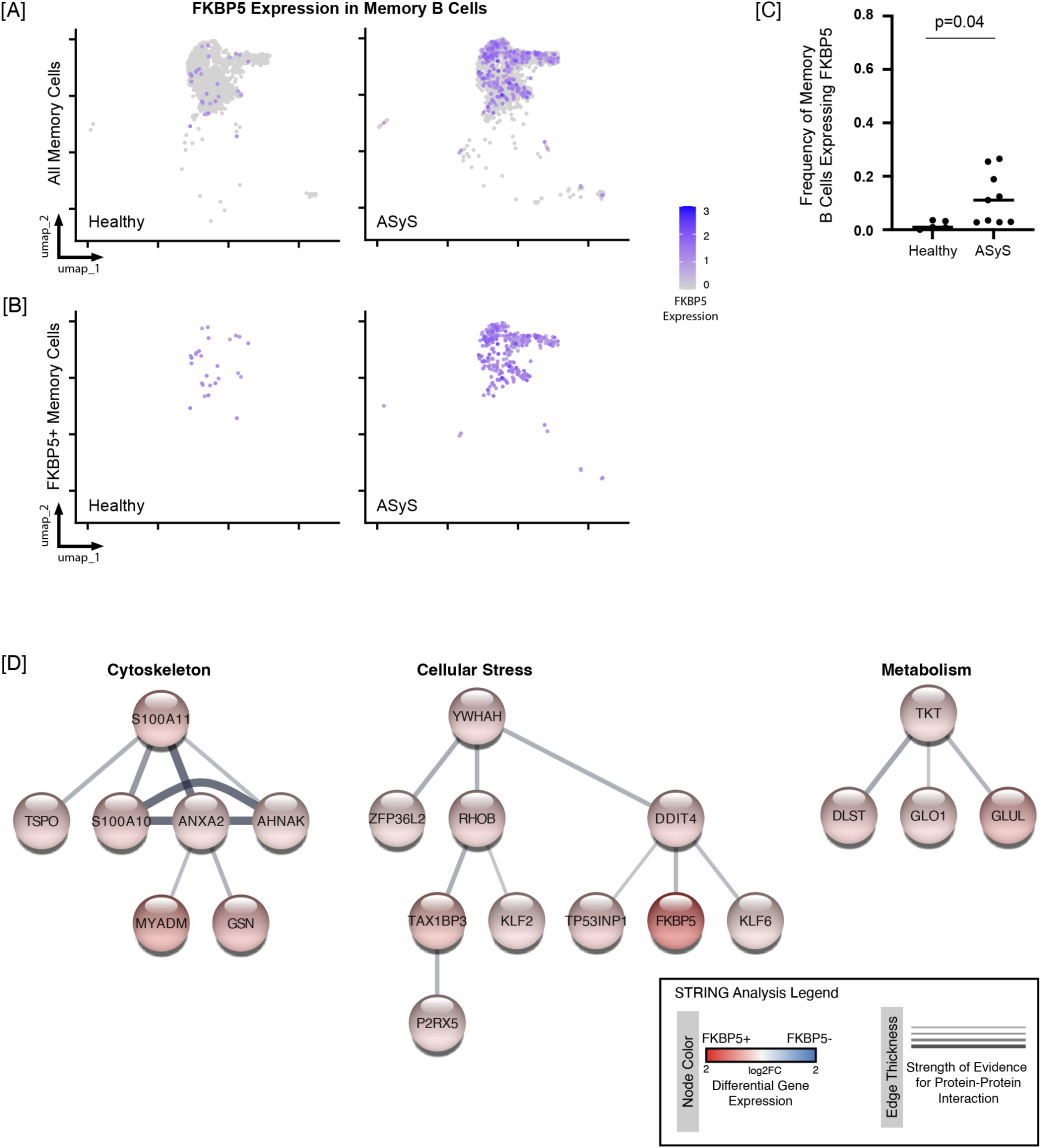
The frequency of FKBP5-expressing memory B cells is increased in ASyS patients compared to healthy controls and is associated with upregulation of key B cell response pathways. Memory B cells were identified as in **Figure 1**. **(A)** UMAPs show FKBP5 gene expression across all healthy (left) or ASyS (right) memory B cells or **(B)** only FKBP5-expressing cells. Memory B cells were categorized as FKBP5-expressing cells if the level of expression was greater than 0.5 in the Seurat object data slot, which contains normalized expression values of each gene. **(C)** The frequency of FKBP5+ cells (within memory B cells) is plotted for individual healthy control or ASyS participants, p = 0.04, Mann Whitney-U test. **(C)** STRING analysis identified three clusters of upregulated genes (fold change > 1.25 and adjusted p value < 0.01) with putative protein-protein interactions shown by connecting lines, with thickness showing strength of evidence for interaction.

We next wanted to identify differentially expressed genes present in B cells expressing FKBP5, MYADM, or STAG3. FK506 binding protein 5 (FKBP5) is one of the many targets of the calcineurin inhibitors tacrolimus and rapamycin, which are among the arsenal of drugs used to treat ASyS. While most immunosuppressive effects of tacrolimus are thought to be related to binding to FKBP12 (38), prior work has also indicated that tacrolimus may further act on FKPB5, which modulates lymphocyte co-activation via PD-L1 (39) and inhibits endogenous MHC class II antigen presentation (40). We dichotomized gene expression as positive or negative for FKBP5. Cells were called “positive” if the level of expression was greater than 0.5 in the Seurat object data slot, which contains normalized expression values for each gene. FKBP5 expression amongst all memory B cells (**Figure 6A**), and amongst only those cells scored as FKBP5+ (**Figure 6B**) is shown on UMAPs. Given that UMAPs are not an ideal way to represent population frequency, **Figure 6C** shows the proportion of FKBP5+ cells amongst total memory B cells in healthy or ASyS individuals. While the frequency of FKBP5 expressing memory B cells was increased across all ASyS participants compared to healthy controls (11.1% [IQR 2.9%,22.2%] vs. 1.0% [IQR 0.4%,3.4%], p=0.04), the increased frequency of FKBP5+ memory B cells was greatest seen in participants 1-5 (**Figure 6C**, **Supplementary Table 16**). Because lymphocytes are known to upregulate FKBP5 after glucocorticoid exposure (41), it is important to note that the three steroid naïve participants (participants 3-5) had FKBP5+ memory B cell frequencies of 12.4%, 25.5%, and 11.1%, respectively.

Pathway analysis using the cut-offs of an absolute fold change of 1.25 and p_adj_ < 0.01 identified 55 genes that were upregulated and one downregulated gene in FKBP5+ compared to FKBP5- memory B cells (**Supplementary Table 17**). The only two overrepresented pathways amongst the upregulated genes were regulation of plasma membrane organization (GO:1903729, 4/17 genes upregulated, p_adj_=9.4E-5) and membrane raft organization (GO:0031579, 4/25 genes upregulated, p_adj_=3.0E-4). These two GO terms shared the upregulated genes GSN (FC 2.0, p_adj_=4.5E-6), S100A10 (FC 1.7, p_adj_=2.0E-7), and ANXA2 (FC 1.5, p_adj_=1.7E-4). AHNAK (FC = 1.5, p_adj_=5.0E-4) was upregulated in regulation of plasma membrane organization pathway; MYADM (FC 2.5, p_adj_=7.1E-6) was upregulated in the membrane raft organization pathway.

We next employed STRING analysis (as in Methods) to visualize putative protein-protein interactions between the 56 upregulated genes in FKBP5+ memory B cells. Three groups of three or more interacting genes were identified (**Figure 6C**, **Supplementary Table 18**). The first group, including S100A11, contained the membrane organization genes identified in the earlier pathway analysis. The second group, led by YWHAH included several genes associated with cellular stress including DDIT4 (42), TP53INP1 (43), KLF6 (44), and FKBP5 (45, 46). The third group contained several key metabolic enzymes including TKT from the pentose phosphate pathway, DLST from the TCA cell cycle, GLO1 from the glyoxalase system, and the glutamine synthesis gene GLUL. These transcriptional changes indicate that FKBP5+ memory B cells have altered metabolism compared to other memory B cells.

Given the prior reports of efficacy of tacrolimus in ASyS (47, 48), we also investigated if other FK506 binding proteins had differential expression in healthy versus ASyS memory B cells (**Supplementary Table 19**). FKBP1A, which is considered to be the primary immunosuppressive target of tacrolimus and rapamycin, was increased in ASyS memory compared to healthy B cells (FC 1.21, padj=2.52E-04), although the difference in frequency of expression was not statistically significant (31.9% [IQR 21.1%, 50.5%] vs. 17.7% [IQR 14.1%, 36.9%]. p=0.11). FKBP3 was also increased in terms of both general upregulation (FC 1.46, padj = 1.50E-04) and frequency of expression (24.7% [IQR 20.6%, 32.2%] vs. 17.7% [IQR 14.1%, 22.1%], p=0.03). Additionally, the cell cycle regulator cyclin D3 (CCND3) was transcriptionally upregulated in ASyS memory B cells (FC 2.60, p_adj_ = 1.33E-102) and detectably expressed in an increased proportion of ASyS memory B cells (67.1% [IQR 55.6%, 79.4%] vs. 35.6% [IQR 29.4%, 40.6%], p=0.002), relative to healthy controls. CCND3 is a known target of rapamycin in both B and T cells (49). Together, these findings indicate that FKBPs and their downstream targets may be important regulators of memory B cells in ASyS.

### 3.7 MYADM expressing memory B cells upregulate pathways involved with cell adhesion, migration, and NF-KB signaling relative to healthy controls

**Figure 7:**
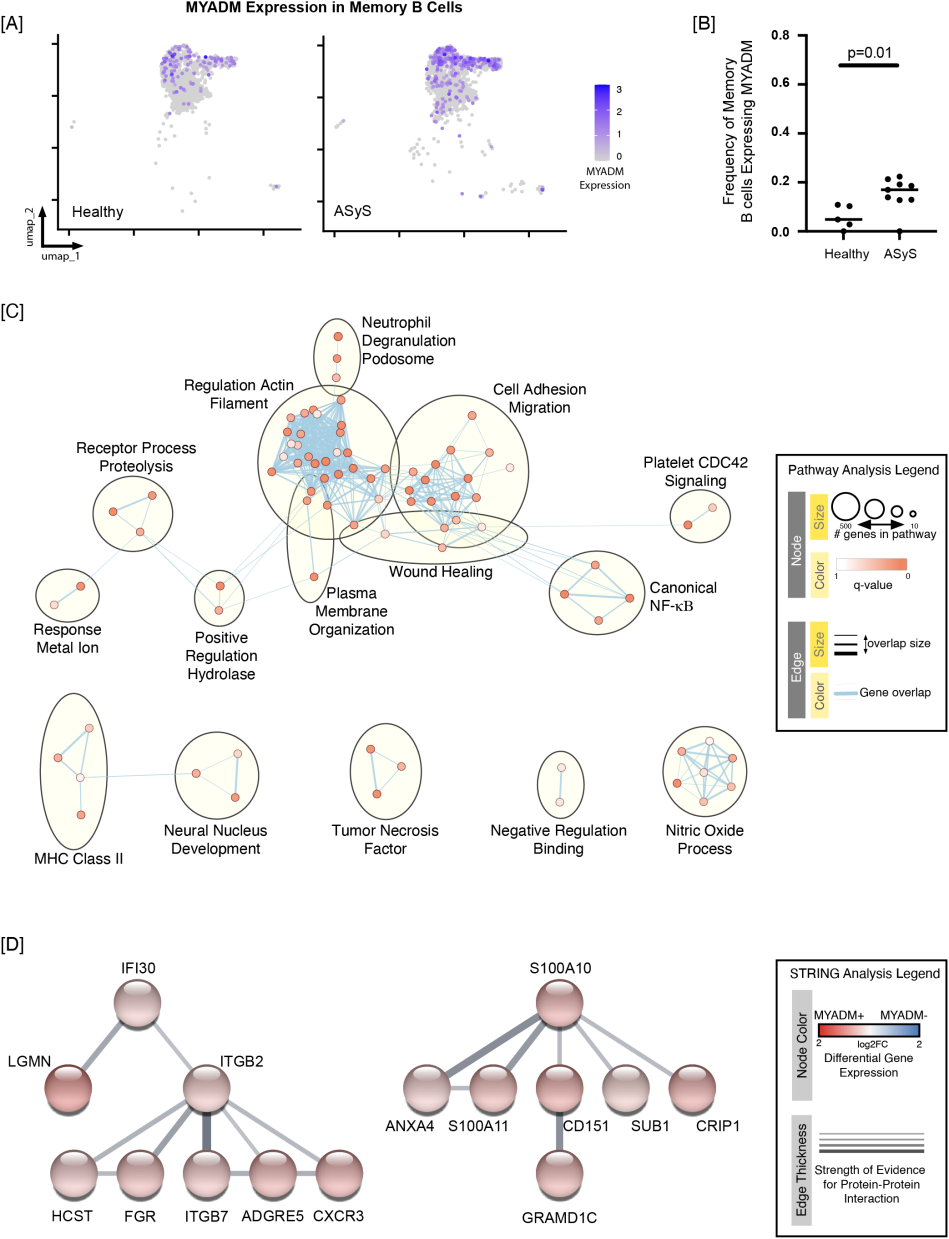
MYADM expression is increased in ASyS compared to healthy memory B cells and is associated with upregulation of multiple gene ontology pathways. **(A)** UMAPs show MYADM gene expression across all healthy (left) or ASyS (right) memory B cells (identified as in **Figure 1**). **(B)** Memory B cells were categorized as MYADM-expressing cells if the level of expression was greater than 0.5 in the Seurat object data slot. The frequency of MYADM+ cells (within memory B cells) is plotted for individual healthy control or ASyS participants, p < 0.01, Mann-Whitney U test. **(C)** Pathway analysis using g:Profiler identified 79 pathways that were overrepresented amongst upregulated genes (fold change > 1.25 and adjusted p value < 0.01) in MYADM+ compared to MYADM- memory B cells, which are visualized using EnrichmentMap and Cytoscape. **(D)** Two clusters of upregulated proteins with putative protein-protein interactions were identified using STRING analysis.

Myeloid-associated differentiation marker (MYADM) is a transmembrane protein that was initially identified to be upregulated during myeloid differentiation (50) but is also known to be expressed in multiple other tissues (51, 52). MYADM regulates membrane organization and lipid domains in the plasma membrane (53). To probe frequency changes of MYADM expressing-memory B cells amongst healthy and ASyS donors, we dichotomized gene expression as positive or negative based on the level of expression being greater than (positive) or less than (negative) 0.5 in the Seurat object data slot, which contains normalized expression values for each gene. MYADM expression amongst all memory B cells is shown on the UMAPs (**Figure 7A**), with only those cells scored as MYADM-positive plotted in **Supplementary Figure 5**. As discussed above, UMAPS are suboptimal for visualizing changes in population frequencies, thus the donor level frequency of MYADM expression in memory B cells is plotted in **Figure 7B**. MYADM was expressed in a much larger proportion of ASyS memory B cells compared to healthy controls (17.0% [IQR 12.8%, 20.2%] vs. 4.8% [IQR 1.4%, 10.6%], p=0.01); all but one ASyS participant had an increased frequency of MYADM+ memory B cells. We therefore sought to understand how MYADM+ memory B cells differed from MYADM- memory B cells. Differential expression gene analysis using the cut-offs of an absolute fold change of 1.25 and p_adj_ < 0.01 identified 188 upregulated and three downregulated genes in MYADM+ compared to MYADM- memory B cells (**Supplementary Table 20**). Pathway analysis revealed 79 overrepresented pathways amongst the upregulated genes (**Supplementary Table 21**), which were further explored using EnrichmentMap visualization (**Figure 7C****),** and detailed expression data for highlighted gene ontology terms is included in **Supplementary Table 22**.

Review of the pathway analysis (**Figure 6C**) indicates that MYADM+ memory B cells possess an inflammatory signature. The integrin-mediated signaling pathway (GO:0007229) was the most overrepresented with 14/117 upregulated genes (p_adj_ = 2.4x10^-9^) (**Supplementary Tables 21**, **S22**). Integrins ITGB7, ITGB2, and ITGA4 were upregulated in the MYADM+ cells. VLA-4 (ITGB1/ITGA4) interaction with VCAM-1 facilitates B cell activation by membrane bound antigen (54, 55). Other upregulated genes in this pathway include two Src kinases, FGR (FC = 1.9, p_adj_=1.6E-25) and HCK (FC = 1.5, p_adj_=1.8E-6), that were previously shown to drive inflammation in myeloid (56) and B cells (57). The most upregulated gene in this pathway, ADAM15 (FC = 2.0, p_adj_=7.41E-8), mediated cell-cell interactions and pro-inflammatory TNF-alpha mRNA levels in T cells (58). Similarly, the response to tumor necrosis factor pathway (GO:0034612) was overrepresented with upregulation of 11/249 genes (p_adj_=6.6E-4). The regulation of canonical NF-kappaB signaling (GO:0043122) contained 13/309 upregulated genes (p_adj_=1.1E-4). The ILK gene (FC = 1.49, p_adj_=1.09E-5) was identified in all three aforementioned pathways. ILK encodes the integrin-linked kinase, which has been implicated in Akt signaling in T cells (59). Finally, MHC class II antigen presentation pathway (REAC:R-HAS-2132295) was enriched in MYADM+ memory B cells (p_adj_=2.49E-3), containing 9/123 upregulated genes including DYNLL1 (FC 1.39, p_adj_ 5.40E-13). DYNLL1 was reported to be a signal-specific regulator of NF-KB capable of modulating TLR4 signaling and antibody responses (60). STRING analysis in **Figure 6D** comparing MYADM+ and MYADM- B cells identified two groups of differentially expressed genes. The first group was led by IFI30, which encodes gamma-interferon-inducible lysosomal thiol reductase, which was connected to LGMN (legumain), both of which are involved in antigen processing and presentation (reviewed in (61, 62) and identified in the enriched MHC Class II antigen presentation pathway (**Figure 7** and **Supplementary Table 22**). Additional connections include integrins (B2 and B7), FGR (a Src family kinase that counteracts β2 integrin signaling (63)) and CXCR3 (which promotes homing to inflamed tissue) (64). Given that integrin α4β7 is typically expressed by gut-homing B cells, and that gut B cells are chiefly of the IgA and IgM isotypes, we evaluated isotype usage among MYADM-positive and MYADM-negative memory B cells with ASyS patients and healthy controls. While MYADM-positive memory B cells showed a rather modest increase in isotype switching overall, they did not exhibit dramatic skewing towards IgA, relative to MYADM-negative memory B cells in either ASyS patients or controls (**Supplementary Figure 6**). A second group was led by S100A10, which interacts with ANXA4 and S100A11 among other proteins. ANXA4 (Annexin A4) interacts with p50 in a Ca2+-dependent manner to modulate NF-κB transcriptional activity (65). Other annexin family proteins have been shown to work in complex with S100A10 to coordinate plasma membrane phospholipid domains, actin rearrangement, exocytosis and positioning of ion channels and GPCRs at the plasma membrane. ANXA4 and S100A11 also play a role in membrane repair (66).

S100A10 is also connected to CD151, which supports β2 integrin-mediated adhesion, as well as cytoskeletal rearrangement, and is regulated by Bcl6 (reviewed in (67, 68)). GRAMD1C negatively regulates autophagy; deficiency increases mitochondrial cholesterol levels and mitochondrial oxidative phosphorylation (69). SUB1 encodes positive coactivator 4 (PC4), which acts with IKAROS to support IRF4 expression and plasma cell differentiation (70). Together, these gene signatures indicate that MYADM+ memory B cells are an inflammatory subset of memory B cells present in higher frequencies in ASyS. STAG3+ memory B cells differentially expressed only 15 genes compared to the STAG3- memory B cells (**Supplementary Table 23**), so additional analysis of this population was deferred.

## 4 Discussion

Here, we report our findings of differential gene expression in ASyS compared with healthy B cells differ within specific, transcriptionally defined B cell subsets. Our key findings can be summarized as follows: (1) memory B cells in ASyS have gene expression changes and functional evidence of chemical stress and increased levels of reactive oxygen species (2) memory B cells in ASyS have upregulated genes related to cytoskeletal rearrangement, (3) memory B cells in ASyS have upregulated several FK506 binding proteins (FKBP) that may alter cellular proliferation and cell cycle regulation, and (4) a subset of MYADM+ memory B cells in ASyS patients show upregulation of genes related to antigen processing/presentation and cell adhesion. Consistent with a prior report using bulk RNA-sequencing of memory B cells (26), we also observed that memory B cells displayed a type I interferon signature, which was also present in activated B cell subsets. Together, these findings indicate key functional differences in ASyS and healthy activated and memory B cells, which may have important pathologic consequences.

A type 1 interferon signature was previously noted in muscle biopsies and blood samples ASyS patients (71, 72), which tracks with the interferon signature noted in various peripheral blood immune populations, as outlined above. Bulk RNA-sequencing of memory B cells showed upregulation of ER stress response genes in active IIM patients, relative to patients with inactive disease (26). In this same study, DN (atypical memory) B cells showed upregulation of oxidative phosphorylation genes. Upregulation of the oxidative phosphorylation pathway in monocytes and NKT cells was also noted in a separate study (73). These gene expression data track with the differentially expressed genes identified in memory B cells in the current study, as well as the flow cytometry data presented here which identify increased reactive oxygen species in ASyS B cells relative to healthy controls.

Here, we show increased levels of intracellular ROS in ASyS B cells. Reactive oxygen species, particularly H_2_O_2_, are long recognized second messengers in the antigen dependent stimulation of lymphocytes. Antigen stimulation of the BCR increases intracellular H_2_O_2_ production, and H_2_O_2_ inactivates inhibitory protein tyrosine phosphatases to amplify BCR signaling (74). Subsequent studies demonstrated that persistent mitochondrial ROS production after antigen stimulation maintains BCR signal transduction to facilitate optimal antigen induced B cell activation and proliferation (75). ROS are also critical for MHC class II antigen presentation by B cells to CD4+ T cells (76). A limitation of our investigations is that these studies focused on mRNA expression and direct detection of free ROS. Studies of lipid oxidation, oxidation of other key metabolites, and iron homeostasis in ASyS B cells would further increase understanding of how ROS modulate B cell function in ASyS.

Our investigations also uncovered the upregulation of several FK506 binding proteins, including FKBP1A, FKBP3, FKBP5, and the downstream FKBP1A target CCND3. This finding is of particular interest as FK506 (e.g. tacrolimus), is historically thought to be a T cell target, as tacrolimus does not directly decrease immunoglobulin production of healthy B cells (77) or inhibit B cell proliferation and plasma cell differentiation *in vitro* (78). However, we have uncovered upregulation of several FKBPs, which may have important functional consequences in B cells. FKBP3, also known as FKBP25, functions to promote the stabilization and polymerization of the microtubule network and regulated entry into mitosis (79). CCND3, which promotes the G1/S cell cycle transition, is known to be downregulated by rapamycin binding to FKBP12, the protein product of FKBP1A (49). Additionally, FKBP5 has been implicated in lymphocyte co-activation via PD-L1 (39) and endogenous MHC class II antigen presentation (40). Hence, tacrolimus may be acting to limit key B cell functions in ASyS patients as an additional mechanism of action. Additional *in vitro* studies are required to probe how ASyS memory B cells may be modulated by tacrolimus.

Our final key finding is the upregulation of MYADM in memory B cells. While initially described as a marker of granulocyte and macrophage differentiation, MYADM is now known to be a key regulator of membrane rafts in epithelial cells (53) and endothelial cells (80). In HeLa cells, MYADM mediates cell spreading and migration by targeting Rac1 to compact ordered membranes, or membrane rafts (53). The membrane ruffling and cell spreading seen after BCR stimulation in mature B cells is also a Rac1-dependent process (81). RAC1 inhibition limits BCR endocytosis in mice (82) and IgM BCR internalization of large particles in human B cells (83). Additional studies will be required to determine whether MYADM, like RAC1, controls BCR internalization as an initial step in antigen processing and presentation.

This study has several limitations. First, the number of participants – both ASyS and controls – was small and had limited ethnic representation, which means our findings should be further validated in a larger multi-ethnic cohort. Our power to identify gene expression differences is limited by the small sample size and number of sequenced cells, particularly within less abundant B cell subsets. Plasmablasts are a key population that contributes to immune responses, but they make up a relatively low frequency within peripheral blood (24). This population was therefore largely ignored in the current study, given practical limitations. The memory B cell compartment is recognized to be highly complex and consists of many sub-populations known to have different programming and function (reviewed in (24)). In the present study, four clusters were collapsed into a single memory subset for downstream analysis to reveal the most broadly dysregulated genes and pathways. It is therefore possible that at least some of the gene expression changes observed are driven by sub-population skewing within the memory compartment. However, several genes were observed to be upregulated in ASyS patients within activated as well as memory B cell subsets (e.g., FKBP5 and STAG3), suggesting that at least some gene expression changes were present which globally affect aberrant B cell programming in ASyS, independent of memory “subtype” bias.

Overall, our data identify novel and known gene expression changes within activated and memory B cells. These include upregulation of genes and pathways that are involved in BCR internalization, B cell activation, and cell stress response. Reactive oxygen species were increased in ASyS B cells relative to healthy control B cells, in line with gene expression changes observed. Finally, we identify enrichment of FKBP5+ and MYADM+ cells within the ASyS memory compartment, relative to healthy controls, which may impact proliferation, adhesion, and antigen processing of ASyS B cells. Future studies will be required to validate these findings and probe their utility as targets in ASyS.

## MYSTIC Investigators

MYSTIC Investigators are collaborative authors who assisted in patient recruitment and collected primary clinical data necessary to facilitate clinical phenotyping.

Rosemarie B. Dudenhofer, Division of Allergy, Pulmonary, and Critical Care Medicine, Department of Medicine, Vanderbilt University Medical Center, Nashville, TN, USA.

Leslie J. Crofford, Division of Rheumatology and Immunology, Department of Medicine, Vanderbilt University Medical Center, Nashville, TN, USA*.

Justin C. Hewlett, Division of Allergy, Pulmonary, and Critical Care Medicine, Department of Medicine, Vanderbilt University Medical Center, Nashville TN, USA.

Susan Kroop, Division of Rheumatology and Immunology, Department of Medicine, Vanderbilt University Medical Center, Nashville, TN, USA.

Meredith E. Pugh, Division of Allergy, Pulmonary, and Critical Care Medicine, Department of Medicine, Vanderbilt University Medical Center, Nashville TN, USA.

Todd W. Rice, Division of Allergy, Pulmonary, and Critical Care Medicine, Department of Medicine, Vanderbilt University Medical Center, Nashville TN, USA.

Carla M. Sevin, Division of Allergy, Pulmonary, and Critical Care Medicine, Department of Medicine, Vanderbilt University Medical Center, Nashville, TN, USA.

Lorraine B. Ware, Division of Allergy, Pulmonary, and Critical Care Medicine, Department of Medicine, Vanderbilt University Medical Center, Nashville TN, USA.

Melissa Warren, Division of Allergy, Pulmonary, and Critical Care Medicine, Department of Medicine, Vanderbilt University Medical Center, Nashville TN, USA.

Erin M. Wilfong, Division of Rheumatology and Immunology, Department of Medicine, Vanderbilt University Medical Center, Nashville, TN, USA*.

*Denotes members of the Writing Committee.

## Data Availability

The datasets presented in this study can be found in online repositories. The names of the repository/repositories and accession number(s) can be found below: https://www.ncbi.nlm.nih.gov/, NCBI Sequence Read Archive (SRA) under BioProject PRJNA1391566.
