# Supplementary material for: B cells in anti-tRNA synthetase syndrome patients show an activated, interferon-responsive signature
Source: Front Immunol. 2026 Apr 7;17:1770291. doi: 10.3389/fimmu.2026.1770291 (PMC13095816; doi:10.3389/fimmu.2026.1770291)
Supplement: Supplementary file 1 [file Supplementaryfile1.docx]

**Supplemental Materials:**

Table of Contents

[Supplemental Methods: 2](#_Toc223965288)

[Supplemental Table S1. Cite-Seq antibody panel 2](#_Toc223965289)

[Supplemental Figures 3](#_Toc223965290)

[Supplemental Figure S1. Representative gating scheme for Figure 4. 3](#_Toc223965291)

[Supplemental Figure S2. B cell clusters were collapsed into B cell subsets. 4](#_Toc223965292)

[Supplemental Figure S3. Top DEGs for B cell clusters. 5](#_Toc223965293)

[Supplemental Figure S4. Donor level expression frequency of candidate genes in activated and memory B cell subsets. 6](#_Toc223965294)

[Supplemental Figure S5. MYADM expression is increased in ASyS compared to healthy memory B cells. 7](#_Toc223965295)

[Supplemental Figure S6. IgH isotype frequency in MYADM+ memory B cells. 8](#_Toc223965296)

[Supplemental Data Tables: 9](#_Toc223965297)

[Supplemental Table S2. Detailed Clinical Phenotypes 9](#_Toc223965298)

[Supplemental Table S3. Frequency of various B cell subsets 11](#_Toc223965299)

[Supplemental Table S4. Differentially Expressed Genes in Transitional B cells in ASyS compared to HC 12](#_Toc223965300)

[Supplemental Table S5. Differentially Expressed Genes in Naïve B cells in ASyS compared to HC 13](#_Toc223965301)

[Supplemental Table S6. Differentially Expressed Genes in Activated B cells in ASyS compared to HC 15](#_Toc223965302)

[Supplemental Table S7. Differentially Expressed Genes in Memory B cells in ASyS Compared to HC 17](#_Toc223965303)

[Supplemental Table S8. Overrepresented pathways in activated B cells. Upregulated genes with fold change increase > 1.25 and FDR q<0.01 in ASyS compared to HC were included in g:profiler analysis. 21](#_Toc223965304)

[Supplemental Table S9. Detailed expression data for featured GO pathways in activated B cells for ASyS v. HC 23](#_Toc223965305)

[Supplemental Table S10. Top up- and down-regulated genes for activated B cells in ASyS compared to HC 24](#_Toc223965306)

[Supplemental Table S11. Overrepresented pathways in memory B cells. Upregulated genes with fold change increase > 1.25 and FDR q<0.01 in ASyS compared to HC were included in g:profiler analysis. 25](#_Toc223965307)

[Supplemental Table S12. Detailed expression data for featured GO pathways in memory B cells for ASyS v. HC 28](#_Toc223965308)

[Supplemental Table S13 Top up- and down-regulated genes for memory B cells in ASyS v. HC 29](#_Toc223965309)

[Supplemental Table S14. Raw data for Figure 4 30](#_Toc223965310)

[Supplemental Table S15. Genes with increased frequency of expression for activated and memory subsets in ASyS versus HC. 31](#_Toc223965311)

[Supplemental Table 16. Donor Level Frequency of Candidate Gene Expression 32](#_Toc223965312)

[Supplemental Table S17. Differentially Expressed Genes in FKBP5+ compared to FKBP5- Memory B cells 33](#_Toc223965313)

[Supplemental Table S18. Transcriptional changes for genes identified by STRING analysis in FKBP5+ memory B cells compared to FKBP5-Memory B cells 34](#_Toc223965314)

[Supplemental Table 19. Differences in healthy (HC) versus antisynthetase syndrome (ASyS) memory B cell expression of FK506 targets and associated regulator proteins 35](#_Toc223965315)

[Supplemental Table S20. Differentially Expressed Genes in MYADM+ versus MYADM- Memory B cells 36](#_Toc223965316)

[Supplemental Table S21. Overrepresented pathways in MYADM+ memory B cells. Upregulated genes with fold change increase > 1.25 and FDR q<0.01 were included in g:profiler analysis. 39](#_Toc223965317)

[Supplemental Table S22. Detailed expression data for featured GO pathways in MYADM+ memory B cells 43](#_Toc223965318)

[Supplemental Table S23. Differentially expressed genes for STAG3+ compared to STAG3- memory B cells using cut-offs FC > 1.25 and padj < 0.01 44](#_Toc223965319)

| Supplemental Methods:Supplemental Table S1. Cite-Seq antibody panel | | | | | | | |
| --- | --- | --- | --- | --- | --- | --- | --- |
| Format | Target: | Clone | Cat # | Company | Dilution | [Final] | Volume |
| TotSeq C | C0251 antihuman hashtag 1 | LNH-94/2M2 | 394661 | BioLegend | 1:500 | 1ug/mL | 0.20 |
| TotSeq C | C0251 antihuman hashtag 2 | LNH-94/2M2 | 394663 | BioLegend | 1:500 | 1ug/mL | 0.20 |
| TotSeq C | C0251 antihuman hashtag 5 | LNH-94/2M2 | 394669 | BioLegend | 1:500 | 1ug/mL | 0.20 |

# Supplemental Figures


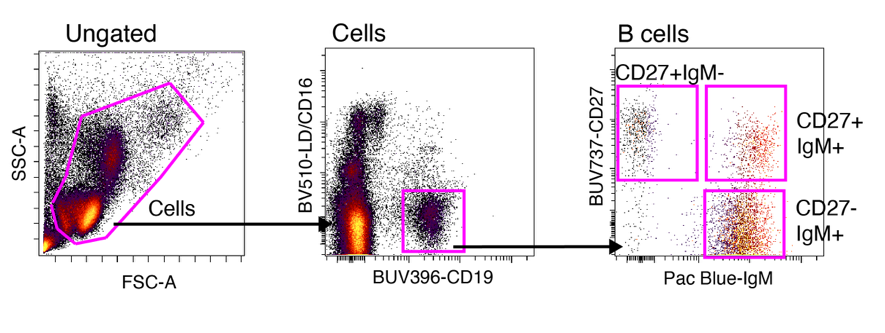


Supplemental Figure S1. Representative gating scheme for Figure 4. The CD19+CD27-IgM+, CD19+CD27+IgM+, and CD19+CD27+IgM- B cell subsets were defined as shown. Mean fluorescent intensity for PE (MitoSOX, TMRE), and FITC (Mitotracker Green, DCFDA) were determined for each subset


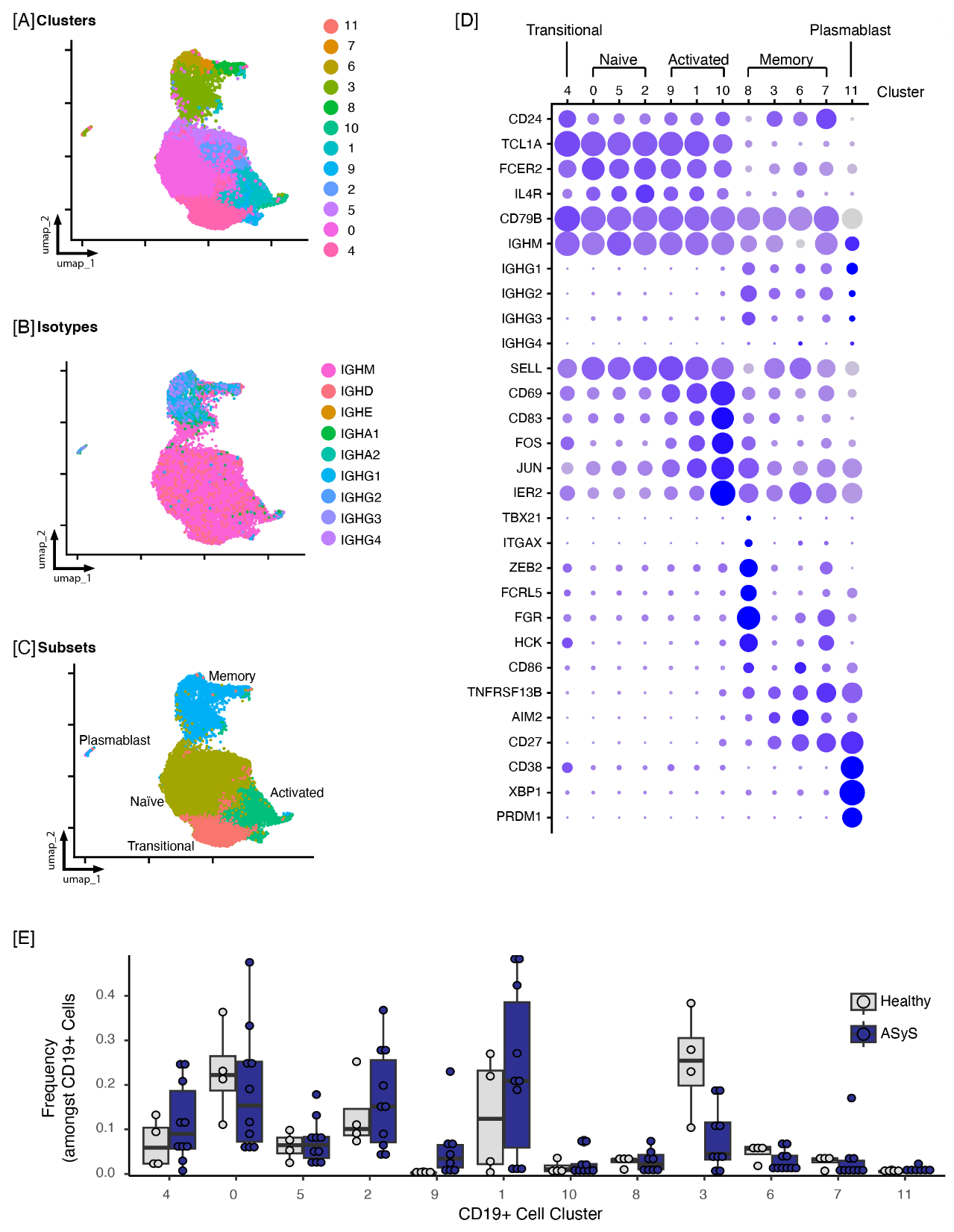


Supplemental Figure S2. B cell clusters were collapsed into B cell subsets. Seurat identified 12 clusters which were further grouped as naïve, transitional, activated, memory, and plasmablast subsets based on manual inspection of isotype and gene expression profiles for each cluster. [A] UMAP showing RNA-seq-based B cell clusters. [B] Isotype distribution based on immunoglobulin heavy chain genes is overlaid onto the cluster UMAP. [C] Overlay of collapsed subsets onto the cluster UMAP. [D] Manually selected gene expression profiles for cells grouped by B cell cluster which can be further collapsed into B cell subsets. A heatmap of top differentially expressed gene expression is shown in Supplemental Figure S3. [E] Boxplots show the mean frequency of each B cell cluster by disease group, with individual donors are plotted as points. Due to the low number of cells isolated from HC2, it was excluded from the frequency calculations shown in [E].

Supplemental Figure S3. Top DEGs for each B cell cluster. Purified CD19+ CD3- cells were isolated from n = 5 Jo-1 ASyS patients, n=5 non-Jo-1 ASyS patients and n = 5 healthy donors and profiled using single-cell RNA-seq technology as in Fig. 1 and Methods. The top 10 differentially expressed genes for each cluster identified as in Supplemental Figure S2 are shown here.


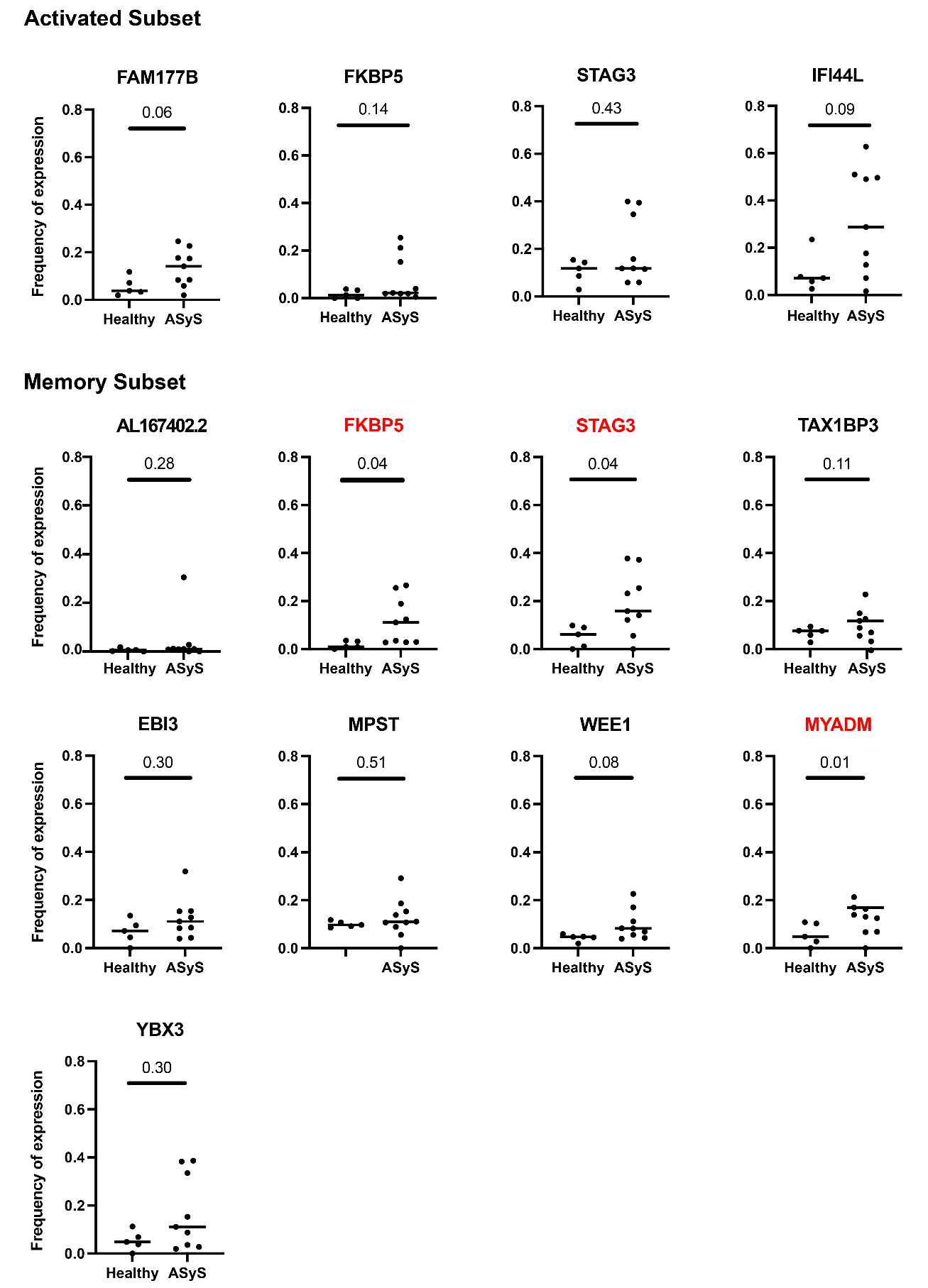


Supplemental Figure S4. Donor level expression frequency of candidate genes in activated and memory B cell subsets. Candidate genes for this analysis were defined as genes expressed in < 10% of healthy cells and for which the percent of ASyS B cells expressing that gene increased by > 10% compared to healthy cells. ASyS4 was excluded from the activated subset analysis and ASyS10 was excluded from the memory analysis as those participants had fewer than 10 cells detected for the subset under investigation.


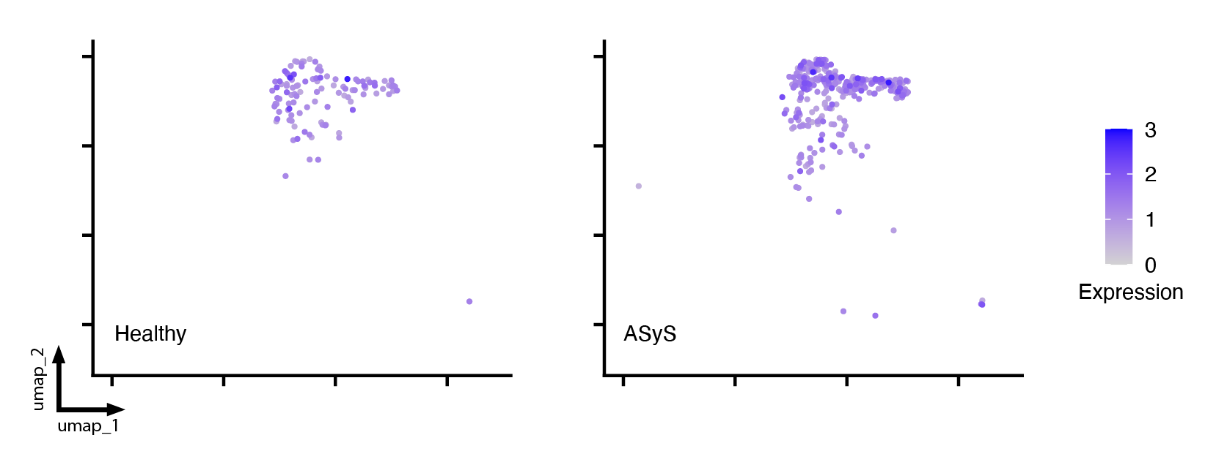


Supplemental Figure S5. MYADM+ B cells are identified in ASyS and healthy memory B cells. UMAPs show MYADM gene expression across healthy (left) or ASyS (right) memory B cells (identified as in Fig. 1) categorized as MYADM-expressing cells based on normalized MYADM expression level greater than 0.5 in the Seurat object data slot. Cells with MYADM expression less than 0.5 were categorized as not expressing MYADM and are not shown.


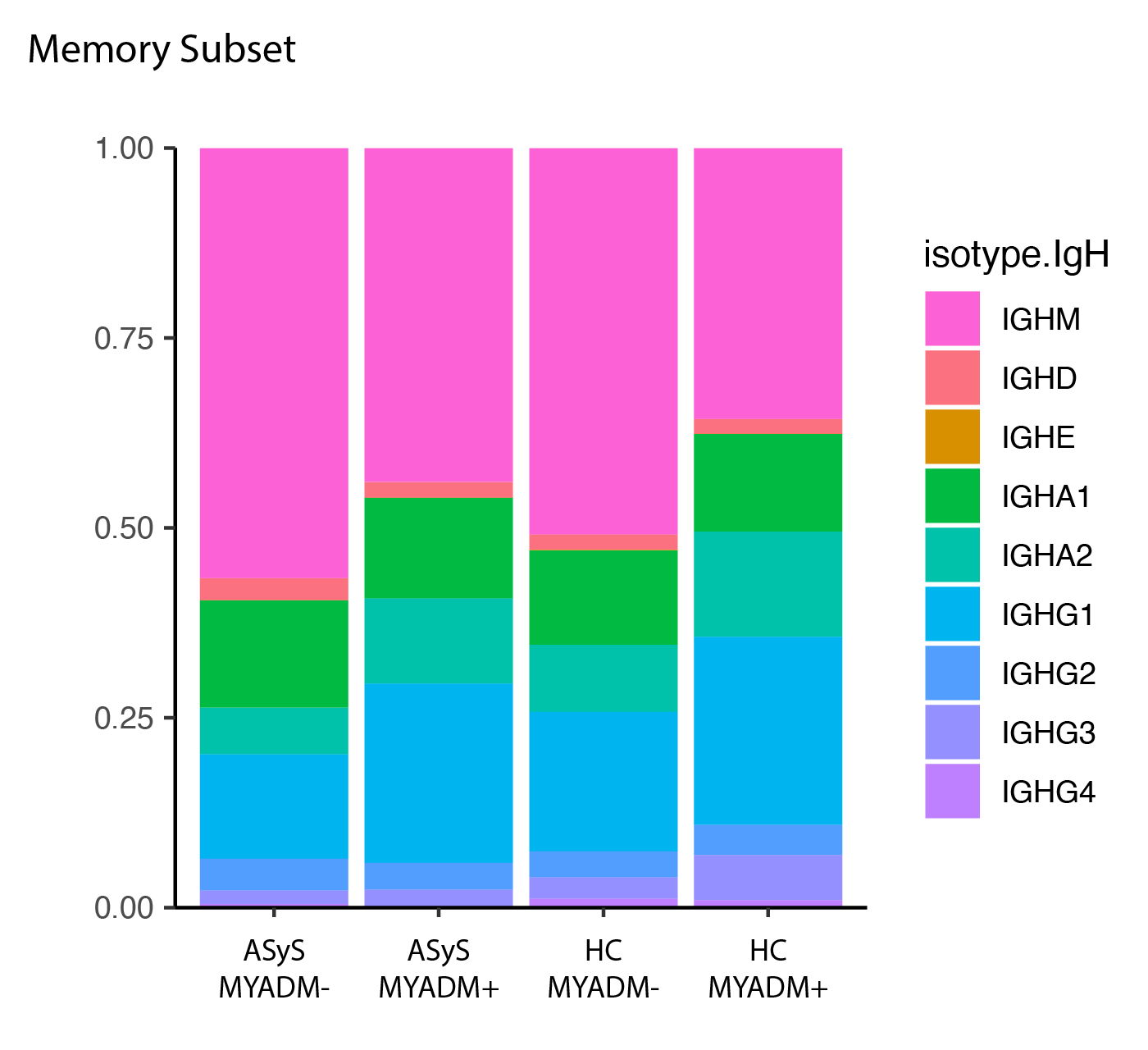


Supplemental Figure S6. IgH isotype frequency in MYADM+ memory B cells. BCR heavy chain isotype was assigned based on RNA-seq counts as in Fig. 1C. Isotype distribution is shown within MYADM- or MYADM+ memory B cells defined in **Figure 7** among ASyS or HC donors.

# Supplemental Data Tables:

| Supplemental Table S2. Detailed Clinical Phenotypes | | | | | | | | | | | | | | |
| --- | --- | --- | --- | --- | --- | --- | --- | --- | --- | --- | --- | --- | --- | --- |
| Participant | Age | Sex | Disease  Duration  (mos) | Ancestry | Myositis  Antibody | Proximal Muscle Weakness | Arthritis | Skin Rashes | ILD | FVC at enroll | DLCO at enroll | Supplemental O2 | Current  Meds† | Meets 2017 IIM criteria |
| ASyS1 | 45-49 | M | 48.0 | European | Jo | Yes | Yes | Gottron’s  Shawl  Mechanic’s hands | Yes | n.d. | N.d. | Intubated, ECMO | Prednisone 60mg x 2 weeks,  IVIG 2g/kg (day 2/5) | yes |
| ASyS2 | 45-49 | M | 12.3 | Black | Jo | Yes | Yes | Heliotrope  Mechanic’s hands | Yes | 59% | 30% | Rest 2L  Exertion 3L | Methylpred 1g/day (day 2/3) | yes |
| ASyS3 | 55-59 | M | 85.6 | Black | PL7 | No | No | None | Yes | 57% | 45% | None | None | No |
| ASyS4 | 50-54 | M | 94.0 | European | Jo1 | No | Yes | Gottron’s  Mechanic’s hands | Yes | 57% | 59% | None | None | Yes |
| ASyS5 | 55-59 | M | 15.9 | Black | PL12, Ro52 | Yes | Yes | Mechanic’s Hands | Yes | 67% | 39% | None | None | No |
| ASyS6 | 70-74 | F | 10.0 | European | Jo1 | No | Yes | None | Yes | n.d. | n.d. | Rest 45L  Exertion 60L | Prednisone 60mg (day 2) | Yes |
| ASyS7 | 55-59 | F | 5.7 | European | Jo1 | No | Yes | Heliotrope  Mechanic’s hands | Yes | 56% | 56% | Rest 2L  Exertion 6L | Prednisone 20 mg/day | Yes |
| ASyS8 | 35-39 | F | 36.0 | Black | PL12, Ro52 | Yes | No | None | Yes | 43% | 23% | None | Prednisone 5 mg/day,  AZA 150 mg/day | Yes |
| ASyS9 | 70-74 | F | 11.0 | European | PL12, Ro52 | No | No | None | Yes | 53% | 30% | Rest 3L  Exertion 3L | Prednisone 10 mg/day | No |
| ASyS10 | 35-39 | F | 12.0 | Black | PL12, Ro52 | No | Yes | Mechanic’s hands | Yes | 35% | 33% | None | Prednisone 40 mg every other day  MMF 1g/day‡ | No |
| HC1 | 55-59 | F |  | European |  |  |  |  |  |  |  |  |  |  |
| HC2 | 60-64 | F |  | European |  |  |  |  |  |  |  |  |  |  |
| HC3 | 35-39 | F |  | Black |  |  |  |  |  |  |  |  |  |  |
| HC4 | 40-44 | M |  | European |  |  |  |  |  |  |  |  |  |  |
| HC5 | 50-54 | M |  | Black |  |  |  |  |  |  |  |  |  |  |
| †Medications at time of enrollment. For medications that have been started within two weeks of enrollment/sample collection, duration of therapy is also listed  ‡Patient reported taking the medication only a few times per week.  ASyS=antisynthetase syndrome, HC=healthy control | | | | | | | | | | | | | | |

| Supplemental Table S3. Frequency of various B cell subsets | | | | | | |
| --- | --- | --- | --- | --- | --- | --- |
| Participant* | # Cells | Subset Frequency | | | | |
|  |  | Activated | Memory | Naïve | Plasmablast | Transitional |
| ASyS1 | 942 | 0.196 | 0.200 | 0.534 | 0.001 | 0.069 |
| ASyS2 | 1766 | 0.230 | 0.503 | 0.140 | 0.016 | 0.111 |
| ASyS3 | 2059 | 0.300 | 0.289 | 0.390 | 0.014 | 0.007 |
| ASyS4 | 448 | 0.018 | 0.210 | 0.647 | 0.004 | 0.121 |
| ASyS5 | 1003 | 0.475 | 0.018 | 0.299 | 0.000 | 0.208 |
| ASyS6 | 2136 | 0.024 | 0.066 | 0.879 | 0.002 | 0.029 |
| ASyS7 | 1582 | 0.032 | 0.064 | 0.841 | 0.000 | 0.063 |
| ASyS8 | 1749 | 0.580 | 0.021 | 0.147 | 0.0000 | 0.253 |
| ASyS9 | 661 | 0.619 | 0.129 | 0.175 | 0.023 | 0.054 |
| ASyS10 | 100 | 0.510 | 0.010 | 0.230 | 0.010 | 0.240 |
| HC1 | 867 | 0.308 | 0.404 | 0.255 | 0.010 | 0.023 |
| HC3 | 1606 | 0.011 | 0.139 | 0.714 | 0.004 | 0.133 |
| HC4 | 1671 | 0.229 | 0.359 | 0.312 | 0.005 | 0.095 |
| HC5 | 605 | 0.043 | 0.511 | 0.418 | 0.005 | 0.023 |
| *HC2 had a low number of recovered B cells, and as such was excluded from subset frequency calculations. | | | | | | |

| Supplemental Table S4. Differentially Expressed Genes in Transitional B cells in ASyS compared to HC | | | | | |
| --- | --- | --- | --- | --- | --- |
| Upregulated in Transitional ASyS B Cells | | | Downregulated in Transitional ASyS B Cells | | |
| Gene | Fold Change | padj | Gene | Fold Change | padj |
| CCND3 | 1.91 | 5.87E-07 | ADK | 1.58 | 3.02E-06 |
| CD69 | 3.30 | 4.85E-34 | ANXA4 | 1.63 | 1.84E-05 |
| CXCR4 | 1.66 | 1.33E-09 | CD180 | 1.46 | 9.12E-03 |
| DDIT4 | 3.16 | 1.23E-03 | FCER2 | 2.02 | 3.52E-28 |
| DUSP1 | 2.56 | 3.99E-34 | ID3 | 2.00 | 2.39E-05 |
| EIF2AK2 | 2.46 | 6.82E-03 | MT-CYB | 1.40 | 1.70E-33 |
| EPSTI1 | 2.57 | 6.73E-07 | MT-ND3 | 1.27 | 2.07E-20 |
| FKBP5 | 13.80 | 6.75E-04 | PTPN6 | 1.37 | 6.33E-08 |
| FOS | 2.52 | 2.70E-14 | RASSF6 | 51.50 | 1.23E-03 |
| GADD45B | 1.95 | 3.14E-09 | RIMBP2 | 5.40 | 2.95E-03 |
| H3F3B | 1.35 | 4.75E-10 | RPS4X | 1.31 | 1.55E-46 |
| IFI44L | 17.29 | 4.05E-27 | SELL | 1.38 | 6.82E-04 |
| IFITM1 | 5.04 | 2.88E-43 | SNHG5 | 2.00 | 2.00E-06 |
| IFITM2 | 1.50 | 3.35E-10 | TUBA1B | 1.40 | 4.85E-03 |
| ISG20 | 1.31 | 1.51E-03 | XIST | 4.10 | 7.48E-14 |
| JUNB | 1.49 | 3.10E-07 |  |  |  |
| KLF6 | 2.28 | 2.89E-08 |  |  |  |
| LY6E | 1.66 | 3.77E-15 |  |  |  |
| MX1 | 2.45 | 2.62E-11 |  |  |  |
| PLSCR1 | 3.62 | 7.76E-04 |  |  |  |
| RPS26 | 1.50 | 1.54E-20 |  |  |  |
| SMDT1 | 1.34 | 2.19E-04 |  |  |  |
| TLE1 | 1.74 | 3.06E-03 |  |  |  |
| TRIM22 | 1.56 | 4.38E-04 |  |  |  |
| TSC22D3 | 2.64 | 8.81E-28 |  |  |  |
| TXNIP | 1.72 | 3.44E-05 |  |  |  |
| VIM | 1.32 | 1.86E-03 |  |  |  |
| XAF1 | 3.05 | 3.98E-11 |  |  |  |
| ZFP36L2 | 2.02 | 2.01E-04 |  |  |  |

| Supplemental Table S5. Differentially Expressed Genes in Naïve B cells in ASyS compared to HC | | | | | |
| --- | --- | --- | --- | --- | --- |
| Upregulated in Naïve ASyS B Cells | | | Downregulated in Naïve ASyS B Cells | | |
| Gene | Fold Change | padj | Gene | Fold Change | padj |
| AC245014.3 | 2.21 | 3.44E-10 | AC091045.1 | 1.57 | 1.96E-03 |
| AC253572.2 | 2.52 | 8.59E-21 | AC245407.2 | 1.52 | 2.17E-03 |
| AL021155.5 | 3.14 | 8.21E-11 | AIM2 | 2.44 | 2.10E-03 |
| CCDC18-AS1 | 1.44 | 2.00E-13 | AL034397.3 | 1.58 | 7.92E-11 |
| CCL4 | 3.86 | 2.09E-05 | AL139020.1 | 1.85 | 5.79E-28 |
| CCND3 | 1.82 | 1.64E-47 | ANXA4 | 1.52 | 3.66E-11 |
| CD69 | 2.21 | 6.29E-51 | ARHGAP24 | 1.40 | 1.95E-20 |
| CNDP2 | 1.45 | 3.80E-05 | ATP6AP2 | 1.27 | 7.29E-05 |
| CNTNAP2 | 2.24 | 7.41E-09 | CBWD1 | 1.43 | 2.32E-03 |
| CXCR4 | 1.27 | 1.91E-07 | CCN2 | 3.00 | 3.98E-03 |
| DDIT4 | 2.94 | 4.82E-31 | CD180 | 1.29 | 5.58E-07 |
| DUSP1 | 1.86 | 1.88E-42 | CD27 | 1.99 | 2.85E-03 |
| FAM118A | 2.23 | 4.14E-03 | CD96 | 1.93 | 2.95E-07 |
| FKBP5 | 11.53 | 1.85E-36 | CDHR3 | 2.11 | 3.20E-03 |
| FOS | 1.67 | 9.57E-06 | CLEC4A | 1.55 | 2.68E-04 |
| GBP4 | 1.68 | 1.55E-03 | EIF5A | 1.53 | 2.81E-42 |
| GLO1 | 1.55 | 1.30E-11 | ENOSF1 | 3.97 | 6.22E-06 |
| GRHPR | 1.43 | 4.66E-04 | FAM43A | 1.58 | 2.74E-04 |
| GSTP1 | 1.28 | 1.15E-06 | FCER2 | 1.43 | 2.29E-74 |
| HAGHL | 2.89 | 8.18E-05 | FGR | 1.40 | 4.80E-06 |
| IFI44L | 3.65 | 4.44E-14 | GALNT2 | 1.48 | 4.92E-08 |
| IFITM1 | 1.99 | 4.04E-59 | GM2A | 1.62 | 1.17E-15 |
| IL13RA1 | 2.81 | 2.73E-06 | GTF2H2 | 1.46 | 1.38E-04 |
| IRF7 | 1.47 | 1.91E-04 | HACD4 | 1.38 | 1.24E-03 |
| ITGAE | 1.36 | 5.13E-03 | HCST | 1.61 | 5.84E-05 |
| JUN | 1.35 | 2.20E-10 | HIST1H2AC | 1.42 | 8.02E-10 |
| KLRK1 | 7.86 | 8.39E-24 | HIST1H2BC | 1.70 | 1.05E-05 |
| LY6E | 1.29 | 1.33E-07 | HIST1H2BF | 1.72 | 3.89E-04 |
| NEAT1 | 1.87 | 8.00E-06 | HOPX | 1.86 | 2.51E-06 |
| OAS1 | 1.68 | 2.48E-06 | HSD17B10 | 1.37 | 2.25E-06 |
| P2RX5 | 1.85 | 5.74E-47 | ID3 | 1.72 | 4.52E-17 |
| PAX8-AS1 | 2.46 | 7.20E-08 | IL21R | 1.53 | 1.38E-03 |
| PDXDC1 | 1.80 | 9.67E-04 | IL3RA | 2.79 | 7.66E-05 |
| PIK3IP1 | 1.42 | 1.50E-04 | ITGB2-AS1 | 1.38 | 1.37E-03 |
| PLP2 | 1.32 | 1.93E-05 | JUP | 1.67 | 5.86E-18 |
| PRCD | 2.09 | 8.93E-04 | LHPP | 1.32 | 7.10E-06 |
| PTCH2 | 2.79 | 3.65E-03 | LINC01013 | 3.49 | 2.32E-23 |
| RAB24 | 1.33 | 3.76E-03 | LITAF | 1.26 | 2.27E-03 |
| RCSD1 | 1.41 | 6.27E-42 | MACROD2 | 1.71 | 1.52E-17 |
| RHOB | 1.86 | 2.07E-06 | MPEG1 | 1.35 | 7.06E-03 |
| RPS26 | 1.57 | 2.24E-154 | MYL9 | 3.89 | 2.33E-07 |
| S100A4 | 1.56 | 1.28E-03 | PLD4 | 1.97 | 1.69E-39 |
| SESN1 | 2.50 | 4.81E-33 | POLR2J2 | 3.66 | 5.94E-09 |
| SMAP2 | 1.32 | 2.39E-03 | PSMC6 | 1.37 | 3.53E-06 |
| SMDT1 | 1.30 | 2.54E-15 | RIMBP2 | 17.99 | 2.20E-08 |
| SNHG8 | 1.25 | 5.40E-09 | RNF125 | 1.75 | 8.35E-03 |
| SOCS1 | 2.40 | 1.79E-07 | SCIMP | 1.78 | 1.13E-21 |
| STAG3 | 3.60 | 2.20E-54 | SMIM10 | 5.18 | 7.74E-06 |
| STK17A | 1.26 | 8.08E-10 | SS18L2 | 1.26 | 7.89E-03 |
| TFEB | 1.76 | 3.32E-07 | TLE5 | 1.29 | 1.98E-10 |
| TLE1 | 1.83 | 1.47E-13 | TMEM156 | 1.30 | 3.75E-05 |
| TNFSF8 | 4.55 | 8.42E-05 | TMEM204 | 3.51 | 9.87E-06 |
| TP53INP1 | 1.98 | 4.02E-07 |  |  |  |
|  | | | | | |
| Supplemental Table S5. Differentially Expressed Genes in Naïve B cells in ASyS compared to HC (continued.) | | | | | |
| Upregulated in Naïve ASyS B Cells | | |  |  |  |
| Gene | Fold Change | padj |  |  |  |
| TRIM22 | 1.33 | 7.80E-07 |  |  |  |
| TSC22D3 | 1.59 | 4.81E-62 |  |  |  |
| TXNIP | 1.68 | 2.17E-86 |  |  |  |
| UACA | 3.26 | 4.96E-11 |  |  |  |
| USP53 | 2.56 | 2.78E-07 |  |  |  |
| XAF1 | 1.71 | 1.48E-07 |  |  |  |
| YBX3 | 1.81 | 2.30E-10 |  |  |  |
| Z93241.1 | 2.40 | 3.80E-08 |  |  |  |
| ZBTB16 | 3.67 | 4.78E-25 |  |  |  |
| ZFP36L2 | 1.41 | 9.68E-09 |  |  |  |

| Supplemental Table S6. Differentially Expressed Genes in Activated B cells in ASyS compared to HC | | | | | |
| --- | --- | --- | --- | --- | --- |
| Upregulated in Naïve ASyS B Cells | | | Downregulated in Naïve ASyS B Cells | | |
| Gene | Fold Change | padj | Gene | Fold Change | padj |
| ABRACL | 1.67 | 2.23E-03 | ABO | 18.06 | 1.15E-08 |
| AC245014.3 | 2.30 | 4.54E-05 | ANXA4 | 1.89 | 1.10E-15 |
| AC253572.2 | 2.66 | 2.12E-06 | BCL2A1 | 2.06 | 8.41E-04 |
| ANAPC16 | 1.29 | 2.86E-07 | CALHM6 | 1.25 | 7.34E-03 |
| ATP5MD | 1.33 | 1.48E-03 | CD82 | 1.44 | 1.15E-05 |
| ATP6V0E1 | 1.32 | 2.03E-04 | DNASE1L3 | 2.26 | 1.43E-03 |
| BST2 | 1.51 | 2.74E-06 | EEF2 | 1.25 | 5.60E-34 |
| CCND3 | 1.63 | 2.31E-13 | EIF5A | 1.39 | 5.34E-07 |
| CD24 | 1.69 | 6.30E-12 | FAM111B | 2.94 | 9.31E-03 |
| CD53 | 1.42 | 2.31E-15 | FCER2 | 1.65 | 4.76E-40 |
| CD69 | 2.23 | 3.62E-65 | HIST1H2AC | 1.62 | 2.06E-06 |
| CNTNAP2 | 6.89 | 5.24E-06 | HOPX | 1.95 | 3.49E-03 |
| COX5A | 1.50 | 7.13E-04 | IL10RA | 1.80 | 1.10E-04 |
| CRIP1 | 1.89 | 9.92E-03 | MT-CYB | 1.39 | 6.86E-68 |
| CYB5A | 1.59 | 3.53E-03 | NFKBID | 1.96 | 1.90E-03 |
| DDIT4 | 2.34 | 4.43E-05 | POLR2J2 | 5.93 | 7.51E-11 |
| DRAP1 | 1.44 | 1.44E-03 | RPS26 | 1.29 | 3.02E-59 |
| DUSP1 | 1.80 | 1.76E-38 | SBDS | 1.50 | 6.95E-03 |
| EIF2AK2 | 2.22 | 1.09E-05 | SGK1 | 2.65 | 3.29E-04 |
| EPSTI1 | 2.32 | 1.07E-07 | SNED1 | 5.21 | 8.00E-03 |
| FAM177B | 5.23 | 1.36E-10 | SNX9 | 1.97 | 5.71E-05 |
| FKBP5 | 5.13 | 3.55E-04 | TLE5 | 1.38 | 2.59E-05 |
| FOS | 1.64 | 8.78E-10 |  |  |  |
| IFI44L | 7.98 | 8.31E-41 |  |  |  |
| IFI6 | 2.53 | 2.30E-04 |  |  |  |
| IFITM1 | 3.69 | 1.91E-70 |  |  |  |
| IFITM2 | 1.74 | 4.44E-38 |  |  |  |
| IFT57 | 1.61 | 3.98E-03 |  |  |  |
| IRF7 | 1.92 | 1.64E-07 |  |  |  |
| ISG15 | 2.35 | 1.13E-03 |  |  |  |
| ISG20 | 1.32 | 8.84E-08 |  |  |  |
| JUN | 1.49 | 5.66E-11 |  |  |  |
| KLF6 | 1.43 | 1.62E-05 |  |  |  |
| LBH | 1.40 | 1.52E-07 |  |  |  |
| LY6E | 1.54 | 3.04E-16 |  |  |  |
| MX1 | 2.42 | 1.98E-16 |  |  |  |
| MX2 | 1.89 | 8.19E-04 |  |  |  |
| MYL12A | 1.33 | 1.72E-13 |  |  |  |
| OAS1 | 2.21 | 1.17E-08 |  |  |  |
| P2RX5 | 2.09 | 7.15E-27 |  |  |  |
| PDXDC1 | 2.84 | 7.95E-04 |  |  |  |
| PLSCR1 | 2.40 | 3.03E-04 |  |  |  |
| PSMB9 | 1.73 | 1.67E-25 |  |  |  |
| RAC2 | 1.31 | 6.50E-16 |  |  |  |
| RCSD1 | 1.30 | 5.02E-05 |  |  |  |
| RHOB | 2.62 | 2.98E-13 |  |  |  |
| RNASE6 | 2.46 | 3.50E-12 |  |  |  |
| S100A6 | 1.48 | 4.47E-06 |  |  |  |
| SESN1 | 2.24 | 2.68E-04 |  |  |  |
| SHISA5 | 1.42 | 2.32E-04 |  |  |  |
| SNHG6 | 1.28 | 1.11E-06 |  |  |  |
| SOCS1 | 3.35 | 7.77E-03 |  |  |  |
| STAG3 | 5.21 | 4.86E-14 |  |  |  |
|  | | | | | |
| Supplemental Table S6. Differentially Expressed Genes in Activated B cells in ASyS compared to HC (continued) | | | | | |
| Upregulated in Activated ASyS B Cells | | |  |  |  |
| Gene | Fold Change | padj |  |  |  |
| STAT1 | 2.69 | 9.37E-05 |  |  |  |
| TMSB4X | 1.33 | 5.34E-54 |  |  |  |
| TRIM22 | 1.54 | 4.33E-06 |  |  |  |
| TSC22D3 | 1.39 | 2.76E-25 |  |  |  |
| TXNIP | 1.37 | 6.37E-04 |  |  |  |
| USP18 | 11.81 | 2.80E-03 |  |  |  |
| VIM | 1.49 | 1.68E-11 |  |  |  |
| WDR83OS | 1.42 | 9.46E-04 |  |  |  |
| XAF1 | 4.15 | 6.49E-39 |  |  |  |

| Supplemental Table S7. Differentially Expressed Genes in Memory B cells in ASyS Compared to HC | | | | | |
| --- | --- | --- | --- | --- | --- |
| Upregulated in Activated ASyS B Cells | | | Downregulated in Naïve ASyS B Cells | | |
| Gene | Fold Change | padj | Gene | Fold Change | padj |
| ABRACL | 1.88 | 2.34E-19 | AC004687.1 | 1.67 | 2.05E-10 |
| AC020916.1 | 4.01 | 2.52E-04 | ADAM28 | 1.46 | 1.45E-08 |
| AC025164.1 | 1.64 | 6.16E-07 | ALOX5 | 1.32 | 6.80E-04 |
| AC090152.1 | 2.82 | 1.12E-04 | ARHGAP24 | 1.33 | 3.56E-05 |
| ACAA2 | 1.36 | 3.42E-03 | BIRC3 | 1.49 | 4.53E-11 |
| ACTG1 | 1.31 | 1.75E-13 | BTG2 | 1.50 | 3.16E-15 |
| ACYP2 | 1.45 | 1.27E-05 | CALHM6 | 1.86 | 4.62E-34 |
| ADGRE5 | 2.22 | 4.50E-29 | CD40 | 1.38 | 4.14E-08 |
| AFMID | 2.29 | 1.46E-03 | CFL2 | 2.34 | 7.91E-03 |
| AGPAT4 | 2.60 | 6.89E-03 | FCER2 | 1.90 | 1.19E-17 |
| AHNAK | 1.53 | 7.71E-15 | IRF1 | 1.90 | 7.51E-12 |
| AIDA | 1.50 | 1.28E-06 | JCHAIN | 1.26 | 3.32E-09 |
| AKAP13 | 1.40 | 1.24E-09 | LINC00926 | 1.34 | 6.13E-20 |
| AL133467.1 | 1.99 | 2.02E-03 | LTB | 1.55 | 1.12E-75 |
| AL157402.2 | 27.08 | 2.46E-39 | LY9 | 1.55 | 2.16E-03 |
| AL592429.2 | 2.51 | 6.93E-06 | NFKBIA | 1.30 | 1.08E-04 |
| ALOX5AP | 1.34 | 5.06E-06 | NFKBID | 2.24 | 5.21E-10 |
| ANAPC16 | 1.29 | 5.77E-19 | NIBAN3 | 1.28 | 3.54E-05 |
| ANG | 4.05 | 1.10E-08 | NPC2 | 1.33 | 4.26E-05 |
| ANKRD28 | 2.50 | 3.26E-03 | NUAK2 | 1.54 | 2.19E-03 |
| ANXA2 | 1.45 | 2.32E-08 | PNRC1 | 1.41 | 6.19E-33 |
| AP1M1 | 1.33 | 1.99E-03 | PNRC2 | 1.39 | 1.12E-04 |
| AP2S1 | 1.42 | 3.54E-11 | RELB | 1.62 | 1.27E-09 |
| ARAP2 | 1.44 | 9.36E-03 | RPS27 | 1.28 | 8.31E-122 |
| ARL4C | 1.57 | 9.32E-05 | RUBCNL | 1.43 | 7.77E-05 |
| ARPC1B | 1.34 | 1.31E-23 | SELENOM | 1.49 | 2.81E-03 |
| ARPC4 | 1.27 | 8.76E-08 | SELL | 1.35 | 1.20E-14 |
| ARPC5 | 1.50 | 3.73E-23 | SYPL1 | 1.28 | 1.78E-08 |
| ARPC5L | 1.46 | 2.42E-10 | TAPBP | 1.37 | 6.30E-12 |
| ASAH1 | 1.37 | 1.63E-07 | TCF7 | 3.23 | 1.03E-05 |
| ASCC1 | 2.46 | 1.00E-12 | TEAD2 | 2.65 | 6.09E-08 |
| ASL | 1.60 | 4.56E-03 | TMEM156 | 1.55 | 4.92E-09 |
| ATG101 | 1.57 | 1.61E-05 | TMEM204 | 10.32 | 3.35E-07 |
| ATP5F1C | 1.26 | 6.35E-05 | TOP1MT | 1.76 | 5.40E-03 |
| ATP5MC3 | 1.27 | 3.48E-10 | U2AF1L5 | 4.55 | 1.20E-14 |
| ATP5MD | 1.26 | 4.17E-08 | VPREB3 | 1.75 | 8.89E-32 |
| ATP5MF | 1.35 | 1.34E-14 |  |  |  |
| ATP6V0D1 | 1.47 | 6.76E-10 |  |  |  |
| BID | 1.57 | 1.46E-03 |  |  |  |
| BLK | 1.33 | 4.44E-19 |  |  |  |
| BRK1 | 1.33 | 8.15E-17 |  |  |  |
| C12orf75 | 1.45 | 8.35E-03 |  |  |  |
| C4orf48 | 1.47 | 1.67E-10 |  |  |  |
| CA5B | 1.63 | 3.11E-06 |  |  |  |
| CALM2 | 1.29 | 1.32E-15 |  |  |  |
| CAP1 | 1.26 | 1.09E-08 |  |  |  |
| CAPNS1 | 1.56 | 4.80E-04 |  |  |  |
| CASP4 | 1.33 | 1.17E-05 |  |  |  |
| CCDC167 | 1.58 | 1.62E-03 |  |  |  |
| CCDC88A | 1.98 | 1.24E-07 |  |  |  |
| CCND3 | 2.61 | 1.33E-102 |  |  |  |
| CD151 | 2.81 | 8.65E-16 |  |  |  |
| CD1C | 1.81 | 2.33E-13 |  |  |  |
|  |  |  |  |  |  |
| Supplemental Table S7. Differentially Expressed Genes in Memory B cells in ASyS Compared to HC (continued) | | | | | |
| Upregulated in Activated ASyS B Cells | | | Upregulated in Activated ASyS B Cells | | |
| Gene | Fold Change | padj | Gene | Fold Change | padj |
| CD24 | 1.50 | 2.87E-17 | FAM118A | 2.47 | 1.16E-06 |
| CD27 | 1.57 | 8.48E-13 | FAM177B | 2.73 | 2.05E-05 |
| CD320 | 2.21 | 7.44E-07 | FAM207A | 1.70 | 1.81E-05 |
| CD68 | 1.69 | 4.39E-03 | FCRL5 | 1.53 | 2.33E-03 |
| CD69 | 2.21 | 8.05E-44 | FDFT1 | 1.28 | 6.94E-08 |
| CD86 | 1.57 | 2.51E-06 | FGD2 | 1.27 | 5.99E-03 |
| CD99 | 1.69 | 1.22E-38 | FGR | 1.81 | 9.95E-24 |
| CDK2AP2 | 1.45 | 3.07E-09 | FKBP3 | 1.46 | 1.50E-04 |
| CFLAR | 1.56 | 5.54E-07 | FKBP5 | 10.05 | 7.31E-38 |
| CHRAC1 | 1.36 | 7.41E-03 | FOS | 2.32 | 5.78E-24 |
| CIAO2A | 1.33 | 5.47E-13 | FUCA2 | 1.50 | 3.40E-03 |
| CIAO2B | 1.29 | 3.49E-04 | GABPB1-AS1 | 1.39 | 7.34E-05 |
| CIB1 | 1.38 | 5.63E-06 | GALNTL6 | 3.47 | 1.84E-11 |
| CKLF | 1.39 | 3.09E-04 | GAPDH | 1.56 | 7.83E-57 |
| CLEC2B | 1.61 | 2.14E-06 | GATD3A | 2.29 | 5.35E-13 |
| CMTM7 | 1.69 | 8.23E-11 | GEMIN7 | 1.36 | 8.35E-05 |
| COA8 | 1.58 | 3.48E-07 | GLO1 | 1.99 | 1.19E-33 |
| COMMD8 | 1.47 | 1.38E-05 | GLUD1 | 1.41 | 2.99E-04 |
| COMTD1 | 1.76 | 4.77E-06 | GNAI2 | 1.33 | 8.77E-04 |
| COTL1 | 1.31 | 9.66E-24 | GNG2 | 1.74 | 1.62E-10 |
| COX20 | 1.46 | 1.18E-05 | GOLGA8B | 1.96 | 1.40E-11 |
| COX5A | 1.40 | 4.42E-14 | GRHPR | 2.06 | 4.87E-22 |
| CPNE5 | 1.54 | 1.49E-18 | GSN | 2.21 | 1.95E-15 |
| CPPED1 | 1.61 | 1.91E-03 | GSTO1 | 1.29 | 6.32E-03 |
| CRACR2B | 2.00 | 1.11E-09 | GTF2A2 | 1.37 | 2.11E-07 |
| CRIP1 | 2.58 | 2.35E-69 | GTF2H5 | 1.39 | 1.58E-03 |
| CRIP2 | 2.22 | 7.31E-10 | GTF3C6 | 1.36 | 5.61E-06 |
| CUX1 | 1.43 | 8.14E-03 | H2AFJ | 1.56 | 2.93E-03 |
| CXCR4 | 1.26 | 1.28E-08 | H2AFV | 1.41 | 2.63E-06 |
| CXXC1 | 1.51 | 5.97E-03 | HAGHL | 2.13 | 1.26E-19 |
| CYB5R3 | 1.68 | 4.37E-22 | HCK | 1.41 | 2.02E-06 |
| CYTH1 | 1.32 | 7.75E-05 | HDAC9 | 1.49 | 9.47E-04 |
| CYTIP | 1.58 | 4.61E-20 | HIST1H2BK | 1.46 | 1.39E-03 |
| CYTOR | 2.34 | 1.70E-08 | HMGA1 | 1.35 | 1.26E-06 |
| DAP | 1.29 | 7.00E-03 | HMOX1 | 2.01 | 1.25E-14 |
| DBI | 1.32 | 6.25E-12 | HSPA4 | 1.49 | 1.76E-06 |
| DDIT4 | 2.70 | 7.05E-64 | IFITM1 | 1.70 | 9.41E-33 |
| DDX43 | 4.14 | 6.36E-05 | IFITM2 | 1.41 | 5.81E-24 |
| DHRS4L2 | 2.18 | 4.06E-04 | IFNGR1 | 1.41 | 3.68E-04 |
| DHRS9 | 2.72 | 2.04E-08 | IFT57 | 1.77 | 3.82E-34 |
| DIAPH1 | 1.63 | 4.45E-05 | IL27RA | 1.34 | 6.07E-03 |
| DPH1 | 1.45 | 2.81E-04 | ILK | 1.33 | 4.46E-03 |
| DUSP1 | 1.74 | 1.21E-48 | IQGAP2 | 1.91 | 3.42E-05 |
| DUSP4 | 6.92 | 2.04E-04 | IRF5 | 1.50 | 6.31E-08 |
| DYNLL1 | 1.39 | 5.94E-13 | IRF7 | 1.33 | 3.25E-05 |
| DYNLRB1 | 1.29 | 1.93E-04 | ISG20 | 2.14 | 1.27E-122 |
| EBI3 | 2.24 | 2.86E-14 | ITGAE | 1.62 | 2.23E-12 |
| EMB | 1.34 | 1.93E-04 | ITGB2-AS1 | 1.45 | 5.60E-06 |
| ERAP2 | 1.73 | 2.20E-12 | ITGB7 | 1.33 | 1.47E-03 |
| ERGIC1 | 1.53 | 2.17E-09 | JOSD2 | 1.75 | 1.95E-03 |
| ERGIC3 | 1.30 | 6.33E-06 | JPT1 | 1.57 | 3.31E-10 |
| EXOSC5 | 1.41 | 6.61E-04 | JUN | 1.41 | 9.35E-22 |
| EZH2 | 3.44 | 4.19E-04 | KEAP1 | 1.61 | 3.36E-03 |
|  |  |  |  |  |  |
| Supplemental Table S7. Differentially Expressed Genes in Memory B cells in ASyS Compared to HC (continued) | | | | | |
| Upregulated in Activated ASyS B Cells | | | Upregulated in Activated ASyS B Cells | | |
| Gene | Fold Change | padj | Gene | Fold Change | padj |
| KLF2 | 1.74 | 2.87E-41 | NUDT5 | 1.46 | 2.59E-04 |
| KLF6 | 1.96 | 7.02E-61 | OSTF1 | 1.32 | 3.94E-10 |
| KLK1 | 2.48 | 8.76E-15 | P2RX5 | 2.84 | 2.61E-121 |
| KLRK1 | 10.19 | 1.33E-23 | PARP15 | 1.33 | 2.49E-04 |
| KPNA2 | 1.58 | 1.79E-06 | PARVB | 1.49 | 1.01E-06 |
| LAMTOR2 | 1.37 | 1.35E-03 | PBXIP1 | 1.36 | 6.83E-04 |
| LBR | 1.41 | 5.72E-05 | PDLIM1 | 1.39 | 5.09E-17 |
| LGALS1 | 2.04 | 2.27E-22 | PEBP4 | 4.27 | 9.95E-04 |
| LGALS9 | 1.38 | 9.73E-04 | PGLS | 1.29 | 2.90E-05 |
| LILRB4 | 11.99 | 3.57E-04 | PIGT | 1.43 | 3.85E-03 |
| LIMS1 | 1.59 | 3.40E-06 | PIH1D1 | 1.40 | 4.34E-04 |
| LIMS2 | 2.07 | 1.12E-11 | PIM1 | 1.63 | 1.65E-03 |
| LINC02384 | 2.34 | 1.47E-05 | PLAAT3 | 2.18 | 7.41E-04 |
| LINC02397 | 1.29 | 3.89E-04 | PLAC8 | 1.52 | 1.79E-18 |
| LMNA | 9.11 | 7.97E-20 | PLEK | 1.26 | 4.01E-03 |
| LPXN | 1.43 | 8.60E-09 | PLEKHJ1 | 1.38 | 1.43E-07 |
| LY6E | 1.43 | 2.86E-24 | PLPP5 | 1.41 | 5.38E-10 |
| MAGEF1 | 1.41 | 7.14E-03 | POLR2G | 1.33 | 9.48E-04 |
| MAP1LC3B | 1.27 | 9.75E-06 | POLR2J3 | 1.61 | 1.16E-06 |
| MAP3K8 | 1.70 | 2.12E-17 | POLR2L | 1.25 | 5.56E-04 |
| MAPRE2 | 1.39 | 6.44E-06 | POMP | 1.37 | 2.11E-08 |
| MARCKS | 1.37 | 1.40E-03 | PPP1R14A | 1.55 | 2.46E-08 |
| MCL1 | 1.56 | 1.42E-06 | PPP1R15A | 1.65 | 3.15E-21 |
| METTL7A | 1.37 | 2.88E-04 | PRCD | 2.33 | 4.60E-05 |
| MIR155HG | 1.94 | 4.03E-05 | PRDX1 | 1.39 | 1.44E-15 |
| MPST | 1.91 | 3.24E-11 | PRDX3 | 1.35 | 4.72E-04 |
| MRPL12 | 1.42 | 6.70E-04 | PRDX5 | 1.31 | 3.30E-08 |
| MRPL15 | 1.43 | 8.15E-03 | PRDX6 | 1.53 | 5.95E-05 |
| MRPL36 | 1.51 | 2.07E-04 | PRR5 | 2.20 | 2.93E-03 |
| MRPL40 | 1.32 | 3.96E-04 | PSMB2 | 1.34 | 2.12E-04 |
| MRPL51 | 1.32 | 6.95E-03 | PSMB4 | 1.41 | 1.73E-04 |
| MRPS6 | 1.50 | 3.76E-11 | PSMB6 | 1.28 | 3.63E-05 |
| MTHFD2 | 1.74 | 9.45E-06 | PSMD9 | 1.42 | 4.30E-06 |
| MTSS1 | 1.35 | 2.92E-07 | PTGER4 | 2.34 | 2.33E-04 |
| MX1 | 1.44 | 8.85E-09 | PTP4A2 | 1.28 | 2.74E-03 |
| MYADM | 2.32 | 1.43E-13 | PTP4A3 | 2.01 | 8.17E-10 |
| MYH9 | 1.26 | 9.40E-05 | PTPN18 | 2.11 | 1.19E-22 |
| MYL12A | 1.29 | 5.27E-32 | PYCARD | 1.38 | 3.33E-08 |
| MYO7B | 4.51 | 2.36E-16 | RAB5IF | 1.42 | 1.64E-04 |
| NABP2 | 1.68 | 7.07E-05 | RAC1 | 1.26 | 3.41E-04 |
| NARF | 1.41 | 1.72E-04 | RAC2 | 1.33 | 1.54E-40 |
| NCF2 | 1.49 | 7.48E-03 | RANBP2 | 1.46 | 2.81E-04 |
| NCOA7 | 1.44 | 1.65E-03 | RAP1B | 1.38 | 8.11E-10 |
| NDUFA12 | 1.32 | 1.18E-07 | RASGRP1 | 2.08 | 9.48E-08 |
| NDUFA8 | 1.39 | 7.17E-03 | RCSD1 | 1.40 | 5.54E-24 |
| NEAT1 | 2.13 | 2.91E-29 | REXO2 | 1.43 | 3.78E-03 |
| NKG7 | 9.50 | 6.88E-10 | RGCC | 5.73 | 4.05E-04 |
| NME1 | 1.83 | 2.67E-11 | RHOB | 3.29 | 7.02E-62 |
| NME2 | 1.28 | 9.46E-18 | RHOC | 1.56 | 6.49E-05 |
| NOP10 | 1.28 | 5.55E-05 | RNASE4 | 7.66 | 1.44E-14 |
| NPEPPS | 1.87 | 2.88E-16 | RNASE6 | 1.70 | 7.38E-09 |
| NUCB1 | 1.30 | 5.04E-03 | RNF166 | 1.82 | 1.38E-05 |
| NUDCD2 | 1.46 | 4.83E-07 | RNPEPL1 | 1.55 | 1.83E-05 |
|  | | | | | |
| Supplemental Table S7. Differentially Expressed Genes in Memory B cells in ASyS Compared to HC (continued) | | | | | |
| Upregulated in Activated ASyS B Cells | | | Upregulated in Activated ASyS B Cells | | |
| Gene | Fold Change | padj | Gene | Fold Change | padj |
| ROMO1 | 1.29 | 9.75E-06 | TKT | 1.46 | 5.54E-14 |
| RPS27L | 1.26 | 4.50E-05 | TLE1 | 2.27 | 5.23E-35 |
| RSU1 | 1.35 | 7.07E-04 | TLE4 | 1.64 | 1.90E-04 |
| S100A10 | 1.59 | 3.68E-12 | TMEM14C | 1.33 | 5.09E-05 |
| S100A11 | 2.00 | 1.46E-25 | TMEM273 | 1.58 | 1.37E-04 |
| S100A4 | 1.70 | 4.07E-20 | TMEM43 | 1.59 | 5.35E-03 |
| S100A6 | 1.40 | 7.55E-19 | TMSB4X | 1.36 | 4.83E-76 |
| SAMHD1 | 2.05 | 8.46E-06 | TNFRSF13B | 1.48 | 1.74E-12 |
| SEC11A | 1.34 | 6.51E-08 | TOR3A | 1.38 | 8.73E-06 |
| SEC61B | 1.26 | 1.04E-05 | TOX | 2.29 | 1.91E-06 |
| SERPINB1 | 1.42 | 6.00E-10 | TP53I3 | 2.58 | 1.57E-06 |
| SERPINB6 | 1.63 | 2.23E-07 | TP53INP1 | 1.93 | 6.41E-06 |
| SESN1 | 3.08 | 7.65E-10 | TPI1 | 1.35 | 2.94E-15 |
| SIGIRR | 1.27 | 3.52E-03 | TPM4 | 1.33 | 4.00E-06 |
| SIK1 | 3.79 | 1.03E-08 | TRAPPC1 | 1.27 | 2.93E-08 |
| SLC25A20 | 2.10 | 1.56E-08 | TSC22D3 | 1.91 | 1.94E-128 |
| SLC2A3 | 1.36 | 9.67E-03 | TSPO | 2.22 | 1.56E-31 |
| SLC39A4 | 1.89 | 4.35E-03 | TTC7A | 1.72 | 2.40E-04 |
| SMAP2 | 2.20 | 5.68E-40 | TUBB6 | 1.65 | 1.12E-06 |
| SMIM14 | 1.67 | 1.53E-23 | TUFM | 1.32 | 2.63E-09 |
| SMIM20 | 1.47 | 2.22E-04 | TWF2 | 1.28 | 4.30E-05 |
| SMKR1 | 2.10 | 1.22E-03 | TXN | 1.48 | 3.27E-08 |
| SMYD2 | 1.57 | 1.23E-03 | TXN2 | 1.37 | 5.19E-06 |
| SND1 | 1.40 | 3.33E-06 | TXNIP | 1.59 | 7.69E-23 |
| SNHG16 | 1.38 | 1.13E-03 | U2AF1 | 1.38 | 6.52E-04 |
| SNRNP25 | 1.47 | 2.69E-04 | UBE2A | 1.40 | 2.45E-07 |
| SNX10 | 1.34 | 6.61E-04 | UBE2N | 1.34 | 4.73E-06 |
| SNX3 | 1.35 | 9.76E-24 | UBL7 | 1.31 | 4.31E-03 |
| SNX5 | 1.32 | 1.55E-03 | UQCRQ | 1.31 | 1.95E-07 |
| SOCS1 | 2.28 | 1.78E-10 | VASP | 1.56 | 5.18E-21 |
| SP100 | 1.25 | 1.84E-09 | VDAC1 | 1.49 | 2.67E-10 |
| SPG21 | 1.39 | 1.56E-04 | VIM | 1.46 | 1.74E-36 |
| SRGAP2 | 1.94 | 3.09E-04 | VOPP1 | 1.25 | 8.57E-04 |
| SRGN | 2.08 | 3.52E-25 | WEE1 | 3.28 | 2.49E-18 |
| SRI | 1.30 | 4.14E-04 | YBX3 | 7.67 | 3.75E-46 |
| SRPK1 | 1.45 | 8.66E-03 | YIPF3 | 1.41 | 2.08E-04 |
| STAG3 | 4.43 | 6.51E-39 | YPEL3 | 1.34 | 4.87E-03 |
| STRADB | 1.67 | 2.83E-03 | YWHAH | 1.91 | 9.47E-20 |
| STXBP2 | 1.31 | 1.60E-03 | ZBTB16 | 6.32 | 5.03E-11 |
| SUB1 | 1.41 | 1.30E-18 | ZBTB32 | 1.69 | 3.26E-07 |
| SUMF2 | 1.40 | 6.77E-04 | ZDHHC12 | 1.37 | 7.29E-03 |
| SUMO1 | 1.31 | 4.44E-05 | ZFP36 | 1.43 | 4.11E-06 |
| SUPT4H1 | 1.33 | 4.63E-05 | ZFP36L2 | 1.47 | 1.88E-16 |
| SYT1 | 2.44 | 2.74E-03 |  |  |  |
| TAGLN2 | 1.48 | 7.40E-33 |  |  |  |
| TALDO1 | 1.28 | 1.87E-07 |  |  |  |
| TAX1BP3 | 3.23 | 1.40E-22 |  |  |  |
| TESC | 1.56 | 3.02E-05 |  |  |  |
| TFEB | 1.94 | 1.45E-30 |  |  |  |
| TIMM10 | 1.85 | 1.94E-16 |  |  |  |
| TIMM13 | 1.44 | 1.70E-05 |  |  |  |
| TIMP1 | 2.28 | 1.27E-06 |  |  |  |

| Supplemental Table S8. Overrepresented pathways in activated B cells. Upregulated genes with fold change increase > 1.25 and FDR q<0.01 in ASyS compared to HC were included in g:profiler analysis. | | | |
| --- | --- | --- | --- |
| GO.ID | Pathway Description | FDR Q value | Upregulated genes in pathway |
| GO:0051607 | defense response to virus | 1.60E-17 | USP18,IFI44L,IFITM1,STAT1,IFI6,RNASE6,MX1,PLSCR1,ISG15,DDIT4,  EIF2AK2,OAS1,IRF7,MX2,IFITM2,TRIM22,BST2,ISG20 |
| GO:0009615 | response to virus | 7.74E-15 | USP18,IFI44L,IFITM1,STAT1,IFI6,RNASE6,MX1,PLSCR1,ISG15,DDIT4,  EIF2AK2,OAS1,IRF7,MX2,IFITM2,TRIM22,BST2,ISG20 |
| GO:0048525 | negative regulation of viral process | 2.77E-12 | IFITM1,STAT1,MX1,PLSCR1,ISG15,EIF2AK2,OAS1,IFITM2,LY6E,BST2,  ISG20 |
| GO:0050792 | regulation of viral process | 3.40E-11 | IFITM1,STAT1,MX1,PLSCR1,ISG15,EIF2AK2,OAS1,IFITM2,TRIM22,LY6E,BST2,ISG20 |
| GO:0045071 | negative regulation of viral genome replication | 3.40E-11 | IFITM1,MX1,PLSCR1,ISG15,EIF2AK2,OAS1,IFITM2,BST2,ISG20 |
| GO:1903900 | regulation of viral life cycle | 1.71E-10 | IFITM1,MX1,PLSCR1,ISG15,EIF2AK2,OAS1,IFITM2,TRIM22,LY6E,BST2,  ISG20 |
| GO:0035456 | response to interferon-beta | 8.98E-10 | XAF1,IFITM1,STAT1,PLSCR1,OAS1,IFITM2,BST2 |
| GO:0016032 | viral process | 8.98E-10 | IFITM1,STAT1,MX1,PLSCR1,ISG15,EIF2AK2,OAS1,IRF7,IFITM2,TRIM22,  LY6E,BST2,JUN,ISG20 |
| GO:0045069 | regulation of viral genome replication | 8.98E-10 | IFITM1,MX1,PLSCR1,ISG15,EIF2AK2,OAS1,IFITM2,BST2,ISG20 |
| GO:0035455 | response to interferon-alpha | 4.51E-09 | IFITM1,EIF2AK2,OAS1,MX2,IFITM2,BST2 |
| GO:0019079 | viral genome replication | 1.56E-08 | IFITM1,MX1,PLSCR1,ISG15,EIF2AK2,OAS1,IFITM2,BST2,ISG20 |
| GO:0019058 | viral life cycle | 2.53E-08 | IFITM1,MX1,PLSCR1,ISG15,EIF2AK2,OAS1,IFITM2,TRIM22,LY6E,BST2,  ISG20 |
| GO:0034340 | response to type I interferon | 5.06E-07 | USP18,IFITM1,STAT1,MX1,ISG15,OAS1,IFITM2 |
| GO:0060337 | type I interferon-mediated signaling pathway | 8.02E-06 | USP18,IFITM1,STAT1,ISG15,OAS1,IFITM2 |
| GO:0071357 | cellular response to type I interferon | 8.38E-06 | USP18,IFITM1,STAT1,ISG15,OAS1,IFITM2 |
| GO:0140888 | interferon-mediated signaling pathway | 3.34E-05 | USP18,IFITM1,STAT1,ISG15,OAS1,IFITM2 |
| GO:0019221 | cytokine-mediated signaling pathway | 4.21E-05 | USP18,IFITM1,SOCS1,STAT1,MX1,ISG15,OAS1,IFITM2,CD24,TMSB4X |
| GO:1903901 | negative regulation of viral life cycle | 5.86E-05 | IFITM1,IFITM2,LY6E,BST2 |
| GO:0046596 | regulation of viral entry into host cell | 6.81E-04 | IFITM1,IFITM2,TRIM22,LY6E |
| GO:0140374 | antiviral innate immune response | 7.84E-04 | USP18,MX1,EIF2AK2,OAS1 |
| GO:0060339 | negative regulation of type I interferon-mediated signaling pathway | 1.03E-03 | USP18,ISG15,OAS1 |
| GO:0052372 | modulation by symbiont of entry into host | 1.07E-03 | IFITM1,IFITM2,TRIM22,LY6E |
| GO:0032606 | type I interferon production | 1.07E-03 | XAF1,STAT1,ISG15,OAS1,IRF7 |
| GO:0032479 | regulation of type I interferon production | 1.07E-03 | XAF1,STAT1,ISG15,OAS1,IRF7 |
| GO:0046597 | negative regulation of viral entry into host cell | 1.10E-03 | IFITM1,IFITM2,LY6E |
| GO:0043903 | regulation of biological process involved in symbiotic interaction | 1.49E-03 | IFITM1,IFITM2,TRIM22,LY6E |
| GO:0035458 | cellular response to interferon-beta | 1.87E-03 | STAT1,OAS1,IFITM2 |
| GO:0032481 | positive regulation of type I interferon production | 2.08E-03 | STAT1,ISG15,OAS1,IRF7 |
| GO:0002831 | regulation of response to biotic stimulus | 2.57E-03 | USP18,STAT1,PLSCR1,ISG15,EIF2AK2,OAS1,IRF7,TRIM22 |
| GO:0046718 | symbiont entry into host cell | 4.36E-03 | IFITM1,IFITM2,TRIM22,LY6E |
| GO:0002683 | negative regulation of immune system process | 5.47E-03 | USP18,SOCS1,ISG15,OAS1,DUSP1,BST2,TSC22D3 |
| GO:0044409 | symbiont entry into host | 5.57E-03 | IFITM1,IFITM2,TRIM22,LY6E |
| GO:0060338 | regulation of type I interferon-mediated signaling pathway | 5.64E-03 | USP18,ISG15,OAS1 |
| GO:0032728 | positive regulation of interferon-beta production | 6.50E-03 | ISG15,OAS1,IRF7 |
| GO:0034341 | response to type II interferon | 9.32E-03 | IFITM1,STAT1,IFITM2,BST2 |
| REAC:R-HSA-909733 | Interferon alpha/beta signaling | 3.67E-18 | USP18,XAF1,IFITM1,SOCS1,STAT1,IFI6,MX1,ISG15,OAS1,IRF7,MX2,  IFITM2,BST2,ISG20 |
| REAC:R-HSA-913531 | Interferon Signaling | 4.21E-15 | USP18,XAF1,IFITM1,SOCS1,STAT1,IFI6,MX1,ISG15,EIF2AK2,OAS1,IRF7,MX2,IFITM2,TRIM22,BST2,ISG20 |
| REAC:R-HSA-1169410 | Antiviral mechanism by IFN-stimulated genes | 2.60E-06 | USP18,STAT1,MX1,ISG15,EIF2AK2,OAS1,MX2 |
| REAC:R-HSA-1169408 | ISG15 antiviral mechanism | 2.44E-05 | USP18,STAT1,MX1,ISG15,EIF2AK2,MX2 |
| WP:WP619 | Type II interferon signaling | 2.05E-07 | SOCS1,STAT1,IFI6,ISG15,EIF2AK2,OAS1,PSMB9 |
| WP:WP4197 | Immune response to tuberculosis | 1.15E-05 | IFITM1,SOCS1,STAT1,MX1,OAS1 |
| WP:WP5115 | Network map of SASYS CoV 2 signaling pathway | 1.64E-05 | IFI44L,IFITM1,STAT1,IFI6,MX1,DDIT4,DUSP1,FOS,BST2,JUN |
| WP:WP4630 | Measles virus infection | 3.69E-05 | STAT1,MX1,EIF2AK2,OAS1,IRF7,FOS,CCND3,JUN |
| WP:WP4880 | Host pathogen interaction of human coronaviruses interferon induction | 3.98E-05 | STAT1,EIF2AK2,OAS1,FOS,JUN |
| WP:WP4877 | Host pathogen interaction of human coronaviruses MAPK signaling | 5.20E-05 | IFITM1,IFITM2,FOS,BST2,JUN |
| WP:WP3972 | PDGFR beta pathway | 5.68E-04 | STAT1,EIF2AK2,FOS,JUN |
| WP:WP4868 | Type I interferon induction and signaling during SASYS CoV 2 infection | 6.54E-04 | STAT1,EIF2AK2,OAS1,IRF7 |

| Supplemental Table S9. Detailed expression data for featured GO pathways in activated B cells for ASyS v. HC | | |
| --- | --- | --- |
| Gene | Fold Change | padj |
| GO:0051607 Defense Response to Virus  (padj 1.6E-17) | | |
| BST2 | 1.51 | 2.74E-06 |
| DDIT4 | 2.34 | 4.43E-05 |
| EIF2AK2 | 2.22 | 1.09E-05 |
| IFI44L | 7.98 | 8.31E-41 |
| IFI6 | 2.53 | 2.30E-04 |
| IFITM1 | 3.69 | 1.91E-70 |
| IFITM2 | 1.74 | 4.44E-38 |
| IRF7 | 1.92 | 1.64E-07 |
| ISG15 | 2.35 | 1.13E-03 |
| ISG20 | 1.32 | 8.84E-08 |
| MX1 | 2.42 | 1.98E-16 |
| MX2 | 1.89 | 8.19E-04 |
| OAS1 | 2.21 | 1.17E-08 |
| PLSCR1 | 2.40 | 3.03E-04 |
| RNASE6 | 2.46 | 3.50E-12 |
| STAT1 | 2.69 | 9.37E-05 |
| TRIM22 | 1.54 | 4.33E-06 |
| USP18 | 11.81 | 2.80E-03 |
| WP:WP3972 PDGFR beta pathway (padj 5.7E-4) | | |
| EIF2AK2 | 2.22 | 1.09E-05 |
| FOS | 1.64 | 8.78E-10 |
| JUN | 1.49 | 5.66E-11 |
| STAT1 | 2.69 | 9.37E-05 |
| GO:0048525 Negative Regulation of Viral Process (padj 2.77E-12) | | |
| BST2 | 1.51 | 2.74E-06 |
| EIF2AK2 | 2.22 | 1.09E-05 |
| IFITM1 | 3.69 | 1.91E-70 |
| IFITM2 | 1.74 | 4.44E-38 |
| ISG15 | 2.35 | 1.13E-03 |
| ISG20 | 1.32 | 8.84E-08 |
| LY6E | 1.54 | 3.04E-16 |
| MX1 | 2.42 | 1.98E-16 |
| OAS1 | 2.21 | 1.17E-08 |
| PLSCR1 | 2.40 | 3.03E-04 |
| STAT1 | 2.69 | 9.37E-05 |

| Supplemental Table S10. Top up- and down-regulated genes for activated B cells in ASyS compared to HC | | | | | |
| --- | --- | --- | --- | --- | --- |
| Upregulated Genes | | | Downregulated Genes | | |
|  | Fold Change | padj |  | Fold Change | padj |
| USP18 | 11.81 | 2.80E-03 | ABO | 18.06 | 1.15E-08 |
| IFI44L | 7.98 | 8.31E-41 | POLR2J2 | 5.93 | 7.51E-11 |
| CNTNAP2 | 6.89 | 5.24E-06 | SNED1 | 5.21 | 8.00E-03 |
| FAM177B | 5.23 | 1.36E-10 | FAM111B | 2.94 | 9.31E-03 |
| STAG3 | 5.21 | 4.86E-14 | SGK1 | 2.65 | 3.29E-04 |
| FKBP5 | 5.13 | 3.55E-04 | DNASE1L3 | 2.26 | 1.43E-03 |
| XAF1 | 4.15 | 6.49E-39 | BCL2A1 | 2.06 | 8.41E-04 |
| IFITM1 | 3.69 | 1.91E-70 | SNX9 | 1.97 | 5.71E-05 |
| SOCS1 | 3.35 | 7.77E-03 | NFKBID | 1.96 | 1.90E-03 |
| PDXDC1 | 2.84 | 7.95E-04 | HOPX | 1.95 | 3.49E-03 |
| STAT1 | 2.69 | 9.37E-05 | ANXA4 | 1.89 | 1.10E-15 |
| AC253572.2 | 2.66 | 2.12E-06 | IL10RA | 1.80 | 1.10E-04 |
| RHOB | 2.62 | 2.98E-13 | FCER2 | 1.65 | 4.76E-40 |
| IFI6 | 2.53 | 2.30E-04 | HIST1H2AC | 1.62 | 2.06E-06 |
| RNASE6 | 2.46 | 3.50E-12 | SBDS | 1.50 | 6.95E-03 |
| MX1 | 2.42 | 1.98E-16 | CD82 | 1.44 | 1.15E-05 |
| PLSCR1 | 2.40 | 3.03E-04 | EIF5A | 1.39 | 5.34E-07 |
| ISG15 | 2.35 | 1.13E-03 | MT-CYB | 1.39 | 6.86E-68 |
| DDIT4 | 2.34 | 4.43E-05 | TLE5 | 1.38 | 2.59E-05 |
| EPSTI1 | 2.32 | 1.07E-07 | RPS26 | 1.29 | 3.02E-59 |
| AC245014.3 | 2.30 | 4.54E-05 |  |  |  |
| SESN1 | 2.24 | 2.68E-04 |  |  |  |
| CD69 | 2.23 | 3.62E-65 |  |  |  |
| EIF2AK2 | 2.22 | 1.09E-05 |  |  |  |
| OAS1 | 2.21 | 1.17E-08 |  |  |  |

| Supplemental Table S11. Overrepresented pathways in memory B cells. Upregulated genes with fold change increase > 1.25 and FDR q<0.01 in ASyS compared to HC were included in g:profiler analysis. | | | |
| --- | --- | --- | --- |
| GO.ID | Pathway Description | FDR Q value | Upregulated genes in pathway |
| GO:0007015 | actin filament organization | 1.05E-08 | RGCC,MYO7B,ANG,RHOB,PTGER4,MYADM,GSN,CCDC88A,IQGAP2,  DIAPH1,S100A10,VASP,RHOC,ARPC5,TAGLN2,ARPC5L,HCK,PDLIM1,  PYCARD,MARCKS,TMSB4X,MTSS1,ARPC1B,RAC2,BRK1,TPM4,COTL1,  ACTG1,TWF2,ARPC4,PLEK,CAP1,RAC1 |
| GO:0032970 | regulation of actin filament-based process | 2.02E-05 | RGCC,PTGER4,MYADM,GSN,CCDC88A,IQGAP2,ABRACL,S100A10,  VASP,RHOC,HCK,AKAP13,PYCARD,TMSB4X,MTSS1,BRK1,COTL1,  ACTG1,SRI,TWF2,PLEK,MYH9,RAC1 |
| GO:0110053 | regulation of actin filament organization | 8.15E-05 | RGCC,PTGER4,MYADM,GSN,CCDC88A,S100A10,VASP,RHOC,HCK,  PYCARD,TMSB4X,MTSS1,BRK1,COTL1,ACTG1,TWF2,PLEK,RAC1 |
| GO:0032956 | regulation of actin cytoskeleton organization | 1.30E-04 | RGCC,PTGER4,MYADM,GSN,CCDC88A,IQGAP2,S100A10,VASP,RHOC,  HCK,AKAP13,PYCARD,TMSB4X,MTSS1,BRK1,COTL1,ACTG1,TWF2,  PLEK,RAC1 |
| GO:0034446 | substrate adhesion-dependent cell spreading | 2.10E-04 | MYADM,LIMS2,SRGAP2,LIMS1,S100A10,PARVB,LPXN,CIB1,ITGB7,ILK,  RAC1 |
| GO:0007229 | integrin-mediated signaling pathway | 8.00E-04 | TIMP1,LIMS2,FGR,ITGAE,LIMS1,HCK,ITGB7,ILK,NME2,PLEK,MYH9 |
| GO:1900026 | positive regulation of substrate adhesion-dependent cell spreading | 8.00E-04 | MYADM,LIMS2,LIMS1,S100A10,CIB1,ILK,RAC1 |
| GO:0051493 | regulation of cytoskeleton organization | 8.00E-04 | RGCC,PTGER4,MYADM,GSN,CCDC88A,IQGAP2,DIAPH1,S100A10,VASP,RHOC,CDK2AP2,HCK,AKAP13,MAPRE2,PYCARD,CIB1,TMSB4X,MTSS1,BRK1,COTL1,ACTG1,TWF2,PLEK,RAC1 |
| GO:1902903 | regulation of supramolecular fiber organization | 8.00E-04 | RGCC,PTGER4,MYADM,GSN,CCDC88A,S100A10,VASP,RHOC,HCK,  AKAP13,PYCARD,CIB1,TMSB4X,MTSS1,BRK1,COTL1,ACTG1,TWF2,  PLEK,RAC1 |
| GO:1903729 | regulation of plasma membrane organization | 1.10E-03 | GSN,S100A10,AHNAK,ANXA2,MYH9 |
| GO:0008154 | actin polymerization or depolymerization | 1.20E-03 | ANG,MYADM,GSN,DIAPH1,VASP,HCK,PYCARD,TMSB4X,BRK1,COTL1,  TWF2,PLEK,RAC1 |
| GO:0052372 | modulation by symbiont of entry into host | 1.20E-03 | GSN,LGALS1,IFITM1,FUCA2,LY6E,IFITM2,LGALS9,SNX3 |
| GO:0048525 | negative regulation of viral process | 1.20E-03 | GSN,ISG20,IFITM1,SRPK1,MX1,ZFP36,LY6E,IFITM2,SNX3,SP100 |
| GO:0050792 | regulation of viral process | 1.60E-03 | GSN,ISG20,LGALS1,IFITM1,KPNA2,SRPK1,MX1,ZFP36,LY6E,IFITM2,  LGALS9,SNX3,SP100 |
| GO:0043903 | regulation of biological process involved in symbiotic interaction | 2.00E-03 | GSN,LGALS1,IFITM1,FUCA2,LY6E,IFITM2,LGALS9,SNX3 |
| GO:0046597 | negative regulation of viral entry into host cell | 2.50E-03 | GSN,IFITM1,LY6E,IFITM2,SNX3 |
| GO:0051345 | positive regulation of hydrolase activity | 2.50E-03 | ANG,EZH2,TAX1BP3,GSN,RASGRP1,SRGAP2,IFT57,PPP1R15A,LIMS1,  S100A10,CD86,RHOC,MAPRE2,PYCARD,LGALS9,RSU1,DAP,CALM2,  PLEK,RPS27L |
| GO:0046596 | regulation of viral entry into host cell | 2.80E-03 | GSN,LGALS1,IFITM1,LY6E,IFITM2,LGALS9,SNX3 |
| GO:0030041 | actin filament polymerization | 2.90E-03 | ANG,MYADM,GSN,DIAPH1,VASP,HCK,PYCARD,TMSB4X,COTL1,TWF2,  RAC1 |
| GO:1902905 | positive regulation of supramolecular fiber organization | 2.90E-03 | RGCC,GSN,CCDC88A,S100A10,VASP,RHOC,HCK,PYCARD,MTSS1,  BRK1, PLEK,RAC1 |
| GO:0032231 | regulation of actin filament bundle assembly | 3.30E-03 | RGCC,PTGER4,CCDC88A,S100A10,RHOC,MTSS1,ACTG1,PLEK,  RAC1 |
| GO:0045010 | actin nucleation | 3.40E-03 | GSN,IQGAP2,ARPC5,ARPC5L,ARPC1B,BRK1,ARPC4 |
| GO:0051495 | positive regulation of cytoskeleton organization | 3.90E-03 | RGCC,GSN,CCDC88A,S100A10,VASP,RHOC,HCK,PYCARD,MTSS1,  BRK1,PLEK,RAC1 |
| GO:0019221 | cytokine-mediated signaling pathway | 4.40E-03 | LILRB4,SOCS1,EBI3,SAMHD1,IFITM1,LIMS1,CD24,IRF5,MX1,IFNGR1,  HCK,IFITM2,PYCARD,CIB1,TMSB4X,IL27RA,CASP4,ILK,SIGIRR,CXCR4,  SP100 |
| GO:0032233 | positive regulation of actin filament bundle assembly | 4.40E-03 | RGCC,CCDC88A,S100A10,RHOC,MTSS1,PLEK,RAC1 |
| GO:1900024 | regulation of substrate adhesion-dependent cell spreading | 4.40E-03 | MYADM,LIMS2,LIMS1,S100A10,CIB1,ILK,RAC1 |
| GO:0008064 | regulation of actin polymerization or depolymerization | 4.40E-03 | MYADM,GSN,VASP,HCK,PYCARD,TMSB4X,BRK1,COTL1,TWF2,PLEK |
| GO:1903900 | regulation of viral life cycle | 4.90E-03 | GSN,ISG20,LGALS1,IFITM1,KPNA2,SRPK1,MX1,LY6E,IFITM2,LGALS9,  SNX3 |
| GO:0030832 | regulation of actin filament length | 5.30E-03 | MYADM,GSN,VASP,HCK,PYCARD,TMSB4X,BRK1,COTL1,TWF2,PLEK |
| GO:0045454 | cell redox homeostasis | 5.90E-03 | PRDX6,TXN,PRDX1,TXN2,PRDX3,PRDX5 |
| GO:0043122 | regulation of canonical NF-kappaB signal transduction | 6.00E-03 | LILRB4,TLE1,LGALS1,HMOX1,S100A4,LIMS1,GAPDH,CFLAR,RHOC,  AKAP13,PYCARD,LGALS9,TMSB4X,UBE2N,ILK |
| GO:1903901 | negative regulation of viral life cycle | 6.00E-03 | GSN,IFITM1,LY6E,IFITM2,SNX3 |
| GO:0030032 | lamellipodium assembly | 6.30E-03 | CCDC88A,SRGAP2,PARVB,RAC2,BRK1,TWF2,RAC1 |
| GO:0043254 | regulation of protein-containing complex assembly | 7.10E-03 | PTGER4,MYADM,GSN,BID,VASP,RHOC,AIDA,HCK,PYCARD,RAP1B,  ZDHHC12,TMSB4X,BRK1,COTL1,SUMO1,TWF2,PLEK,RAC1 |
| GO:0031032 | actomyosin structure organization | 7.20E-03 | RGCC,PTGER4,CCDC88A,IQGAP2,S100A10,CFLAR,RHOC,AKAP13,  PDLIM1,ACTG1,MYH9,RAC1 |
| GO:0031294 | lymphocyte costimulation | 7.80E-03 | LILRB4,KLRK1,CD320,MAP3K8,CD86,CD24 |
| GO:0034314 | Arp2/3 complex-mediated actin nucleation | 7.80E-03 | IQGAP2,ARPC5,ARPC5L,ARPC1B,BRK1,ARPC4 |
| GO:0009617 | response to bacterium | 7.80E-03 | RNASE4,ANG,TFEB,FGR,RNASE6,CD68,CD86,CD24,IRF5,FUCA2,  HIST1H2BK,VIM,ZFP36,HCK,PYCARD,LGALS9,SNX3,PRDX3,CASP4,  ROMO1,SIGIRR |
| GO:0000302 | response to reactive oxygen species | 8.50E-03 | RHOB,FOS,HMOX1,COA8,TXN,JUN,PRDX1,PRDX3,PRDX5,ROMO1 |
| GO:0042743 | hydrogen peroxide metabolic process | 8.50E-03 | TXN,PRDX1,PRDX3,RAC2,PRDX5,RAC1 |
| GO:0006979 | response to oxidative stress | 9.30E-03 | RHOB,FOS,HMOX1,KEAP1,COA8,PRDX6,TXN,NCOA7,JUN,PDLIM1,  PRDX1,PRDX3,NDUFA12,PRDX5,ROMO1 |
| REAC:R-HSA-446353 | Cell-extracellular matrix interactions | 2.25E-05 | LIMS2,LIMS1,VASP,PARVB,RSU1,ILK,ACTG1 |
| REAC:R-HSA-9711123 | Cellular response to chemical stress | 2.71E-05 | SESN1,HMOX1,KEAP1,TXNIP,PRDX6,NCF2,TXN,TKT,COX20,PSMD9,  PSMB4,COX5A,PRDX1,TXN2,PRDX3,PSMB2,TALDO1,PSMB6,MAP1LC3B |
| REAC:R-HSA-6798695 | Neutrophil degranulation | 1.10E-04 | ADGRE5,GSN,S100A11,IQGAP2,FGR,CD68,CYB5R3,SERPINB6,DIAPH1,CPPED1,PRDX6,PLAC8,ARPC5,FUCA2,ANXA2,SERPINB1,DYNLL1,  PYCARD,RAP1B,ASAH1,LAMTOR2,METTL7A,SLC2A3,AP1M1,OSTF1,  COTL1,NME2,TRAPPC1,CAP1,RAC1 |
| REAC:R-HSA-195258 | RHO GTPase Effectors | 6.60E-04 | RHOB,TAX1BP3,SRGAP2,YWHAH,IQGAP2,TUBB6,DIAPH1,H2AFJ,RHOC,PPP1R14A,ARPC5,NCF2,HIST1H2BK,RANBP2,DYNLL1,ARPC1B,RAC2,  BRK1,ACTG1,ARPC4,MYH9,RAC1 |
| REAC:R-HSA-449147 | Signaling by Interleukins | 1.40E-03 | DUSP4,FOS,TIMP1,SOCS1,EBI3,PTPN18,HMOX1,MAP3K8,PIM1,CD86,  MCL1,VIM,ANXA2,PSMD9,HCK,JUN,PSMB4,RAP1B,LGALS9,IL27RA,  PSMB2,UBE2N,STXBP2,GSTO1,TALDO1,PSMB6,SIGIRR |
| REAC:R-HSA-9755511 | KEAP1-NFE2L2 pathway | 3.00E-03 | SESN1,HMOX1,KEAP1,TKT,PSMD9,PSMB4,PRDX1,PSMB2,TALDO1,  PSMB6,MAP1LC3B |
| REAC:R-HSA-9658195 | Leishmania infection | 3.10E-03 | HMOX1,FGR,GNG2,TXNIP,ARPC5,TXN,HCK,JUN,PYCARD,ARPC1B,  GNAI2,BRK1,ACTG1,ARPC4,MYH9,RAC1 |
| REAC:R-HSA-9824443 | Parasitic Infection Pathways | 3.10E-03 | HMOX1,FGR,GNG2,TXNIP,ARPC5,TXN,HCK,JUN,PYCARD,ARPC1B,  GNAI2,BRK1,ACTG1,ARPC4,MYH9,RAC1 |
| REAC:R-HSA-162906 | HIV Infection | 4.60E-03 | RANBP2,PSMD9,AP2S1,HCK,PSMB4,GTF2H5,GTF2A2,HMGA1,PSMB2,  POLR2G,SUPT4H1,AP1M1,PSMB6,CXCR4,RAC1,POLR2L |
| REAC:R-HSA-5663213 | RHO GTPases Activate WASPs and WAVEs | 7.90E-03 | ARPC5,ARPC1B,BRK1,ACTG1,ARPC4,RAC1 |
| REAC:R-HSA-3299685 | Detoxification of Reactive Oxygen Species | 7.90E-03 | PRDX6,NCF2,TXN,PRDX1,TXN2,PRDX3 |
| REAC:R-HSA-913531 | Interferon Signaling | 9.00E-03 | SOCS1,ISG20,SAMHD1,IFITM1,KPNA2,IRF5,RANBP2,MX1,IFNGR1,  IFITM2,UBE2N,IRF7,SUMO1,SP100 |

| Supplemental Table S12. Detailed expression data for featured GO pathways in memory B cells for ASyS v. HC | | | | | | | | |
| --- | --- | --- | --- | --- | --- | --- | --- | --- |
| Gene | Fold Change | | padj | Gene | Fold Change | | padj | |
| GO:0007015 Actin Filament Organization  (padj 1.6E-17) | | | | REAC:R-HAS-9711123 Cellular Response to Chemical Stress (padj 2.71E-05) | | | | |
| ACTG1 | | 1.31 | 1.75E-13 | COX20 | 1.46 | | 1.18E-05 | |
| ANG | | 4.05 | 1.10E-08 | COX5A | 1.40 | | 4.42E-14 | |
| ARPC1B | | 1.34 | 1.31E-23 | HMOX1 | 2.01 | | 1.25E-14 | |
| ARPC4 | | 1.27 | 8.76E-08 | KEAP1 | 1.61 | | 3.36E-03 | |
| ARPC5 | | 1.50 | 3.73E-23 | MAP1LC3B | 1.27 | | 9.75E-06 | |
| ARPC5L | | 1.46 | 2.42E-10 | NCF2 | 1.49 | | 7.48E-03 | |
| BRK1 | | 1.33 | 8.15E-17 | PRDX1 | 1.39 | | 1.44E-15 | |
| CAP1 | | 1.26 | 1.09E-08 | PRDX3 | 1.35 | | 4.72E-04 | |
| CCDC88A | | 1.98 | 1.24E-07 | PRDX6 | 1.53 | | 5.95E-05 | |
| COTL1 | | 1.31 | 9.66E-24 | PSMB2 | 1.34 | | 2.12E-04 | |
| DIAPH1 | | 1.63 | 4.45E-05 | PSMB4 | 1.41 | | 1.73E-04 | |
| GSN | | 2.21 | 1.95E-15 | PSMB6 | 1.28 | | 3.63E-05 | |
| HCK | | 1.41 | 2.02E-06 | PSMB9 | 1.14 | | 7.71E-03 | |
| IQGAP2 | | 1.91 | 3.42E-05 | SESN1 | 3.08 | | 7.65E-10 | |
| MARCKS | | 1.37 | 1.40E-03 | TALDO1 | 1.28 | | 1.87E-07 | |
| MTSS1 | | 1.35 | 2.92E-07 | TKT | 1.46 | | 5.54E-14 | |
| MYADM | | 2.32 | 1.43E-13 | TXN | 1.48 | | 3.27E-08 | |
| MYO7B | | 4.51 | 2.36E-16 | TXN2 | 1.37 | | 5.19E-06 | |
| PDLIM1 | | 1.39 | 5.09E-17 | TXNIP | 1.59 | | 7.69E-23 | |
| PLEK | | 1.26 | 4.01E-03 |  | |  | |  |
| PTGER4 | | 2.34 | 2.33E-04 |  | |  | |  |
| PYCARD | | 1.38 | 3.33E-08 |  | |  | |  |
| RAC1 | | 1.26 | 3.41E-04 |  | |  | |  |
| RAC2 | | 1.33 | 1.54E-40 |  | |  | |  |
| RGCC | | 5.73 | 4.05E-04 |  | |  | |  |
| RHOB | | 3.29 | 7.02E-62 |  | |  | |  |
| RHOC | | 1.56 | 6.49E-05 |  | |  | |  |
| S100A10 | | 1.59 | 3.68E-12 |  | |  | |  |
| TAGLN2 | | 1.48 | 7.40E-33 |  | |  | |  |
| TMSB4X | | 1.36 | 4.83E-76 |  | |  | |  |
| TPM4 | | 1.33 | 4.00E-06 |  | |  | |  |
| TWF2 | | 1.28 | 4.30E-05 |  | |  | |  |
| VASP | | 1.56 | 5.18E-21 |  | |  | |  |
| GO:0048525 Negative Regulation of Viral Process (padj 1.23 E-03) | | | |  | |  | |  |
| GSN | | 2.21 | 1.95E-15 |  | |  | |  |
| IFITM1 | | 1.70 | 9.41E-33 |  | |  | |  |
| IFITM2 | | 1.41 | 5.81E-24 |  | |  | |  |
| ISG20 | | 2.14 | 1.27E-122 |  | |  | |  |
| LY6E | | 1.43 | 2.86E-24 |  | |  | |  |
| MX1 | | 1.44 | 8.85E-09 |  | |  | |  |
| SNX3 | | 1.35 | 9.76E-24 |  | |  | |  |
| SP100 | | 1.25 | 1.84E-09 |  | |  | |  |
| SRPK1 | | 1.45 | 8.66E-03 |  | |  | |  |
| ZFP36 | | 1.43 | 4.11E-06 |  | |  | |  |

| Supplemental Table S13 Top up- and down-regulated genes for memory B cells in ASyS v. HC | | | | | |
| --- | --- | --- | --- | --- | --- |
| Upregulated Genes | | | Downregulated Genes | | |
|  | Fold Change | padj |  | Fold Change | padj |
| AL157402.2 | 27.08 | 2.46E-39 | TMEM204 | 10.32 | 3.35E-07 |
| LILRB4 | 11.99 | 3.57E-04 | U2AF1L5 | 4.55 | 1.20E-14 |
| KLRK1 | 10.19 | 1.33E-23 | TCF7 | 3.23 | 1.03E-05 |
| FKBP5 | 10.05 | 7.31E-38 | TEAD2 | 2.65 | 6.09E-08 |
| NKG7 | 9.50 | 6.88E-10 | CFL2 | 2.34 | 7.91E-03 |
| LMNA | 9.11 | 7.97E-20 | NFKBID | 2.24 | 5.21E-10 |
| YBX3 | 7.67 | 3.75E-46 | IRF1 | 1.90 | 7.51E-12 |
| RNASE4 | 7.66 | 1.44E-14 | FCER2 | 1.90 | 1.19E-17 |
| DUSP4 | 6.92 | 2.04E-04 | CALHM6 | 1.86 | 4.62E-34 |
| ZBTB16 | 6.32 | 5.03E-11 | TOP1MT | 1.76 | 5.40E-03 |
| RGCC | 5.73 | 4.05E-04 | VPREB3 | 1.75 | 8.89E-32 |
| MYO7B | 4.51 | 2.36E-16 | AC004687.1 | 1.67 | 2.05E-10 |
| STAG3 | 4.43 | 6.51E-39 | RELB | 1.62 | 1.27E-09 |
| PEBP4 | 4.27 | 9.95E-04 | TMEM156 | 1.55 | 4.92E-09 |
| DDX43 | 4.14 | 6.36E-05 | LY9 | 1.55 | 2.16E-03 |
| ANG | 4.05 | 1.10E-08 | LTB | 1.55 | 1.12E-75 |
| AC020916.1 | 4.01 | 2.52E-04 | NUAK2 | 1.54 | 2.19E-03 |
| SIK1 | 3.79 | 1.03E-08 | BTG2 | 1.50 | 3.16E-15 |
| GALNTL6 | 3.47 | 1.84E-11 | BIRC3 | 1.49 | 4.53E-11 |
| EZH2 | 3.44 | 4.19E-04 | SELENOM | 1.49 | 2.81E-03 |
| RHOB | 3.29 | 7.02E-62 | ADAM28 | 1.46 | 1.45E-08 |
| WEE1 | 3.28 | 2.49E-18 | RUBCNL | 1.43 | 7.77E-05 |
| TAX1BP3 | 3.23 | 1.40E-22 | PNRC1 | 1.41 | 6.19E-33 |
| SESN1 | 3.08 | 7.65E-10 | PNRC2 | 1.39 | 1.12E-04 |
| P2RX5 | 2.84 | 2.61E-121 | CD40 | 1.38 | 4.14E-08 |

| Supplemental Table S14. Raw data for Figure 4 | | | | | | |
| --- | --- | --- | --- | --- | --- | --- |
|  | CD27- | | CD27+IgM+ | | CD27+IgM- | |
|  | Healthy | ASyS | Healthy | ASyS | Healthy | ASyS |
| MitoTracker Green  (Mitochondrial bulk) | 2567.3 | 3569.1 | 3197.5 | 7557.3 | 3656.1 | 6696.7 |
|  | 4046.2 | 3942.3 | 4917.7 | 5378.2 | 5902.3 | 6005.8 |
|  | 4594.9 | 2876.5 | 4864.2 | 2880.3 | 6617.6 | 4749.9 |
|  | 4824.1 | 2818.5 | 5571.9 | 4053.5 | 6420.9 | 2988.9 |
|  | 4769.6 | 3427.6 | 6969.6 | 5858.1 | 7832.9 | 5370.6 |
|  | 1814.4 | 4701.8 | 1901.7 | 5419.2 | 2089.8 | 5197.2 |
|  |  | 1818.3 |  | 2869.2 |  | 3057.0 |
|  |  | 5066.4 |  | 5996.8 |  | 5719.8 |
|  |  | 3511.5 |  | 3912.5 |  | 4123.7 |
|  |  | 3875.8 |  | 4434.1 |  | 4248.2 |
|  |  | 2534.8 |  | 3474.6 |  | 4128.9 |
|  |  | 4680.8 |  | 5712.0 |  | 5623.1 |
| Median | 4320.5 | 3540.3 | 4890.9 | 4906.1 | 6161.6 | 4973.5 |
| IQR | 2379.1,4783.2 | 2833.0,4496.2 | 2873.6,5921.3 | 3584.1,5821.6 | 3264.5,6921.4 | 4125.0,5695.7 |
| TMRE - normalized  (Mitochondrial Membrane Potential) | 1880.6 | 2230.8 | 2833.3 | 4717.9 | 4781.356 | 6332.318 |
|  | 2078.0 | 1806.9 | 3378.0 | 2826.8 | 4041.176 | 4243.92 |
|  | 1821.5 | 2682.3 | 2765.9 | 4711.2 | 3570.661 | 6359.435 |
|  | 1863.5 | 1996.5 | 2392.8 | 2328.1 | 3789.699 | 4298.822 |
|  | 1443.7 | 1729.7 | 2199.6 | 3259.2 | 3492.669 | 3224.029 |
|  | 2495.3 | 1763.6 | 4772.7 | 2365.5 | 6171.926 | 4651.587 |
|  |  | 1179.9 |  | 1121.5 |  | 3505.455 |
|  |  | 1937.2 |  | 3130.945 |  | 4228.184 |
|  |  | 1572.7 |  | 4449.854 |  | 3813.101 |
|  |  | 2462.2 |  | 3079.222 |  | 7568.301 |
|  |  | 1401.4 |  | 3211.115 |  | 3743.824 |
|  |  | 2033.2 |  | 3279.268 |  | 5136.701 |
| Median | 1872.1 | 1872.1 | 2799.6 | 3171.0 | 3915.4 | 4271.4 |
| IQR | 1727.0,2182.3 | 1612.0,2181.4 | 2344.5,3726.7 | 2480.8,4157.2 | 3551.2,5129.0 | 3761.1,6033.4 |
| MitoSOX - normalized  (Mitochondrial Superoxide) | 273.4 | 223.1 | 350.5 | 350.5 | 438.4 | 389.6 |
|  | 117.3 | 172.0 | 190.1 | 190.1 | 218.4 | 306.4 |
|  | 102.3 | 210.6 | 188.1 | 188.1 | 177.0 | 357.6 |
|  | 96.4 | 201.4 | 134.4 | 134.4 | 164.7 | 411.3 |
|  | 112.1 | 231.8 | 144.7 | 144.7 | 142.9 | 389.2 |
|  | 642. 9 | 130.2 | 1106.7 | 1106.7 | 1281.5 | 644.4 |
|  |  | 155.5 |  | 205.2 |  | 289.1 |
|  |  | 105.5 |  | 149.8 |  | 252.8 |
|  |  | 185.3 |  | 250.8 |  | 303.5 |
|  |  | 165.1 |  | 222.9 |  | 293.8 |
|  |  | 292.9 |  | 350.7 |  | 467.3 |
|  |  | 139.9 |  | 203.6 |  | 291.9 |
| Median | 114.7 | 220.0 | 189.1 | 249.1 | 197.7 | 332.0 |
| IQR | 100.8,365.7 | 178.7,143.8 | 142.2,539.6 | 209.1,383.0 | 159.2,649.2 | 292.4,405.9 |
| DCFDA – normalized (Reactive Oxygen Species) | 1755.6 | 4655.5 | 1677.5 | 2822.8 | 1365.6 | 3259.3 |
|  | 3365.6 | 2953.2 | 3155.9 | 2520.3 | 2691.4 | 2137.0 |
|  | 2601.4 | 5181.6 | 2798.6 | 7944.3 | 1697.4 | 4224.0 |
|  | 2438.8 | 3980.5 | 2582.4 | 3573.3 | 2043.7 | 4010.6 |
|  | 4156.6 | 6550.1 | 3673.0 | 5006.1 | 2945.0 | 4408.8 |
|  | 4350.7 | 5772.3 | 4973.1 | 6865.7 | 3814.9 | 4103.5 |
|  |  | 4690.7 |  | 3768.2 |  | 3571.9 |
|  |  | 2383.7 |  | 2093.1 |  | 2042.3 |
|  |  | 5662.5 |  | 7166.5 |  | 7901.5 |
|  |  | 3566.8 |  | 4241.2 |  | 3027.7 |
|  |  | 5962.3 |  | 4769.7 |  | 3589.3 |
|  |  | 4413.2 |  | 4430.8 |  | 2831.7 |
| Median | 2983.5 | 4673.1 | 2977.3 | 4336.0 | 2367.5 | 3580.6 |
| IQR | 2268.0,4205.1 | 3670.2,5744.8 | 2356.1,3998.0 | 3010.4,6400.8 | 1614.4,3162.5 | 2880.7,4193.8 |

| Supplemental Table S15. Genes with increased frequency of expression for activated and memory subsets in ASyS versus HC. | | | | | | |
| --- | --- | --- | --- | --- | --- | --- |
| Gene | Activated | | | Memory | | |
|  | % increased expression in ASYS | Fold Change | padj | % increased expression in ASYS | Fold Change | padj |
| IFI44L | 25.9% | 7.98 | 8.31E-41 | n.s. | n.s. | n.s. |
| STAG3 | 13.3% | 5.20 | 4.86E-14 | 17.5% | 4.43 | 6.51E-39 |
| FAM177B | 10.4% | 5.22 | 1.36E-10 | 4.9% | 2.73 | 2.04E-5 |
| YBX3 | n.s. | n.s. | n.s. | 18.3% | 7.67 | 3.75E-46 |
| FKBP5 | 6.1% | 5.13 | 0.00035 | 13.7% | 10.05 | 7.30E-38 |
| AL157402.2 | n.s. | n.s. | n.s. | 12.8% | 27.08 | 2.46E-39 |
| TAX1BP3 | n.s. | n.s. | n.s. | 12.8% | 3.23 | 1.40E-22 |
| EBI3 | n.s. | n.s. | n.s. | 11.0% | 2.24 | 2.86E-14 |
| MPST | n.s. | n.s. | n.s. | 10.3% | 1.91 | 3.24E-11 |
| WEE1 | n.s. | n.s. | n.s. | 10.1% | 3.27 | 2.49E-18 |
| MYADM | n.s. | n.s. | n.s. | 10.1% | 2.32 | 1.43E-13 |
| n.s. = not significant | | | | | | |

| Supplemental Table 16. Donor Level Frequency of Candidate Gene Expression | | | | | | | | | | | | | | | | | | |
| --- | --- | --- | --- | --- | --- | --- | --- | --- | --- | --- | --- | --- | --- | --- | --- | --- | --- | --- |
| Activated B cells† | | | | | | | | | | | | | | | | | | |
| Partici-pant | FAM177B  (p=0.06) | | FKBP5  (p=0.14) | | IFI44L  (p-0.09) | | STAG3  (p=0.43) | |  | | | | | | | | | |
|  | HC | ASyS | HC | ASyS | HC | ASyS | HC | ASyS |  |  |  |  |  |  |  |  |  |  |
| 1 | 1.9% | 22.7% | 3.4% | 25.4% | 2.6% | 1.6% | 8.6% | 34.6% |  |  |  |  |  |  |  |  |  |  |
| 2 | 7.1% | 24.6% | 0.0% | 21.2% | 7.1% | 7.1% | 14.3% | 39.4% |  |  |  |  |  |  |  |  |  |  |
| 3 | 11.8% | 14.1% | 0.0% | 15.2% | 23.5% | 12.8% | 11.8% | 40.0% |  |  |  |  |  |  |  |  |  |  |
| 4 | 3.4% | 8.4% | 1.3% | 4.0% | 5.7% | 28.8% | 2.9% | 5.9% |  |  |  |  |  |  |  |  |  |  |
| 5 | 3.8% | 17.6% | 3.8% | 2.0% | 7.7% | 17.6% | 15.4% | 15.7% |  |  |  |  |  |  |  |  |  |  |
| 7 |  | 5.9% |  | 2.0% |  | 51.0% |  | 11.8% |  |  |  |  |  |  |  |  |  |  |
| 8 |  | 8.4% |  | 0.5% |  | 49.0% |  | 5.8% |  |  |  |  |  |  |  |  |  |  |
| 9 |  | 17.4% |  | 2.2% |  | 49.6% |  | 11.5% |  |  |  |  |  |  |  |  |  |  |
| 10 |  | 2.0% |  | 2.0% |  | 62.7% |  | 11.8% |  |  |  |  |  |  |  |  |  |  |
| Memory B cells‡ | | | | | | | | | | | | | | | | | | |
| Partici-pant | AL167402.2  (p=0.28) | | EBI3  (p=0.30) | | FKBP5  (p=0.04) | | MPST  (p=0.51) | | MYADM  (p=0.01) | | STAG3  (p=0.04) | | TAX1BP3  (p=0.11) | | WEE1  (p=0.08) | | YBX3  (p=0.30) | |
| 1 | 0.6% | 1.1% | 7.1% | 8.5% | 0.0% | 26.6% | 8.6% | 18.6% | 2.9% | 12.8% | 1.1% | 37.8% | 2.9% | 18.6% | 2.0% | 11.2% | 6.9% | 33.5% |
| 2 | 0.0% | 30.5% | 0.0% | 31.9% | 3.6% | 18.9% | 11.8% | 29.2% | 0.0% | 19.1% | 0.0% | 25.5% | 5.9% | 28.8% | 5.9% | 22.6% | 0.0% | 38.6% |
| 3 | 1.8% | 0.8% | 9.4% | 15.5% | 1.0% | 12.4% | 10.8% | 10.8% | 10.3% | 17.0% | 9.9% | 23.2% | 7.6% | 12.9% | 4.5% | 6.9% | 11.2% | 8.7% |
| 4 | 0.2% | 1.1% | 13.5% | 12.8% | 3.2% | 25.5% | 9.2% | 13.8% | 10.8% | 21.3% | 9.0% | 37.2% | 7.7% | 14.9% | 4.7% | 17.0% | 3.8% | 38.3% |
| 5 | 0.6% | 0.0% | 4.5% | 11.1% | 0.9% | 11.1% | 9.7% | 5.6% | 4.9% | 0.0% | 6.1% | 5.6% | 9.4% | 5.6% | 4.9% | 5.6% | 4.9% | 11.1% |
| 6 |  | 0.0% |  | 4.3% |  | 2.9% |  | 10.7% |  | 18.6% |  | 12.1% |  | 9.3% |  | 4.3% |  | 3.6% |
| 7 |  | 1.0% |  | 4.0% |  | 3.0% |  | 8.9% |  | 12.9% |  | 15.8% |  | 8.9% |  | 4.0% |  | 2.0% |
| 8 |  | 2.8% |  | 8.3% |  | 2.8% |  | 11.1% |  | 13.9% |  | 0.0% |  | 5.6% |  | 8.3% |  | 2.8% |
| 9 |  | 1.2% |  | 15.3% |  | 3.5% |  | 15.3% |  | 22.4% |  | 14.1% |  | 11.8% |  | 8.2% |  | 15.3% |
| †Participant 4 excluded as only eight activated B cells were identified  ‡Participant 10 was excluded as only one memory B cell was identified | | | | | | | | | | | | | | | | | | |

| Supplemental Table S17. Differentially Expressed Genes in FKBP5+ compared to FKBP5- Memory B cells | | | | | |
| --- | --- | --- | --- | --- | --- |
| Upregulated in FKBP+ memory B Cells | | | Downregulated in FKBP5+ Memory B Cells | | |
| Gene | Fold Change | padj | Gene | Fold Change | padj |
| ADGRE5 | 1.58 | 2.30E-06 | VPREB3 | 1.76988808 | 3.56E-03 |
| AHNAK | 1.48 | 4.99E-04 |  |  |  |
| AL157402.2 | 2.55 | 1.04E-08 |  |  |  |
| ANXA2 | 1.48 | 1.72E-04 |  |  |  |
| ARPC5 | 1.34 | 3.38E-03 |  |  |  |
| CAP1 | 1.30 | 6.43E-03 |  |  |  |
| CCDC167 | 1.94 | 1.48E-05 |  |  |  |
| CCND3 | 1.60 | 1.64E-16 |  |  |  |
| CD27 | 1.41 | 6.60E-04 |  |  |  |
| CD99 | 1.65 | 4.44E-13 |  |  |  |
| DDIT4 | 1.59 | 2.53E-11 |  |  |  |
| DLST | 1.70 | 8.09E-03 |  |  |  |
| DUSP1 | 1.24 | 2.27E-03 |  |  |  |
| DYNLL1 | 1.44 | 3.07E-04 |  |  |  |
| DYNLRB1 | 1.46 | 4.52E-03 |  |  |  |
| EBI3 | 2.33 | 7.43E-11 |  |  |  |
| GLO1 | 1.53 | 7.71E-08 |  |  |  |
| GLUL | 2.13 | 3.99E-08 |  |  |  |
| GNG2 | 1.79 | 7.80E-07 |  |  |  |
| GRAMD1C | 1.97 | 2.51E-03 |  |  |  |
| GSN | 2.04 | 4.54E-06 |  |  |  |
| HMOX1 | 1.63 | 1.09E-03 |  |  |  |
| ISG20 | 1.47 | 1.05E-15 |  |  |  |
| ITGB2-AS1 | 1.66 | 7.43E-03 |  |  |  |
| JPT1 | 1.49 | 8.00E-03 |  |  |  |
| KLF2 | 1.49 | 1.51E-07 |  |  |  |
| KLF6 | 1.46 | 2.67E-06 |  |  |  |
| LGMN | 2.90 | 1.59E-04 |  |  |  |
| LIMS2 | 1.76 | 2.11E-04 |  |  |  |
| LRRFIP1 | 1.39 | 1.65E-03 |  |  |  |
| MPST | 1.91 | 7.43E-04 |  |  |  |
| MVD | 1.96 | 8.70E-05 |  |  |  |
| MYADM | 2.51 | 7.09E-06 |  |  |  |
| NEAT1 | 1.85 | 1.81E-12 |  |  |  |
| P2RX5 | 1.52 | 1.41E-10 |  |  |  |
| PBXIP1 | 1.78 | 4.18E-07 |  |  |  |
| PRDX5 | 1.29 | 3.66E-03 |  |  |  |
| RHOB | 1.62 | 7.53E-06 |  |  |  |
| ROMO1 | 1.38 | 3.18E-04 |  |  |  |
| S100A10 | 1.69 | 2.02E-07 |  |  |  |
| S100A11 | 1.83 | 1.62E-10 |  |  |  |
| SERPINB1 | 1.29 | 4.83E-04 |  |  |  |
| SMAP2 | 1.74 | 6.33E-09 |  |  |  |
| SSPN | 1.68 | 1.36E-04 |  |  |  |
| STAG3 | 1.85 | 5.55E-07 |  |  |  |
| TAX1BP3 | 2.02 | 2.38E-05 |  |  |  |
| THEM4 | 1.56 | 1.00E-03 |  |  |  |
| TKT | 1.53 | 9.66E-05 |  |  |  |
| TLE1 | 1.58 | 1.75E-04 |  |  |  |
| TP53INP1 | 1.69 | 1.67E-03 |  |  |  |
| TSPO | 1.63 | 1.68E-05 |  |  |  |
| UBE2N | 1.49 | 1.35E-04 |  |  |  |
| YBX3 | 2.49 | 1.17E-10 |  |  |  |
| YWHAH | 1.63 | 2.17E-05 |  |  |  |
| ZFP36L2 | 1.45 | 2.67E-10 |  |  |  |
|  |  |  |  |  |  |

| Supplemental Table S18. Transcriptional changes for genes identified by STRING analysis in FKBP5+ memory B cells compared to FKBP5-Memory B cells | | | |
| --- | --- | --- | --- |
| Gene | Protein name | Fold Change | padj |
| Plasma Membrane Organization/Raft Cluster | | | |
| AHNAK | Neuroblast differentiation-associated protein AHNAK | 1.48 | 4.99E-04 |
| ANXA2 | Annexin A2 | 1.48 | 1.72E-04 |
| GSN | Gelsolin | 2.04 | 4.54E-06 |
| MYADM | Myeloid Associated Differentiation Marker | 2.51 | 3.99E-08 |
| S100A11 | S100 Calcium Binding Protein A11 | 1.83 | 1.62E-10 |
| S100A10 | S100 Calcium Binding Protein A10 | 1.69 | 2.02E-07 |
| TSPO | Translocator Protein | 1.63 | 1.68E-05 |
| Cellular Stress Cluster | | | |
| DDIT4 | DNA-Damage-Inducible Transcript 4 (DDIT4) or Regulated in Development and DNA Damage Response 1 (REDD1) | 1.59 | 2.52E-11 |
| FKBP5 | FK506 Binding Protein 5 or FK506 Binding Protein 51 | Not applicable | Not applicable |
| KLF2 | Krüppel-like Factor 2 | 1.49 | 1.51E-07 |
| KLF6 | Krüppel-like Factor 2 | 1.46 | 2.67E-10 |
| P2RX5 | P2X purinoceptor 5 | 1.52 | 1.41E-10 |
| RHOB | Ras homolog gene family, member B | 1.62 | 7.53E-06 |
| TAX1BP3 | Tax1-binding protein 3 | 2.02 | 2.38E-05 |
| TP53INP1 | Tumor protein p53-inducible nuclear protein 1 | 1.69 | 1.67E-03 |
| YWHAH | 14-3-3 protein eta | 1.63 | 2.17E-05 |
| ZFP36L2 | ZFP36 ring finger protein like 2 | 1.45 | 2.67E-10 |
| Cellular Metabolism Cluster | | | |
| DLST | Dihydrolipoamide S-Succinyltransferase | 1.70 | 8.09E-03 |
| GLO1 | Glyoxalase I | 1.53 | 7.71E-08 |
| GLUL | Glutamate Ammonia Ligase | 2.13 | 3.99E-08 |
| TKT | Transketolase | 1.53 | 9.66E-05 |

| Supplemental Table 19. Differences in healthy (HC) versus antisynthetase syndrome (ASyS) memory B cell expression of FK506 targets and associated regulator proteins | | | | | | | | | | | | | |
| --- | --- | --- | --- | --- | --- | --- | --- | --- | --- | --- | --- | --- | --- |
| Fold change difference in expression levels for HC vs. ASyS Memory B Cells | | | | | | | | | | | | | |
| Gene | Fold Change | | padj | | | |  | | | | | | |
| FKBP1A | 1.21 | | 2.52E-04 | | | |  |  |  |  |  |  |  |
| FBKP3 | 1.46 | | 1.50E-04 | | | |  | | | | | | |
| CCND3 | 2.60 | | 1.33E-102 | | | |  | | | | | | |
| PARK7 | -0.25 | | 1.4E-04 | | | |  | | | | | | |
| Frequency of expression in memory B cells | | | | | | | | | | | | | |
| Participant | FKBP1A  (MW p=0.11 | | | FKBP3  (MW p=0.02) | | | | CCND3  (MW p=0.002) | | | PARK7  (MW p=0.36) | | |
|  | HC | ASyS | | | HC | ASyS | | | HC | ASyS | | HC | ASyS |
| 1 | 12.9% | 19.4% | | | 12.9% | 19.4% | | | 43.7% | 55.6% | | 60.3% | 86.1% |
| 2 | 17.6% | 24.7% | | | 17.6^ | 24.7% | | | 23.5% | 67.1% | | 47.1% | 57.6% |
| 3 | 15.2%% | 51.0% | | | 15.2% | 27.7% | | | 35.0% | 86.8% | | 69.5% | 62.2% |
| 4 | 50.5% | 32.5% | | | 20.8% | 32.2% | | | 37.5% | 74.8% | | 59.5% | 76.9% |
| 5 | 23.3% | 22.9% | | | 23.3% | 22.9% | | | 35.6% | 71.8% | | 70.2% | 69.9% |
| 6 |  | 31.9% | | |  | 31.9% | | |  | 84.0% | |  | 69.1% |
| 7 |  | 19.2 | | |  | 19.3% | | |  | 55.7% | |  | 63.4% |
| 8 |  | 50.0% | | |  | 38.9% | | |  | 55.6% | |  | 72.2% |
| 9 |  | 60.3% | | |  | 21.8% | | |  | 38.6% | |  | 54.5% |
| ‡ASyS Participant 10 was excluded as only one memory B cell was identified  MW=mann whitney comparison | | | | | | | | | | | | | |

| Supplemental Table S20. Differentially Expressed Genes in MYADM+ versus MYADM- Memory B cells | | | | | |
| --- | --- | --- | --- | --- | --- |
| Upregulated in MYADM+ B Cells | | | Downregulated in MYADM+ B Cells | | |
| Gene | Fold Change | padj | Gene | Fold Change | padj |
| ABRACL | 1.64 | 6.17E-08 | LINC01857 | 1.92 | 3.22E-07 |
| ACAA2 | 1.50 | 3.52E-04 | SELENOM | 2.85 | 1.03E-06 |
| ACP5 | 1.37 | 1.01E-06 | VPREB3 | 1.87 | 2.60E-08 |
| ADAM15 | 2.00 | 7.41E-08 |  |  |  |
| ADD3 | 1.32 | 2.89E-03 |  |  |  |
| ADGRE5 | 2.12 | 1.60E-20 |  |  |  |
| ADK | 1.59 | 7.50E-10 |  |  |  |
| AHNAK | 1.40 | 2.95E-08 |  |  |  |
| ALDH16A1 | 1.45 | 3.39E-04 |  |  |  |
| ANXA2 | 1.60 | 3.25E-15 |  |  |  |
| ANXA4 | 1.62 | 3.29E-16 |  |  |  |
| AP1S2 | 1.53 | 4.92E-07 |  |  |  |
| APBB1IP | 1.32 | 5.14E-05 |  |  |  |
| ARF5 | 1.30 | 2.68E-03 |  |  |  |
| ARHGAP30 | 1.32 | 2.02E-03 |  |  |  |
| ARPC4 | 1.29 | 2.35E-04 |  |  |  |
| ARPC5 | 1.42 | 2.29E-10 |  |  |  |
| ARRB2 | 1.50 | 1.65E-04 |  |  |  |
| ASAH1 | 1.40 | 7.88E-03 |  |  |  |
| ATP2B1 | 1.37 | 5.15E-05 |  |  |  |
| ATP5F1C | 1.29 | 2.83E-04 |  |  |  |
| ATP5PF | 1.27 | 8.78E-04 |  |  |  |
| BIN2 | 1.40 | 4.89E-05 |  |  |  |
| C4orf3 | 1.33 | 4.20E-04 |  |  |  |
| C4orf48 | 1.59 | 5.34E-09 |  |  |  |
| CA5B | 1.57 | 2.54E-03 |  |  |  |
| CALM3 | 1.35 | 1.08E-03 |  |  |  |
| CAP1 | 1.30 | 5.20E-06 |  |  |  |
| CAPN1 | 1.58 | 3.98E-06 |  |  |  |
| CAPZB | 1.51 | 2.22E-21 |  |  |  |
| CASP8 | 1.42 | 9.62E-05 |  |  |  |
| CCDC167 | 1.99 | 1.18E-08 |  |  |  |
| CD151 | 2.29 | 2.08E-11 |  |  |  |
| CD99 | 1.54 | 2.61E-19 |  |  |  |
| CDC42 | 1.34 | 9.32E-08 |  |  |  |
| CDK2AP2 | 1.35 | 3.49E-05 |  |  |  |
| CIB1 | 1.55 | 3.44E-07 |  |  |  |
| CLEC2D | 1.55 | 3.61E-05 |  |  |  |
| CLTA | 1.30 | 7.87E-03 |  |  |  |
| COX5A | 1.32 | 3.19E-04 |  |  |  |
| CPNE5 | 1.49 | 4.97E-11 |  |  |  |
| CRIP1 | 2.27 | 1.73E-65 |  |  |  |
| CRIP2 | 2.81 | 6.50E-21 |  |  |  |
| CXCR3 | 2.16 | 1.79E-09 |  |  |  |
| CYTH1 | 1.29 | 1.17E-03 |  |  |  |
| CYTIP | 1.32 | 3.30E-03 |  |  |  |
| DAPP1 | 1.43 | 7.65E-03 |  |  |  |
| DBI | 1.29 | 6.52E-04 |  |  |  |
| DBNL | 1.26 | 7.70E-03 |  |  |  |
| DHRS9 | 3.71 | 7.04E-17 |  |  |  |
| DUS2 | 1.43 | 4.03E-05 |  |  |  |
| DYNLL1 | 1.55 | 1.78E-12 |  |  |  |
| DYNLRB1 | 1.38 | 8.09E-04 |  |  |  |
| EBI3 | 2.72 | 1.53E-28 |  |  |  |
| ELOVL5 | 1.31 | 1.48E-03 |  |  |  |
| FGD2 | 1.40 | 1.17E-03 |  |  |  |
| FGR | 1.91 | 1.56E-25 |  |  |  |
| Supplemental Table S20. Differentially Expressed Genes in MYADM+ versus MYADM- Memory B cells (cont) | | | | | |
| Upregulated in MYADM+ B Cells | | | Upregulated in MYADM+ B Cells | | |
| Gene | Fold Change | padj | Gene | Fold Change | padj |
| FIBP | 1.33 | 4.73E-03 | OAS1 | 1.43 | 8.50E-07 |
| FLNA | 1.42 | 1.55E-03 | OPTN | 1.42 | 2.36E-03 |
| FNBP1 | 1.33 | 1.25E-03 | OTUB1 | 1.29 | 1.75E-03 |
| FOS | 1.80 | 7.26E-03 | PABPC4 | 1.45 | 1.10E-03 |
| GABARAPL2 | 1.37 | 5.23E-08 | PBXIP1 | 1.61 | 9.47E-08 |
| GDI2 | 1.25 | 2.85E-04 | PDCD6IP | 1.39 | 6.76E-05 |
| GLUL | 2.79 | 4.04E-25 | PDLIM1 | 1.39 | 1.09E-10 |
| GNB2 | 1.32 | 4.34E-03 | PFKL | 1.41 | 1.10E-04 |
| GNG2 | 1.44 | 1.61E-03 | PIH1D1 | 1.69 | 5.86E-08 |
| GNG5 | 1.26 | 9.99E-04 | PLEK | 1.39 | 5.54E-07 |
| GRAMD1C | 2.05 | 1.72E-09 | PMVK | 1.56 | 6.72E-03 |
| GRHPR | 1.60 | 5.71E-07 | POMP | 1.34 | 4.09E-05 |
| GSN | 2.55 | 7.01E-24 | PPM1G | 1.38 | 2.26E-05 |
| GSTK1 | 1.34 | 1.38E-05 | PPM1M | 1.54 | 3.15E-06 |
| H2AFV | 1.38 | 5.63E-04 | PPP1CA | 1.27 | 1.44E-07 |
| H2AFY | 1.41 | 5.33E-04 | PPP1R14A | 1.94 | 1.69E-18 |
| HCK | 1.50 | 1.84E-06 | PPP1R15A | 1.42 | 1.14E-05 |
| HCST | 1.70 | 1.98E-06 | PRDX5 | 1.29 | 2.20E-03 |
| HIST1H2BK | 1.48 | 2.72E-03 | PTPN1 | 1.29 | 6.01E-03 |
| HMOX1 | 1.61 | 7.07E-04 | PYCARD | 1.37 | 1.80E-04 |
| HMOX2 | 1.45 | 7.14E-05 | RASGRP2 | 1.39 | 2.10E-07 |
| HOPX | 2.17 | 9.99E-18 | REEP5 | 1.41 | 2.84E-07 |
| HRK | 3.00 | 8.21E-15 | RHOB | 1.50 | 6.89E-04 |
| IDS | 1.41 | 3.74E-03 | RHOC | 1.65 | 2.52E-03 |
| IFI30 | 1.62 | 2.96E-14 | RNF126 | 1.35 | 8.93E-04 |
| ILK | 1.49 | 1.09E-05 | RNPEPL1 | 1.51 | 8.47E-03 |
| ISG20 | 1.31 | 1.29E-08 | S100A10 | 2.19 | 1.79E-33 |
| ITGA4 | 1.43 | 7.02E-04 | S100A11 | 1.99 | 1.17E-19 |
| ITGB2 | 1.67 | 2.01E-16 | S1PR4 | 1.46 | 6.10E-04 |
| ITGB2-AS1 | 1.91 | 1.22E-13 | SCIMP | 1.33 | 1.64E-04 |
| ITGB7 | 1.74 | 9.25E-16 | SCML4 | 1.66 | 5.08E-05 |
| JPT1 | 1.68 | 9.86E-11 | SEC11A | 1.36 | 1.01E-04 |
| KLF2 | 1.30 | 3.75E-05 | SEC61B | 1.40 | 3.06E-07 |
| KLF3 | 1.89 | 1.65E-12 | SERPINB1 | 1.68 | 5.96E-19 |
| KLF6 | 1.43 | 1.67E-07 | SERPINB6 | 1.61 | 3.30E-06 |
| KYNU | 1.68 | 1.45E-10 | SHKBP1 | 1.35 | 7.43E-06 |
| LAMTOR2 | 1.46 | 2.08E-04 | SKAP2 | 1.32 | 1.12E-03 |
| LDHA | 1.35 | 3.20E-06 | SLC44A2 | 1.32 | 6.81E-03 |
| LGMN | 2.90 | 3.16E-09 | SND1 | 1.38 | 1.03E-03 |
| LIMS1 | 1.56 | 2.94E-04 | SNRPD3 | 1.27 | 7.35E-03 |
| LIMS2 | 1.82 | 2.64E-11 | SPPL2A | 1.48 | 2.70E-03 |
| LITAF | 1.42 | 2.40E-05 | SRGN | 1.49 | 9.39E-04 |
| LRMP | 1.65 | 9.73E-12 | SSPN | 1.69 | 1.38E-07 |
| LRRFIP1 | 1.46 | 2.15E-08 | SUB1 | 1.61 | 1.85E-20 |
| MAP7D3 | 1.52 | 4.00E-05 | SUMO1 | 1.45 | 6.34E-06 |
| MCL1 | 1.53 | 3.09E-04 | TAX1BP3 | 1.74 | 5.06E-04 |
| MICU2 | 1.51 | 2.63E-03 | TCIRG1 | 1.43 | 2.25E-04 |
| MIF4GD | 1.42 | 2.89E-04 | THEM4 | 1.57 | 5.46E-05 |
| MPST | 1.51 | 7.11E-04 | TIMM10 | 1.39 | 1.12E-03 |
| MTHFD1 | 1.49 | 3.55E-03 | TKT | 1.73 | 2.11E-17 |
| MTSS1 | 1.78 | 3.10E-22 | TLE1 | 1.33 | 3.23E-03 |
| MYO1G | 1.37 | 3.45E-06 | TLE5 | 1.45 | 6.19E-10 |
| NAA38 | 1.33 | 4.67E-03 | TLN1 | 1.55 | 1.06E-06 |
| NAA50 | 1.50 | 1.87E-03 | TMBIM1 | 1.38 | 5.47E-04 |
| NDUFB2 | 1.36 | 5.56E-08 | TMED3 | 1.37 | 4.22E-03 |
| NEAT1 | 1.79 | 1.95E-15 | TMEM147 | 1.37 | 2.76E-03 |
| NOP10 | 1.29 | 2.68E-03 | TOR3A | 1.38 | 6.15E-03 |
|  |  |  |  |  |  |
|  |  |  |  |  |  |
| Supplemental Table S20. Differentially Expressed Genes in MYADM+ versus MYADM- Memory B cells (cont) | | | | | |
| Upregulated in MYADM+ B Cells | | | Upregulated in MYADM+ B Cells | | |
| Gene | Fold Change | padj | Gene | Fold Change | padj |
| TPI1 | 1.26 | 3.89E-04 | VAMP5 | 1.58 | 5.36E-04 |
| TSPO | 1.43 | 1.97E-04 | VDAC1 | 1.45 | 1.78E-07 |
| TUBA1A | 1.51 | 4.39E-07 | VRK3 | 1.40 | 6.57E-03 |
| TUBA4A | 1.34 | 5.28E-04 | VSIR | 1.82 | 5.88E-06 |
| TUBB6 | 2.35 | 1.83E-17 | YBX3 | 1.79 | 1.51E-07 |
| TUSC2 | 1.45 | 9.37E-05 | YIF1A | 1.49 | 4.60E-04 |
| TWF2 | 1.50 | 8.73E-10 | YWHAH | 1.76 | 1.78E-12 |
| UBE2N | 1.34 | 1.31E-03 | ZYX | 1.48 | 4.68E-03 |
| UVRAG | 1.42 | 2.97E-07 |  |  |  |

| Supplemental Table S21. Overrepresented pathways in MYADM+ memory B cells. Upregulated genes with fold change increase > 1.25 and FDR q<0.01 were included in g:profiler analysis. | | | |
| --- | --- | --- | --- |
| GO.ID | Pathway Description | padj | Upregulated genes in pathway |
| GO:0007229 | integrin-mediated signaling pathway | 2.42E-09 | ADAM15,FGR,LIMS2,ITGB7,ITGB2,LIMS1,TLN1,HCK,ILK,ZYX,ITGA4,FLNA,PLEK,CDC42 |
| GO:0007015 | actin filament organization | 8.81E-06 | GSN,S100A10,MTSS1,RHOC,CAPZB,HCK,TWF2,RHOB,ZYX,FLNA,ARPC5,PDLIM1,PLEK,MYO1G,PYCARD,CDC42,ADD3,CAP1,ARPC4 |
| GO:1900026 | positive regulation of substrate adhesion-dependent cell spreading | 2.15E-05 | S100A10,LIMS2,LIMS1,CIB1,ILK,FLNA,CDC42 |
| GO:0034446 | substrate adhesion-dependent cell spreading | 4.59E-05 | S100A10,LIMS2,ITGB7,LIMS1,CIB1,ILK,ITGA4,FLNA,CDC42 |
| GO:0110053 | regulation of actin filament organization | 9.26E-05 | GSN,S100A10,MTSS1,RHOC,CAPZB,HCK,TWF2,FLNA,ARPC5,PLEK,PYCARD,CDC42,ADD3 |
| GO:0043122 | regulation of canonical NF-kappaB signal transduction | 1.07E-04 | RHOC,HMOX1,LIMS1,ARRB2,ILK,FLNA,OPTN,CASP8,LITAF,PYCARD,UBE2N,TLE1,SLC44A2 |
| GO:1900024 | regulation of substrate adhesion-dependent cell spreading | 1.32E-04 | S100A10,LIMS2,LIMS1,CIB1,ILK,FLNA,CDC42 |
| GO:0031589 | cell-substrate adhesion | 2.23E-04 | S100A10,ADAM15,LIMS2,ITGB7,ITGB2,LIMS1,TLN1,CIB1,ILK,ZYX,ITGA4,FLNA,MYO1G,CDC42 |
| GO:0007249 | canonical NF-kappaB signal transduction | 2.60E-04 | RHOC,HMOX1,LIMS1,ARRB2,ILK,FLNA,OPTN,CASP8,LITAF,PYCARD,UBE2N,TLE1,SLC44A2 |
| GO:0032801 | receptor catabolic process | 2.85E-04 | LGMN,ANXA2,CAPN1,UVRAG,PTPN1 |
| GO:0051495 | positive regulation of cytoskeleton organization | 3.70E-04 | GSN,S100A10,MTSS1,RHOC,HCK,FLNA,PLEK,PYCARD,CDC42,ADD3 |
| GO:0032970 | regulation of actin filament-based process | 3.77E-04 | GSN,S100A10,MTSS1,RHOC,ABRACL,CAPZB,HCK,TWF2,FLNA,ARPC5,PLEK,PYCARD,CDC42,ADD3 |
| GO:0051345 | positive regulation of hydrolase activity | 3.91E-04 | LGMN,GSN,S100A10,VSIR,TAX1BP3,RHOC,LIMS1,S1PR4,CASP8,PPP1R15A,PLEK,RASGRP2,PYCARD,CALM3 |
| GO:1902903 | regulation of supramolecular fiber organization | 4.25E-04 | GSN,S100A10,MTSS1,RHOC,CIB1,CAPZB,HCK,TWF2,FLNA,ARPC5,PLEK,PYCARD,CDC42,ADD3 |
| GO:0032956 | regulation of actin cytoskeleton organization | 4.55E-04 | GSN,S100A10,MTSS1,RHOC,CAPZB,HCK,TWF2,FLNA,ARPC5,PLEK,PYCARD,CDC42,ADD3 |
| GO:0001818 | negative regulation of cytokine production | 5.32E-04 | VSIR,SERPINB1,ANXA4,ARRB2,SRGN,TUSC2,TSPO,OAS1,ACP5,ATP2B1,PYCARD,KLF2 |
| GO:0034612 | response to tumor necrosis factor | 6.60E-04 | FOS,YBX3,LIMS1,CIB1,TUBA1A,ILK,SPPL2A,CASP8,ASAH1,PYCARD,KLF2 |
| GO:1903729 | regulation of plasma membrane organization | 6.99E-04 | GSN,S100A10,ANXA2,AHNAK |
| GO:0010038 | response to metal ion | 8.38E-04 | LGMN,CRIP1,FOS,HMOX1,TUBA1A,CPNE5,SUMO1,TCIRG1,TSPO,CASP8,RASGRP2,CALM3,PPP1CA |
| GO:0032233 | positive regulation of actin filament bundle assembly | 8.42E-04 | S100A10,MTSS1,RHOC,FLNA,PLEK,CDC42 |
| GO:1902905 | positive regulation of supramolecular fiber organization | 1.11E-03 | GSN,S100A10,MTSS1,RHOC,HCK,FLNA,PLEK,PYCARD,CDC42 |
| GO:0071356 | cellular response to tumor necrosis factor | 1.41E-03 | FOS,YBX3,LIMS1,CIB1,ILK,SPPL2A,CASP8,ASAH1,PYCARD,KLF2 |
| GO:0071800 | podosome assembly | 1.41E-03 | GSN,HCK,BIN2,DBNL |
| GO:0043112 | receptor metabolic process | 1.58E-03 | LGMN,ANXA2,CAPN1,OPTN,UVRAG,PTPN1 |
| GO:0007160 | cell-matrix adhesion | 1.58E-03 | S100A10,ADAM15,ITGB7,ITGB2,LIMS1,TLN1,CIB1,ILK,ZYX,ITGA4 |
| GO:0021762 | substantia nigra development | 1.89E-03 | YWHAH,DYNLL1,CALM3,CDC42,ATP5PF |
| GO:0008154 | actin polymerization or depolymerization | 2.04E-03 | GSN,CAPZB,HCK,TWF2,ARPC5,PLEK,PYCARD,ADD3,ARPC4 |
| GO:0008064 | regulation of actin polymerization or depolymerization | 2.14E-03 | GSN,CAPZB,HCK,TWF2,ARPC5,PLEK,PYCARD,ADD3 |
| GO:0051016 | barbed-end actin filament capping | 2.21E-03 | GSN,CAPZB,TWF2,ADD3 |
| GO:0030832 | regulation of actin filament length | 2.34E-03 | GSN,CAPZB,HCK,TWF2,ARPC5,PLEK,PYCARD,ADD3 |
| GO:0051017 | actin filament bundle assembly | 2.42E-03 | S100A10,MTSS1,RHOC,ZYX,FLNA,PDLIM1,PLEK,CDC42 |
| GO:0061572 | actin filament bundle organization | 2.60E-03 | S100A10,MTSS1,RHOC,ZYX,FLNA,PDLIM1,PLEK,CDC42 |
| GO:0043123 | positive regulation of canonical NF-kappaB signal transduction | 2.60E-03 | RHOC,HMOX1,LIMS1,ILK,FLNA,CASP8,LITAF,UBE2N,SLC44A2 |
| GO:0001667 | ameboidal-type cell migration | 2.79E-03 | LGMN,GLUL,ITGB7,ITGB2,HMOX1,CIB1,RHOB,ILK,LAMTOR2,ITGA4,PDLIM1,CDC42,CAP1 |
| GO:0043124 | negative regulation of canonical NF-kappaB signal transduction | 2.79E-03 | ARRB2,OPTN,CASP8,PYCARD,TLE1 |
| GO:0030834 | regulation of actin filament depolymerization | 2.93E-03 | GSN,CAPZB,TWF2,PLEK,ADD3 |
| GO:0010810 | regulation of cell-substrate adhesion | 3.14E-03 | S100A10,ADAM15,LIMS2,LIMS1,TLN1,CIB1,ILK,FLNA,CDC42 |
| GO:0010811 | positive regulation of cell-substrate adhesion | 3.14E-03 | S100A10,LIMS2,LIMS1,CIB1,ILK,FLNA,CDC42 |
| GO:0043244 | regulation of protein-containing complex disassembly | 3.14E-03 | GSN,CIB1,CAPZB,TWF2,UVRAG,PLEK,ADD3 |
| GO:0030041 | actin filament polymerization | 3.14E-03 | GSN,CAPZB,HCK,TWF2,ARPC5,PYCARD,ADD3,ARPC4 |
| GO:0045216 | cell-cell junction organization | 3.14E-03 | HOPX,LIMS2,MTSS1,RHOC,LIMS1,TLN1,FLNA,PDCD6IP,CDC42 |
| GO:1901879 | regulation of protein depolymerization | 3.35E-03 | GSN,CIB1,CAPZB,TWF2,PLEK,ADD3 |
| GO:0045428 | regulation of nitric oxide biosynthetic process | 3.52E-03 | ITGB2,DYNLL1,TSPO,ACP5,KLF2 |
| GO:0031638 | zymogen activation | 3.74E-03 | LGMN,S100A10,VSIR,ANXA2,CASP8 |
| GO:2001046 | positive regulation of integrin-mediated signaling pathway | 3.75E-03 | LIMS2,LIMS1,FLNA |
| GO:0097264 | self proteolysis | 3.75E-03 | LGMN,CAPN1,CASP8 |
| GO:0030042 | actin filament depolymerization | 3.93E-03 | GSN,CAPZB,TWF2,PLEK,ADD3 |
| GO:0080164 | regulation of nitric oxide metabolic process | 3.93E-03 | ITGB2,DYNLL1,TSPO,ACP5,KLF2 |
| GO:0043113 | receptor clustering | 4.19E-03 | GSN,ITGB7,ITGB2,ITGA4,FLNA |
| GO:0062098 | regulation of programmed necrotic cell death | 4.32E-03 | YBX3,TSPO,CASP8,ASAH1 |
| GO:0030833 | regulation of actin filament polymerization | 4.69E-03 | GSN,CAPZB,HCK,TWF2,ARPC5,PYCARD,ADD3 |
| GO:0051258 | protein polymerization | 4.96E-03 | GSN,MAP7D3,TUBA1A,CAPZB,HCK,TWF2,ARPC5,PYCARD,ADD3,ARPC4 |
| GO:0034109 | homotypic cell-cell adhesion | 4.96E-03 | TLN1,CD99,ILK,FLNA,PLEK,FIBP |
| GO:0006909 | phagocytosis | 5.00E-03 | GSN,FGR,ITGB2,HCK,TUSC2,BIN2,MYO1G,PYCARD,CDC42 |
| GO:1904406 | negative regulation of nitric oxide metabolic process | 5.37E-03 | DYNLL1,TSPO,ACP5 |
| GO:0045019 | negative regulation of nitric oxide biosynthetic process | 5.37E-03 | DYNLL1,TSPO,ACP5 |
| GO:0045785 | positive regulation of cell adhesion | 5.93E-03 | EBI3,S100A10,LIMS2,VSIR,ITGB2,LIMS1,CIB1,ILK,ITGA4,FLNA,PYCARD,CDC42,APBB1IP |
| GO:0043254 | regulation of protein-containing complex assembly | 6.27E-03 | HRK,GSN,RHOC,CAPZB,HCK,TWF2,SUMO1,ARPC5,PLEK,PYCARD,CDC42,ADD3 |
| GO:0032231 | regulation of actin filament bundle assembly | 6.31E-03 | S100A10,MTSS1,RHOC,FLNA,PLEK,CDC42 |
| GO:0042060 | wound healing | 7.20E-03 | CD151,S100A10,RHOC,HMOX1,ANXA2,TLN1,ILK,PABPC4,FLNA,AHNAK,PLEK,FIBP |
| GO:0048857 | neural nucleus development | 7.38E-03 | YWHAH,DYNLL1,CALM3,CDC42,ATP5PF |
| GO:0071248 | cellular response to metal ion | 7.73E-03 | LGMN,FOS,HMOX1,TUBA1A,CPNE5,SUMO1,TSPO,RASGRP2 |
| GO:0006809 | nitric oxide biosynthetic process | 8.10E-03 | ITGB2,DYNLL1,TSPO,ACP5,KLF2 |
| GO:0051693 | actin filament capping | 8.42E-03 | GSN,CAPZB,TWF2,ADD3 |
| GO:1901880 | negative regulation of protein depolymerization | 8.43E-03 | GSN,CIB1,CAPZB,TWF2,ADD3 |
| GO:0051100 | negative regulation of binding | 8.45E-03 | ADAM15,ARRB2,TLE5,SUMO1,ITGA4,PPP1CA |
| GO:0032091 | negative regulation of protein binding | 8.71E-03 | ADAM15,ARRB2,TLE5,ITGA4,PPP1CA |
| GO:0070527 | platelet aggregation | 8.71E-03 | TLN1,ILK,FLNA,PLEK,FIBP |
| GO:0072678 | T cell migration | 9.10E-03 | ITGB7,CD99,ITGA4,MYO1G,PYCARD |
| GO:0046209 | nitric oxide metabolic process | 9.90E-03 | ITGB2,DYNLL1,TSPO,ACP5,KLF2 |
| REAC:R-HSA-6798695 | Neutrophil degranulation | 6.02E-06 | GSN,ADGRE5,S100A11,FGR,SERPINB1,ITGB2,SERPINB6,ANXA2,CAPN1,DYNLL1,LAMTOR2,HMOX2,TCIRG1,ARPC5,PFKL,BIN2,ASAH1,TMBIM1,PYCARD,SLC44A2,CAP1,DBNL,GDI2 |
| REAC:R-HSA-76002 | Platelet activation, signaling and aggregation | 9.39E-05 | TLN1,ARRB2,RHOB,SRGN,GNG2,FLNA,PLEK,RASGRP2,TUBA4A,CDC42,APBB1IP,GNB2,CAP1,PTPN1,GNG5 |
| REAC:R-HSA-2132295 | MHC class II antigen presentation | 2.49E-03 | LGMN,TUBB6,IFI30,DYNLL1,AP1S2,TUBA1A,CAPZB,TUBA4A,CLTA |
| REAC:R-HSA-1632852 | Macroautophagy | 3.70E-03 | TUBB6,DYNLL1,TUBA1A,LAMTOR2,VDAC1,UVRAG,GABARAPL2,UBE2N,TUBA4A |
| REAC:R-HSA-195258 | RHO GTPase Effectors | 3.70E-03 | TUBB6,PPP1R14A,YWHAH,TAX1BP3,RHOC,DYNLL1,TUBA1A,RHOB,HIST1H2BK,FLNA,ARPC5,TUBA4A,CDC42,ARPC4 |
| REAC:R-HSA-446353 | Cell-extracellular matrix interactions | 4.82E-03 | LIMS2,LIMS1,ILK,FLNA |
| REAC:R-HSA-9612973 | Autophagy | 6.15E-03 | TUBB6,DYNLL1,TUBA1A,LAMTOR2,VDAC1,UVRAG,GABARAPL2,UBE2N,TUBA4A |
| REAC:R-HSA-8964616 | G beta:gamma signalling through CDC42 | 6.15E-03 | GNG2,CDC42,GNB2,GNG5 |
| REAC:R-HSA-9646399 | Aggrephagy | 9.57E-03 | TUBB6,DYNLL1,TUBA1A,UBE2N,TUBA4A |

| Supplemental Table S22. Detailed expression data for featured GO pathways in MYADM+ memory B cells | | | |
| --- | --- | --- | --- |
| Gene | Fold Change | | padj |
| GO:0007229 Integrin-Mediated Signaling (padj 2.4E-09) | | | |
| ADAM15 | | 2.00 | 7.41E-08 |
| CDC42 | | 1.34 | 9.32E-08 |
| FGR | | 1.91 | 1.56E-25 |
| FLNA | | 1.42 | 1.5E-03 |
| HCK | | 1.50 | 1.84E-06 |
| ILK | | 1.49 | 1.09E-05 |
| ITGA4 | | 1.43 | 7.0E-04 |
| ITGB2 | | 1.67 | 2.01E-16 |
| ITGB7 | | 1.74 | 9.25E-16 |
| LIMS1 | | 1.56 | 2.9E-04 |
| LIMS2 | | 1.82 | 2.64E-11 |
| PLEK | | 1.39 | 5.54E-07 |
| TLN1 | | 1.55 | 1.06E-06 |
| ZYX | | 1.48 | 4.7E-03 |
| GO0043122 Regulation of Canonical NK-KappaB Signaling (padj 1.1E-04) | | | |
| ARRB2 | | 1.50 | 1.7E-04 |
| CASP8 | | 1.42 | 9.62E-05 |
| FLNA | | 1.42 | 1.5E-03 |
| HMOX1 | | 1.61 | 7.1E-04 |
| ILK | | 1.49 | 1.09E-05 |
| LIMS1 | | 1.56 | 2.9E-04 |
| LITAF | | 1.42 | 2.40E-05 |
| OPTN | | 1.42 | 2.4E-03 |
| PYCARD | | 1.37 | 1.8E-04 |
| RHOC | | 1.65 | 2.5E-03 |
| SLC44A2 | | 1.32 | 6.8E-03 |
| TLE1 | | 1.33 | 3.2E-03 |
| UBE2N | | 1.34 | 1.3E-03 |
| GO0034612 Response to Tumor Necrosis Factor (padj 6.6E-04) | | | |
| ASAH1 | | 1.40 | 7.9E-03 |
| CASP8 | | 1.42 | 9.62E-05 |
| CIB1 | | 1.55 | 3.44E-07 |
| FOS | | 1.80 | 7.3E-03 |
| ILK | | 1.49 | 1.09E-05 |
| KLF2 | | 1.30 | 3.75E-05 |
| LIMS1 | | 1.56 | 2.9E-04 |
| PYCARD | | 1.37 | 1.8E-04 |
| SPPL2A | | 1.48 | 2.7E-03 |
| TUBA1A | | 1.51 | 4.39E-07 |
| YBX3 | | 1.79 | 1.51E-07 |
| REAC:R-HAS-2132295 MHC Class II Antigen Presentation Pathway (padj=2.49E-3) | | | |
| AP1S2 | | 1.53 | 4.92E-07 |
| CAPZB | | 1.51 | 2.22E-21 |
| CLTA | | 1.30 | 7.9E-03 |
| DYNLL1 | | 1.55 | 1.78E-12 |
| IFI30 | | 1.62 | 2.96E-14 |
| LGMN | | 2.90 | 3.16E-09 |
| TUBA1A | | 1.51 | 4.39E-07 |
| TUBA4A | | 1.34 | 5.3E-04 |
| TUBB6 | | 2.35 | 1.83E-17 |

| Supplemental Table S23. Differentially expressed genes for STAG3+ compared to STAG3- memory B cells using cut-offs FC > 1.25 and padj < 0.01 | | |
| --- | --- | --- |
| Gene | Fold Change | padj |
| CCND3 | 1.46 | 1.25E-12 |
| CD69 | 1.33 | 1.37E-03 |
| CXCR4 | 1.45 | 2.50E-15 |
| DDIT4 | 1.53 | 2.67E-09 |
| FCER2 | 1.47 | 4.87E-04 |
| GLO1 | 1.58 | 2.04E-10 |
| IL4R | 2.03 | 1.87E-07 |
| IRF8 | 1.29 | 1.87E-03 |
| MARCKSL1 | 1.48 | 1.26E-05 |
| P2RX5 | 1.44 | 1.70E-13 |
| PLPP5 | 1.39 | 1.52E-06 |
| RNGTT | 2.05 | 2.39E-09 |
| TCL1A | 1.85 | 4.96E-10 |
| ZFP36L1 | 1.29 | 7.34E-05 |
